# Synthesis, Structural Insights, and In Vitro Evaluation of Novel Silver Complexes Supported by Amantadine-Functionalized Bis(pyrazolyl)acetate Ligands as Anticancer Agents

**DOI:** 10.1021/acsomega.5c10692

**Published:** 2026-05-06

**Authors:** Maura Pellei, Michele De Franco, Carlo Santini, Miriam Caviglia, Jo’ Del Gobbo, Luca Barigelli, Fabio Del Bello, Wilma Quaglia, Chiara Battocchio, Giovanna Iucci, Iole Venditti, Carlo Meneghini, Simone Amatori, Valentina Gandin, Cristina Marzano, Alessandro Dolmella

**Affiliations:** † School of Science and Technology, Chemistry Division, 18959 University of Camerino, via Madonna delle Carceri (ChIP), 62032 Camerino, Italy; ‡ Department of Pharmaceutical and Pharmacological Sciences, University of Padova, via Marzolo 5, 35131 Padova, Italy; § School of Pharmacy, Medicinal Chemistry Unit, University of Camerino, via Madonna delle Carceri (ChIP), 62032 Camerino, Italy; ∥ Department of Science, Roma Tre University, Via della Vasca Navale 79, 00146 Roma, Italy

## Abstract

Bis­(pyrazol-1-yl)­acetic acid (HC­(pz)_2_COOH) and bis­(3,5-dimethyl-pyrazol-1-yl)­acetic acid (HC­(pz^Me2^)_2_COOH) were conjugated with amantadine to yield the ligands L^Ad^ and L^2Ad^, respectively. These chelating ligands were used for the synthesis of silver complexes 1–5, whose electronic and molecular structures were investigated by X-ray Photoelectron Spectroscopy (XPS) and Near Edge X-ray Absorption Fine Structure (NEXAFS) spectroscopy. The coordination geometry of the noble metal ion was assessed by combining X-ray Absorption Spectroscopy (XAS) data analysis with Density Functional Theory (DFT) modeling. The structures of the ligand L^Ad^ and of the complex [Ag­(L^2Ad^)­(PPh_3_)]­NO_3_·0.5CH_3_CN (3a) were determined by single-crystal X-ray diffraction analysis. The bis­(pyrazolyl)­acetamide ligand crystallizes in the I2/a space group and is similar to the 3,5-dimethyl analogue. The complex crystallizes in the P-1 space group and is the first reported example of a tetra-coordinated silver­(I) complex incorporating a tridentate bis­(pyrazolyl)­acetate ligand. The cytotoxicity potential of the new phosphane Ag­(I) complexes was tested on various human cancer cell lines, together with that of the two unfunctionalized analogs [Ag­(L^2OiPr^)­(PPh_3_)]­NO_3_ (6) and [Ag­(L^2OiPr^)­(PTA)]­NO_3_ (7), synthesized and studied for useful comparison. Notably, the novel complexes exhibited pronounced cytotoxic effects against the human-derived pancreatic cancer BxPC-3 cell line, both in 2D monolayers and in 3D spheroid models. Mechanistic studies revealed efficient intracellular accumulation of the complexes, likely favored by increased lipophilicity, and inhibition of thioredoxin reductase (TrxR), ultimately disrupting redox homeostasis and promoting oxidative stress, which are key contributors to their antiproliferative effects.

## Graphical abstract

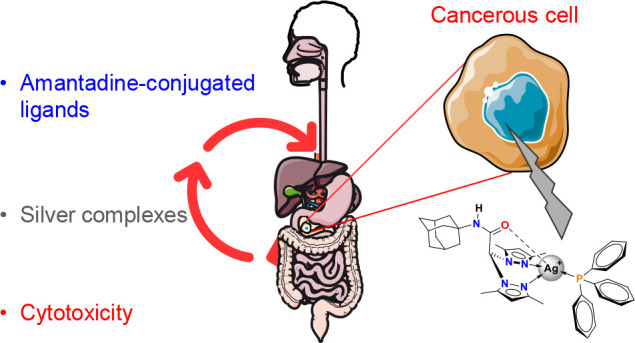

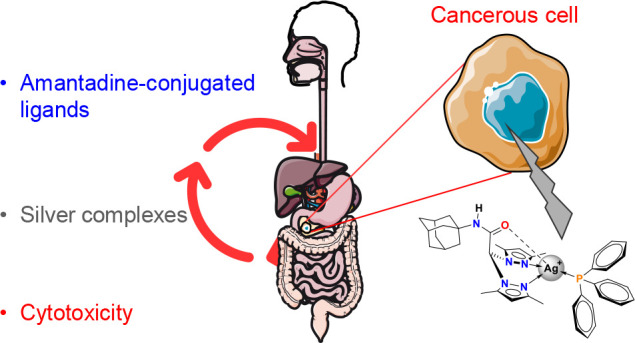

## 1 Introduction

Metallodrugs are pharmaceutical compounds containing metal ions as an essential component of their structure. Although they cover only a small fraction of pharmaceuticals currently available, some of them are among the most significant and widely used therapeutics in modern medicine. Unlike most conventional organic drugs, metallodrugs rely on the unique chemical properties of metals to exert their therapeutic effects, including variable oxidation states, geometry and coordination number, redox reactivity, and the ability to form complex structures with biomolecules.^1^

Group 11 metal complexes have demonstrated promising potential in this area, with numerous copper, silver, and gold complexes being explored for their antitumor properties.^2–4^ Among them, silver coordination compounds have gained recognition as promising therapeutic candidates in medical applications owing to their remarkable antibacterial, antifungal, antimalarial, antiparasitic, and anticancer properties.^3,5–9^ In cancer models, the antitumor effects of Ag­(I) complexes appear to involve biological pathways similar to those implicated in their antimicrobial activity,^3^ such as the improvement of reactive oxygen species (ROS), antimitochondrial effect, and apoptosis induction. They can disrupt redox balance, damage mitochondrial membranes, and activate intrinsic apoptotic pathways. Unlike platinum-based drugs, which primarily exert their effects through DNA cross-linking, silver complexes act through redox-mediated and protein-targeting mechanisms, thereby circumventing some of the resistance pathways associated with platinum drugs.^10,11^ However, little information is currently available regarding potential side effects on humans that may arise following exposure to Ag­(I) complexes, despite silver still being regarded as nontoxic to humans and other mammals. To the best of our knowledge, only a limited number of investigations have explored the impact of silver complexes on tumor xenograft models in mice, revealing either a reduction in tumor growth or induction of cell death.^12–14^

Ag­(I) complexes have been synthesized with a wide array of ligands for therapeutic applications, and ligand design plays a critical role in modulating their biological activity. Compounds incorporating N-heterocyclic carbenes, N-heterocycles, and phosphanes often show enhanced stability, cellular uptake, and bioactivity. These ligands can influence the lipophilicity, rate of release of the silver ions, charge distribution and redox proclivity of the complexes, thereby optimizing their pharmacological profiles.^3,7,15–24^ In this context, the adamantylamine moiety, exhibiting high lipophilicity and enhancing the cellular uptake of compounds into which it is incorporated,^25^ has been widely used in biologically active Ag­(I) complexes.^26–28^

Bis­(pyrazol-1-yl)­acetates ligands have also attracted considerable attention due to their κ^3^-N,N,O tripodal coordination behavior,^29–33^ and their metal complexes have been studied as ligands for metalloenzyme models relevant in the field of biochemistry.^34–38^ On the other hand, the carboxylic group enables easy functionalization, making these compounds valuable starting materials for the synthesis of ligands embedding a chelating core and a bioactive moiety, which are useful in biological studies^39–44^ and as catalysts.^45^ As part of our ongoing research focused on the chemical-biological properties of copper- and silver-based complexes incorporating ester or amide derivatives of bis­(pyrazol-1-yl)­acetate ligands,^39–44,46–51^ we recently described copper coordination compounds featuring a bis­(3,5-dimethyl-pyrazol-1-yl)­acetate ligand conjugated with an adamantane moiety, which showed promise as potential agents for glioblastoma therapy.^47^

Based on these observations, the main focus of the present research paper was to estimate the capability of ligands obtained by conjugating bis­(pyrazolyl)­acetates species with adamantylamine moiety to form Ag­(I) complexes, with potential applications in cancer therapy. Specifically, we herein present a study on the synthesis, characterization, and biological assessment of novel Ag­(I) complexes incorporating the newly designed N-(adamantan-1-yl)-2,2-di­(1H-pyrazol-1-yl)­acetamide (L^Ad^) ligand (compounds 1 and 2) or the already reported N-(adamantan-1-yl)-2,2-bis­(3,5-dimethyl-1H-pyrazol-1-yl)­acetamide (L^2Ad^)^47^ (compounds 3–5). The lipophilic triphenylphosphine (PPh_3_) and the hydrophilic 1,3,5-triaza-7-phosphaadamantane (PTA) were used as coligands to adjust the hydrophilicity and impart varying solubility characteristics to the synthesized complexes. For comparison, two new phosphane (PPh_3_ or PTA) silver­(I) complexes of the isopropyl ester derivative of bis­(3,5-dimethyl-pyrazol-1-yl)­acetic acid (L^2OiPr^) (6 and 7, respectively) have been synthesized to evaluate the biological effect of conjugation of the supporting ligands with amantadine.

The newly prepared Ag­(I) complexes 1–7, together with their respective free ligands, were tested for cytotoxic effects against a collection of human tumor cell lines, including cisplatin-resistant variants. Complexes 1–7 were further tested on three-dimensional spheroids generated from human BxPC-3 pancreatic adenocarcinoma cells, which exhibited high sensitivity to the cytotoxic effects of Ag­(I) compounds, underscoring their potential therapeutic relevance. Finally, more detailed mechanistic analyses were conducted to explain the molecular pathways involved in the anticancer effects observed in pancreatic carcinoma cells.

## 2 Materials and Methods

### 2.1 Chemistry

#### 2.1.1 Materials and General Methods

All chemicals employed in the preparation of the ligands and complexes were obtained from Sigma-Aldrich and used as received, without additional purification. Elemental analyses (C, H, N, S) were performed in-house with Fisons THERMO Fisher Flash 2000 instrument. Melting points (MP) were taken on a SMP3 Stuart Scientific Instrument. IR spectra were recorded from 4000 to 200 cm^–1^ with a PerkinElmer Frontier FT-IR Instrument. IR annotations used: wbr = weak broad, m = medium, mbr = medium broad, s = strong, sh = shoulder, vs = very strong, w = weak. ^1^H-, ^31^P- and ^13^C-NMR spectra were recorded on an Ascend 500 Bruker spectrometer (500.1 MHz for ^1^H, 202.5 MHz for ^31^P, 125.8 MHz for ^13^C). Chemical shifts, in ppm, for ^1^H- and ^13^C NMR spectra are relative to internal standard Me_4_Si. ^31^P NMR chemical shifts were referenced to an 85% H_3_PO_4_ standard. NMR annotations used: d = doublet, dbr = doublet broad, m = multiplet, s = singlet, sbr = singlet broad, t = triplet, AB q = AB quartet. Electrospray ionization-mass spectra (ESI-MS) were obtained in positive- and negative-ion (ESI-MS­(+) and ESI-MS(−)) mode on a Series 1100 MSD detector HP spectrometer, using a methanol or acetonitrile mobile phase. The compounds were dissolved in reagent-grade CH_3_OH, CH_3_CN or CH_3_CN/DMSO mixtures to prepare solutions with an approximate concentration of 0.1 mM. Aliquots of 1 μL were injected into the spectrometer via a HPLC HP 1090 Series II fitted with an autosampler. The pump delivered the solutions to the mass spectrometer source at a flow rate of 300 μL min^–1^, with nitrogen serving as both the drying and nebulizing gas. Capillary voltages were typically 4000 and 3500 V for the positive- and negative-ion mode, respectively. Identification of the main species detected in the ESI-MS experiments was supported by comparing the experimental and simulated isotope distribution patterns, the latter generated with the IsoPro 3.0 software. High-resolution mass spectra (HRMS) were obtained with electron spray ionization (HRMS-ESI) in the positive-ion mode using an Agilent Technologies 6545 Q-TOF LC/MS mass spectrometer. The molar conductivity (Λ_m_) of the acetonitrile solutions was determined using a Crison CDTM 522 conductivity meter at room temperature. Stability studies were performed acquiring ^1^H-NMR spectra on a Bruker Avance 300 MHz spectrometer (300.1 MHz for ^1^H) with a BBFO-z-ATMA probe.

The precursors HC­(pz)_2_COOH (LH)^31^ and HC­(pz^Me2^)_2_COOH (L^2^H)^30^ and the ligands L^2Ad^ ^47^ and L^2OiPr^ ^49^ were prepared by a method described in the literature. The spectroscopic data for ligand L^Ad^ and the complexes 1–7, including FT-IR, ^1^H-, ^13^C­{^1^H}-, ^31^P­{^1^H}-NMR and HR-MS spectra, are provided in the Supporting Information (Figures S1–S55).

#### 2.1.2 Synthesis of L^Ad^

Diisopropylethylamine (DIPEA, 0.294 g, 2.274 mmol) was added to a solution of amantadine (Ad, 0.157 g, 1.038 mmol) in DMF (10 mL). A solution of LH (0.200 g, 1.041 mmol) and 2-(1H-benzotriazole-1-yl)-1,1,3,3-tetramethylaminium tetrafluoroborate (TBTU, 0.334 g, 1.040 mmol) in DMF (10 mL) was then added dropwise to the reaction mixture, which was then stirred at room temperature for 20 h. The mixture was subsequently diluted with brine (30 mL) and extracted with ethyl acetate (2 × 30 mL). The combined organic layers were washed with distilled water (5 × 20 mL), dried over anhydrous Na_2_SO_4_, and concentrated under reduced pressure. Purification by flash chromatography, using cyclohexane/ethyl acetate (5:5) as the eluent, afforded ligand L^Ad^ as a white solid in 86% yield. MP: 132–134 °C. FT-IR (cm^–1^): 3281mbr (N–H); 3146w, 3114w, 3077w, 2916m, 2890w, 2849w (C–H); 1670vs (C=O); 1552s (C=C/C=N). ^1^H-NMR (CDCl_3_, 293 K): δ 1.70–2.10 (m, 15H, CH_Ad_), 6.36 (t, 2H, 4-CH_pz_), 6.87 (s, 1H, CHCO), 6.95 (sbr, 1H, NH), 7.64 (d, 2H, CH_pz_), 7.74 (d, 2H, CH_pz_). ^1^H-NMR (CD_3_CN, 293 K): δ 1.71–2.17 (m, 15H, CH_Ad_), 6.35 (t, 2H, 4-CH_pz_), 6.95 (sbr, 2H, CHCO and NH), 7.58 (d, 2H, CH_pz_), 7.79 (d, 2H, CH_pz_). ^13^C­{^1^H}-NMR (CDCl_3_, 293 K): 29.4 (s, CH_Ad_), 36.2 (s, CH_Ad_), 41.1 (s, CH_Ad_), 53.0 (s, CH_Ad_), 76.1 (s, CHCO), 107.0 (s, CH_pz_), 130.3 (s, CH_pz_), 141.4 (s, CH_pz_), 162.2 (s, C=O). ^13^C­{^1^H}-NMR (CD_3_CN, 293 K): 29.4 (s, CH_Ad_), 35.9 (s, CH_Ad_), 40.8 (s, CH_Ad_), 52.5 (s, CH_Ad_), 75.5 (s, CHCO), 106.6 (s, CH_pz_), 130.2 (s, CH_pz_), 140.6 (s, CH_pz_), 162.2 (s, C=O). ESI-MS­(+) (major positive ions, CH_3_OH), *m*/*z* (%): 326 (70) [L^Ad^ + H]^+^, 348 (100) [L^Ad^ + Na]^+^, 673 (50) [2L^Ad^ + Na]^+^. ESI-MS­(−) (major negative ions, CH_3_OH), *m*/*z* (%): 324 (100) [pz + Cl]^−^. Elemental Analysis (%) calculated for C_18_H_23_N_5_O: C 66.44, H 7.12, N 21.52; found: C 66.73, H 7.00, N 21.31.

#### 2.1.3 Synthesis of [Ag­(L^Ad^)­(PPh_3_)_2_]­NO_3_ (1)

Silver nitrate (0.500 mmol, 0.085 g) and triphenylphosphine (PPh_3_, 1.000 mmol, 0.262 g) were dissolved in CH_3_CN (30 mL) and stirred at room temperature for 2 h. Subsequently, ligand L^Ad^ (0.500 mmol, 0.163 g) was added, and the reaction mixture was stirred for an additional 24 h at room temperature in the dark. The resulting white suspension was filtered, and the filtrate was concentrated under reduced pressure to yield the complex in 82% yield. MP: 185–190 °C. Λ_m_ (CH_3_CN, 10^–3^ M, 293 K): 130.8 S cm^2^ mol^–1^. Solubility: CH_3_OH, CH_3_CN, CH_2_Cl_2_, CHCl_3_, acetone, DMSO. FT-IR (cm^–1^): 3265wbr (NH); 3052wbr, 2906m, 2849w (C–H); 1674m (C=O); 1479m, 1453w, 1434s (C=C/C=N); 1344m, 1307sbr (NO_3_). ^1^H-NMR (CDCl_3_, 293 K): δ 1.68 (s, 6H, CH_Ad_), 2.03–2.08 (d, 9H, CH_Ad_), 6.36 (t, 2H, CH_pz_), 7.34–7.46 (m, 31H, CH_Ar_ and CHCO), 7.59 (d, 2H, CH_pz_), 7.89 (d, 2H, CH_pz_). ^1^H-NMR (CD_3_CN, 293 K): δ 1.65–2.03 (m, 15H, CH_Ad_), 6.38 (t, 2H, CH_pz_), 7.22 (sbr, 1H, CHCO and NH), 7.36–7.54 (m, 30H, CH_Ar_), 7.61 (d, 2H, CH_pz_), 7.87 (d, 2H, CH_pz_). ^13^C­{^1^H}-NMR (CD_3_CN, 293 K): δ 29.4 (s, CH_Ad_), 35.9 (s, CH_Ad_), 40.8 (s, CH_Ad_), 52.6 (s, CH_Ad_), 74.8 (s, CHCO), 106.6 (s, CH_pz_), 129.2 (d, J_(C–P)_ = 9.9 Hz, PPh_3_), 130.8 (s, CH_pz_), 130.9 (d, J_(C–P)_ = 1.2 Hz, PPh_3_), 131.4 (d, J_(C–P)_ = 30.0 Hz, PPh_3_), 133.6 (d, J_(C–P)_ = 16.3 Hz, PPh_3_), 141.1 (s, CH_pz_), 162.2 (s, C=O). ^31^P­{^1^H}-NMR (CDCl_3_, 293 K): δ 10.61 (s). ^31^P­{^1^H}-NMR (CD_3_CN, 293 K): δ 10.26 (s). ^31^P­{^1^H}-NMR (CD_3_CN, 243 K): δ 8.69 (sbr). ESI-MS­(+) (major positive ions, CH_3_CN) *m*/*z* (%): 633 (100) [Ag­(PPh_3_)_2_]^+^, 696 (30) [Ag­(L^Ad^)­(PPh_3_)]^+^, 958 (5) [Ag­(L^Ad^)­(PPh_3_)_2_]^+^. ESI-MS(−) (major negative ions, CH_3_CN), *m*/*z* (%): 231 (100) [Ag­(NO_3_)_2_]^−^, 387 (5) [L^Ad^ + NO_3_]^−^. Elemental Analysis (%) calculated for C_54_H_53_AgN_6_O_4_P_2_: C 63.60, H 5.24, N 8.24; found: C 63.39, H 4.97, N 7.93. HR-MS [ESI, positive ion mode ESI-qTOF]: *m*/*z* for C_36_H_38_AgN_5_OP [M – PPh_3_]^+^ calcd: 694.1865. Found: 694.1869.

#### 2.1.4 Synthesis of [Ag­(L^Ad^)­(PTA)_2_]­NO_3_ (2)

Silver nitrate (0.500 mmol, 0.085 g) and 1,3,5-triaza-7-phosphaadamantane (PTA, 1.000 mmol, 0.158 g) were dissolved in CH_3_OH (20 mL) and the mixture was allowed to stir at room temperature for 3 h. Then, the ligand L^Ad^ (0.500 mmol, 0.163 g) was solubilized in CH_3_CN (10 mL) and added to the reaction mixture that was stirred for 24 h at room temperature, in the dark. The complex [Ag­(L^Ad^)­(PTA)_2_]­NO_3_ was recovered by filtration as a white solid in 86% yield. MP: 240–243 °C. Λ_m_ (CH_3_CN, 10^–3^ M, 293 K): 118.2 S cm^2^ mol^–1^. Solubility: CH_3_OH, DMSO. FT-IR (cm^–1^): 3281m (N–H); 3145w, 3114w, 3078w, 2916m, 2891sh, 2849w (C–H); 1671vs (C=O); 1553s, 1515w; 1444w, 1425w (C=C/C=N); 1392m; 1359s, 1343s, 1315s, 1290s (NO_3_); 1278s, 1255m, 1242s, 1212m, 1181m, 1170w, 1124m, 1107m, 1087s, 1049m, 1014s, 972vs, 948vs, 918w, 913m, 902m, 853w, 844m, 818s, 806s, 787vs, 774sh, 759vs, 747s, 716m. ^1^H-NMR (DMSO-d_6_, 293 K): δ 1.62 (s, 6H, CH_Ad_), 1.92 (d, 6H, CH_Ad_), 2.02 (s, 3H, CH_Ad_), 4.16 (d, 12H, NCH_2_P), 4.41–4.58 (AB q, 12H, NCH_2_N), 6.34 (t, 2H, CH_pz_), 7.18 (s, 1H, CHCO), 7.56 (d, 2H, CH_pz_), 7.80 (d, 2H, CH_pz_), 8.28 (s, 1H, NH). ^13^C­{^1^H}-NMR (DMSO-d_6_, 293 K): δ 29.2 (s, CH_Ad_), 36.3 (s, CH_Ad_), 41.0 (s, CH_Ad_), 50.8 (sbr, NCH_2_P), 52.2 (s, CH_Ad_), 72.6 (d, J_(C–P)_ = 5.7 Hz, NCH_2_N), 74.9 (s, CHCO), 107.0 (s, CH_pz_), 130.1­(s, CH_pz_), 140.4 (s, CH_pz_), 162.6 (C=O). ^31^P­{^1^H}-NMR (CD_3_CN, 293 K): δ – 86.97 (s). ^31^P­{^1^H}-NMR (CD_3_CN, 233 K): δ – 85.78 (dbr, ^1^J­(Ag–^31^P) = 207 Hz). ^31^P­{^1^H}-NMR (CD_3_OD, 293 K): δ – 83.29 (s). ^31^P­{^1^H}-NMR (CD_3_OD, 223 K): δ – 81.52 (sbr). ESI-MS­(+) (major positive ions, CH_3_CN/DMSO) *m*/*z* (%): 264 (15) [Ag­(PTA)]^+^, 421 (30) [Ag­(PTA)_2_]^+^, 591 (100) [Ag­(L^Ad^)­(PTA)]^+^. ESI-MS(−) (major negative ions, CH_3_CN/DMSO) *m*/*z* (%): 231 (100) [Ag­(NO_3_)_2_]^−^. Elemental Analysis (%) calculated for C_30_H_47_AgN_12_O_4_P_2_: C 44.51, H 5.85, N 20.76; found: C 44.94, H, 6.12, N 20.51. HR-MS [ESI, positive ion mode ESI-qTOF]: *m*/*z* for C_24_H_35_AgN_8_OP [M – PTA]^+^ calcd: 589.1722. Found: 589.1722.

#### 2.1.5 Synthesis of [Ag­(L^2Ad^)­(PPh_3_)]­NO_3_ (3)

Silver nitrate (0.500 mmol, 0.085 g) and triphenylphosphine (0.500 mmol, 0.132 g) were dissolved in CH_3_OH (30 mL) and the reaction mixture was stirred at room temperature for 2 h, after which ligand L^2Ad^ (0.500 mmol, 0.191 g) was added. Stirring continued for a further 24 h at room temperature in the dark. Diethyl ether (10 mL) and *n*-hexane (10 mL) were added to the solution, and a white solid was recovered by filtration. The white powder was washed with diethyl ether (10 mL) and *n*-hexane (10 mL) and dried under reduced pressure to give the complex [Ag­(L^2Ad^)­(PPh_3_)]­NO_3_ in 80% yield. MP: 216–217 °C. Λ_m_ (CH_3_CN, 10^–3^ M, 293 K): 150.1 S cm^2^ mol^–1^. Solubility: CH_3_OH, CH_3_CN, CH_2_Cl_2_, CHCl_3_, acetone, DMSO. FT-IR (cm^–1^): 3220wbr (N–H); 3049wbr, 2909m, 2850w (C–H); 1674s (C=O); 1376s, 1344sh, 1310vs (NO_3_). ^1^H-NMR (CD_3_CN, 293 K): δ 1.51–1.90 (m, 15H, CH_Ad_), 2.08 (s, 6H, CH_3_), 2.45 (s, 6H, CH_3_), 6.07 (s, 2H, CH_pz_), 7.00 (s, 1H, CHCO), 7.41 (sbr, 1H, NH), 7.48–7.58 (m, 15H, CH_Ar_). ^1^H-NMR (acetone-d_6_, 293 K): δ 1.60–2.52 (m, 27H, CH_Ad_ and CH_3pz_), 6.07 (s, 2H, CH_pz_), 7.53–7.66 (m, 16H, CH_Ar_ and CHCO), 9.25 (sbr, 1H, NH). ^13^C­{^1^H}-NMR (CD_3_CN, 293 K): δ 10.2 (s, CH_3pz_), 13.5 (s, CH_3pz_), 29.3 (s, CH_Ad_), 35.8 (s, CH_Ad_), 40.7 (s, CH_Ad_), 53.0 (s, CH_Ad_), 66.5 (s, CHCO), 106.7 (s, CH_pz_), 129.3 (d, J_(C–P)_ = 10.4 Hz, PPh_3_), 131.1 (d, J_(C–P)_ = 1.7 Hz, PPh_3_), 131.4 (d, J_(C–P)_ = 34.6 Hz, PPh_3_), 133.7 (d, J_(C–P)_ = 16.6 Hz, PPh_3_), 143.2 (s, C_pz_), 151.0 (s, C_pz_), 162.6 (s, C=O). ^31^P­{^1^H}-NMR (CD_3_CN, 293 K): δ 14.02 (s). ^31^P­{^1^H}-NMR (acetone-d_6_, 293 K): δ 14.87 (s). ^31^P­{^1^H}-NMR (CD_3_OD, 223 K): δ 15.15 (d, ^1^J­(^107^Ag–^31^P) = 649 Hz, and d, ^1^J­(^109^Ag–^31^P) = 734 Hz). ESI-MS­(+) (major positive ions, CH_3_CN), *m*/*z* (%), 633 (45) [Ag­(PPh_3_)_2_]^+^, 752 (100) [Ag­(L^2Ad^)­(PPh_3_)]^+^. ESI-MS(−) (major negative ions, CH_3_CN), *m*/*z* (%): 231 (50) [Ag­(NO_3_)_2_]^−^. Elemental Analysis (%) calculated for C_40_H_46_AgN_6_O_4_P (%): C 59.04, H 5.70, N 10.33; found: C 58.69, H 5.58, N 9.99. HR-MS [ESI, positive ion mode ESI-qTOF]: *m*/*z* for C_40_H_46_AgN_5_OP [M]^+^ calcd: 750.2491. Found: 750.2490.

From the slow crystallization of complex 3 in CH_3_CN solutions at 4 °C, crystals of sufficient quality for crystallographic analysis were obtained. The results of this analysis show that the crystallized item is an acetonitrile hemisolvate of complex 3, which has been indicated as complex 3a. Its spectroscopic data, including FT-IR, ^1^H-, ^13^C­{^1^H}-, ^31^P­{^1^H}-NMR and HR-MS spectra, are provided in the Supporting Information (Figures S51–S55).

#### 2.1.6 Synthesis of [Ag­(L^2Ad^)­(PPh_3_)_2_]­NO_3_ (4)

Silver nitrate (0.500 mmol, 0.085 g) and PPh_3_ (1.000 mmol, 0.262 g) were dissolved in CH_3_CN (30 mL) and the reaction mixture was stirred at room temperature for 2 h, after which ligand L^2Ad^ (0.500 mmol, 0.191 g) was added. Stirring continued for 24 h at room temperature in the dark. The solvent was then evaporated under reduced pressure, and the resulting residue was washed with diethyl ether (20 mL) and *n*-hexane (10 mL) and dried to give the complex [Ag­(L^2Ad^)­(PPh_3_)_2_]­NO_3_ in 90% yield. MP: 193–197 °C. Λ_m_ (CH_3_CN, 10^–3^ M, 293 K): 172.1 S cm^2^ mol^–1^. Solubility: CH_3_OH, CH_3_CN, CH_2_Cl_2_, CHCl_3_, DMSO, acetone. FT-IR (cm^–1^): 3243wbr (N–H); 3050wbr, 2907w, 2850w (C–H); 1673m (C=O); 1558w, 1479w, 1455w, 1434m (C=C/C=N); 1382m, 1360sh, 1308sbr (NO_3_). ^1^H-NMR (CDCl_3_, 293 K): δ 1.65–2.48 (m, 27H, CH_Ad_ and CH_3pz_), 5.87 (s, 2H, 4-CH_pz_), 7.30–7.43 (m, 32H, CH_Ar_, CHCO and NH). ^1^H-NMR (CD_3_CN, 293 K): δ 1.60–1.99 (m, 15H, CH_Ad_), 2.11 (s, 6H, CH_3pz_), 2.38 (s, 6H, CH_3pz_), 5.97 (s, 2H, 4-CH_pz_), 6.83 (s, 1H, CHCO), 7.26–7.50 (m, 31H, CH_Ar_ and NH). ^13^C­{^1^H}-NMR (CD_3_CN, 293 K): δ 10.4 (s, CH_3pz_), 13.0 (s, CH_3pz_), 29.4 (s, CH_Ad_), 35.9 (s, CH_Ad_), 40.8 (s, CH_Ad_), 52.5 (s, CH_Ad_), 69.1 (s, CHCO), 106.6 (s, CH_pz_), 129.1 (d, J_(C–P)_ = 9.6 Hz, PPh_3_), 130.7 (sbr, PPh_3_), 131.8 (d, J_(C–P)_ = 26.7 Hz, PPh_3_), 133.6 (d, J_(C–P)_ = 16.1 Hz, PPh_3_), 142.3 (s, C_pz_), 149.8 (s, C_pz_), 162.7 (s, C=O). ^31^P­{^1^H}-NMR (CDCl_3_, 293 K): δ 8.01 (s). ^31^P­{^1^H}-NMR (CD_3_CN, 293 K): δ 10.03 (s). ^31^P­{^1^H}-NMR (CD_3_CN, 233 K): δ 7.77 (d, ^1^J­(Ag–^31^P) = 318 Hz). ^31^P­{^1^H}-NMR (CD_3_OD, 223 K): δ 7.96 (d, ^1^J­(Ag–^31^P) = 338 Hz). ESI-MS­(+) (major positive ions, CH_3_CN), *m*/*z* (%): 633 (100) [Ag­(PPh_3_)_2_]^+^, 752 [Ag­(L^2Ad^)­(PPh_3_)]^+^. ESI-MS(−) (major negative ions, CH_3_CN), *m*/*z* (%): 231 (100) [Ag­(NO_3_)_2_]^−^. Elemental Analysis (%) calculated for C_58_H_61_AgN_6_O_4_P_2_: C 64.74, H 5.71, N 7.81; found: C 64.40, H 5.84, N 8.30. HR-MS [ESI, positive ion mode ESI-qTOF]: *m*/*z* for C_40_H_46_AgN_5_OP [M – PPh_3_]^+^ calcd: 750.2491. Found: 750.2490.

#### 2.1.7 Synthesis of [Ag­(L^2Ad^)­(PTA)]­NO_3_ (5)

Silver nitrate (0.500 mmol, 0.085 g) and PTA (1.000 mmol, 0.158 g) were dissolved in CH_3_OH (20 mL) and the mixture was stirred stirred at room temperature for 3 h. Then the ligand L^2Ad^ (0.500 mmol, 0.191 g) was added. The reaction mixture was stirred at room temperature for additional 24 h in the dark, after which the resulting white suspension was filtered off. Diethyl ether (10 mL) and *n*-hexane (10 mL) were added to the filtrate, and the yellow solid that precipitated was collected by filtration and dried under reduced pressure to give the complex [Ag­(L^2Ad^)­(PTA)]­NO_3_ in 58% yield. MP: 217–220 °C. Λ_m_ (CH_3_CN, 10^–3^ M, 293 K): 148.9 S cm^2^ mol^–1^. Solubility: CH_3_OH, CH_3_CN, CH_2_Cl_2_, CHCl_3_, DMSO. FT-IR (cm^–1^): 3260wbr (N–H); 3063w, 2908m, 2852m (C–H); 1675m (C=O); 1557m, 1447m, 1417m (C=C/C=N); 1311vs, 1290vs (NO_3_). ^1^H-NMR (DMSO-d_6_, 293 K): δ 1.61–2.37 (m, 27H, CH_Ad_ and CH_3pz_), 4.39 (s, 6H, NCH_2_P), 4.43–4.65 (AB q, 6H, NCH_2_N), 6.10 (s, 2H, CH_pz_), 6.92 (s, 1H, CHCO), 8.29 (s, 1H, NH). ^1^H-NMR (CD_3_CN, 293 K): 1.70–2.40 (m, 27H, CH_Ad_ and CH_3pz_), 4.38 (d, 6H, NCH_2_P), 4.53–4.67 (AB q, 6H, NCH_2_N), 6.03 (s, 2H, CH_pz_), 6.80 (s, 1H, CHCO), 7.00 (s, 1H, NH). ^1^H-NMR (CD_3_OD, 293 K): δ 1.73–2.42 (m, 27H, CH_Ad_ e CH_3pz_), 4.48 (s, 6H, NCH_2_P), 4.62–4.79 (AB q, 6H, NCH_2_N), 6.08 (s, 2H, CH_pz_), 6.91 (s, 1H, CHCO). ^13^C­{^1^H}-NMR (CD_3_CN, 293 K): δ 10.4 (s, CH_3pz_), 13.2 (s, CH_3pz_), 29.4 (s, CH_Ad_), 35.9 (s, CH_Ad_), 40.9 (s, CH_Ad_), 50.8 (d, J_(C–P)_ = 8.0 Hz, NCH_2_P), 52.6 (s, CH_Ad_), 68.4 (s, CHCO), 72.6 (d, J_(C–P)_ = 6.6 Hz, NCH_2_N), 106.5 (s, CH_pz_), 142.7 (s, C_pz_), 150.3 (s, C_pz_), 162.1 (s, C=O). ^31^P­{^1^H}-NMR (CD_3_CN, 293 K): δ – 80.97 (d, ^1^J­(Ag–^31^P) = 659 Hz). ^31^P­{^1^H}-NMR (CD_3_OD, 223 K): δ – 80.97 (d, ^1^J­(^107^Ag–^31^P) = 630 Hz, and d, ^1^J­(^109^Ag–^31^P) = 727 Hz). ESI-MS­(+) (major positive ions, CH_3_CN), *m*/*z* (%), 647 (100) [Ag­(L^2Ad^)­(PTA)]^+^, 871 (30) [Ag­(L^2Ad^)_2_]^+^. ESI-MS­(−) (major negative ions, CH_3_CN), *m*/*z* (%): 231 (100) [Ag­(NO_3_)_2_]^−^. Elemental Analysis (%) calculated for C_28_H_43_AgN_9_O_4_P: C 47.46, H 6.12, N 17.79; found: C 47.15, H 6.17, N 17.40. HR-MS [ESI, positive ion mode ESI-qTOF]: *m*/*z* for C_28_H_43_AgN_8_OP [M]^+^ calcd: 645.2348. Found: 645.2349.

#### 2.1.8 Synthesis of [Ag­(L^2OiPr^)­(PPh_3_)]­NO_3_ (6)

Silver nitrate (0.500 mmol, 0.085 g) and PPh_3_ (0.500 mmol, 0.132 g) were dissolved in CH_3_OH (50 mL) and the reaction mixture was stirred at room temperature for 2 h. Then, the ligand L^2OiPr^ (0.500 mmol, 0.145 g) was added, and the reaction was stirred for 24 h at room temperature, in the dark. A white suspension was filtered off and the obtained filtrate was dried under reduced pressure to give the complex [Ag­(L^2OiPr^)­(PPh_3_)]­NO_3_ in 82% yield. MP: 190–192 °C. Solubility: diethyl ether, CHCl_3_, DMSO, acetone. FT-IR (cm^–1^): 3133vw, 3052vw, 2972w, 2925w (C–H); 1747m (C=O); 1564m (C=C/C=N); 1323s, 1300s (NO_3_). ^1^H-NMR (CD_3_CN, 293 K): δ 1.05 (d, 6H, CH­(CH_3_)_2_), 2.16 (s, 6H, CH_3pz_), 2.41 (s, 6H, CH_3pz_), 4.84 (sept, 1H, CH­(CH_3_)_2_), 6.07 (s, 2H, 4-CH_pz_), 6,96 (s, 1H, CHCO), 7.47–7.58 (m, 15H, CH_Ar_). ^31^P­{^1^H}-NMR (CD_3_CN, 293 K): δ 13.59 (d, ^1^J­(Ag–^31^P) = 636 Hz). ^31^P­{^1^H}-NMR (CD_3_CN, 243 K): δ 12.00 (d, ^1^J­(^107^Ag–^31^P) = 616 Hz and d, ^1^J­(^109^Ag–^31^P) = 663 Hz). ESI-MS­(+) (major positive ions, CH_3_CN), *m*/*z* (%): 631 (15) [Ag­(PPh_3_)_2_]^+^, 659 (100) [Ag­(L^2OiPr^)­(PPh_3_)]^+^. ESI-MS­(−) (major negative ions, CH_3_CN), *m*/*z* (%): 231 (100) [Ag­(NO_3_)_2_]^−^. Elemental Analysis (%) calculated for C_33_H_37_AgN_5_O_5_P: C 54.86, H 5.16, N 9.69; found: C 54.12, H 5.04, N 10.22. HR-MS [ESI, positive ion mode ESI-qTOF]: *m*/*z* for C_33_H_37_AgN_4_O_2_P [M]^+^ calcd: 659.1705. Found: 659.1706.

#### 2.1.9 Synthesis of [Ag­(L^2OiPr^)­(PTA)]­NO_3_ (7)

Silver nitrate (0.500 mmol, 0.085 g) and PTA (0.500 mmol, 0.079 g) were dissolved in CH_3_OH (50 mL) and the reaction mixture was stirred for 3 h at room temperature. Then the ligand L^2OiPr^ (0.500 mmol, 0.145 g) was added. The reaction was stirred for 24 h at room temperature, in the dark and a white suspension was filtered off. The obtained filtrate was dried under reduced pressure to give the complex [Ag­(L^2OiPr^)­(PTA)]­NO_3_ in 59% yield. MP: 209–211 °C. Solubility: CH_3_OH, H_2_O, DMSO. FT-IR (cm^–1^): 3144vw, 2982w, 2922w (C–H); 1750s (C=O); 1645vw, 1564m (C=C/C=N); 1455m, 1422m, 1372m; 1349s, 1341s, 1318s (NO_3_). ^1^H-NMR (CD_3_OD, 293 K): δ 1.24 (d, 6H, CH­(CH_3_)_2_), 2.14 (s, 6H, CH_3pz_), 2.45 (s, 6H, CH_3pz_), 4.44 (s, 6H, NCH_2_P), 4.62–4.78 (AB q, 6H, NCH_2_N), 5.11 (sept, 1H, CH­(CH_3_)_2_), 6.09 (s, 2H, 4-CH_pz_), 7.23 (s, 1H, CHCO). ^1^HNMR (DMSO-d_6_, 293 K): δ 1.18 (d, 6H, CH­(CH_3_)_2_), 2.07 (s, 6H, CH_3pz_), 2.37 (s, 6H, CH_3pz_), 4.32 (s, 6H, NCH_2_P), 4.44–4.65 (AB q, 6H, NCH_2_N), 5.01 (sept, 1H, CH­(CH_3_)_2_), 6.07 (s, 2H, 4-CH_pz_), 7.38 (s, 1H, CHCO). ^31^P­{^1^H}-NMR (CD_3_OD, 293 K): δ – 82.07 (d, ^1^J­(Ag–^31^P) = 554 Hz). ^31^P­{^1^H}-NMR (CD_3_OD, 263 K): δ – 81.66 (d, ^1^J­(^107^Ag–^31^P) = 687 Hz and d, ^1^J­(^109^Ag–^31^P) = 620 Hz). ESI-MS­(+) (major positive ions, CH_3_CN), *m*/*z* (%): 397 (25) [Ag­(L^2OiPr^)]^+^, 554 (100) [Ag­(L^2OiPr^)­(PTA)]^+^. ESI-MS(−) (major negative ions, CH_3_CN), *m*/*z* (%): 231 (100) [Ag­(NO_3_)_2_]^−^. Elemental Analysis (%) calculated for C_21_H_34_AgN_8_O_5_P: C 40.85, H 5.55, N 18.15; found: C 41.03, H 5.59, N 17.99.

### 2.2 X-ray Crystallography

The solid-state, single-crystal X-ray diffraction analysis of the ligand L^Ad^ and of the silver­(I) complex [Ag­(L^2Ad^)­(PPh_3_)]­NO_3_·0.5CH_3_CN (3a) were performed on selected specimens found in vials where the named compounds were recrystallized from methanol (L^Ad^) or acetonitrile (3a). In the case of L^Ad^, a well-formed, colorless prism was fastened with Loctite glue to a glass capillary and mounted on the top of the goniometer head of a Rigaku-OD Gemini E diffractometer, equipped with a sealed tube Enhance Cu X-ray source and with an EOS CCD area detector. The raw diffraction data were collected at room temperature by means of the ω-scans technique, using graphite-monochromated Cu radiation (λ = 1.54184 Å). Data were acquired in a 1024 × 1024 pixel mode and 2 × 2 pixel binning using the CrysAlisPro software, Version 1.171.42.49;^52^ the same software was used also for reduction, finalization as well as to introduce the corrections for Lorentz and polarization effects. An absorption correction was performed empirically, by means of a multiscan approach, with the scaling algorithm SCALE3 ABSPACK, using equivalent reflections. The item used for the analysis of complex 3a was a small, colorless fragment chipped away from a much larger prismatic crystal, picked up with a nylon loop wet with a thin Paratone layer and then mounted on a Bruker D8 Venture diffractometer, equipped with an Incoatec IμS3.0­(EF) microfocus sealed tube Cu source with a Montel layer optics monochromator (λ = 1.54178 Å) and a Photon III C14 CPAD area detector, generously made available by the Department of Chemical Sciences of the University of Padova. The diffraction data of complex 3a were also obtained at room temperature, using the ω+ϕ scan technique. Data integration/reduction was made using a narrow-frame algorithm by means of SAINT software, Version 8.40B;^53^ data scaling and corrections for Lorentz, polarization, scan speed, background, including also an empirical correction for absorption, were performed with the SADABS program^54^ embedded in the APEX6 software.^55^

The unit cell parameters of L^Ad^ were obtained by the least-squares refinement of 13874 strong reflections selected during the whole experiment: those for compound 3a from the refinement of 9093 strong reflections in the 2θ range 3.893–72.097°. In both cases, crystal and equipment stability were monitored throughout the data collection, and no sample degradation, nor significant change in peak intensities were observed. In the case of L^Ad^, a manual data reduction was performed to account for small sample motions and to obtain better background treatment at the end of the data collection. The structures were solved by direct phasing (L^Ad^) or by the heavy-atom method (3a) and refined by full-matrix least-squares methods based on F_o_ ^2^ with the SHELXT^56^ and SHELXL^57^ programs through the OLEX2 program interface.^58^

During the refinement of complex 3a it became clear that the thermal displacement parameters of the C30, C31 and of the C37–C40 atoms in the C29/C34 and C35/C40 phenyl rings were quite high and that the involved atoms might be split. Similar considerations apply to the oxygen atoms of the nitrate counteranion. Attempts to treat these atoms as disordered over two arrangements gave unsatisfactory results, despite the introduction of DELU, SIMU and RIGU restraints. Since the chemical identity of the compound was not in question, we decided to leave the affected atom unsplit. The analysis of the electron density maps of 3a suggested also the fractional presence of a disordered acetonitrile molecule in the asymmetric unit. Again, despite several attempts, we could not obtain an acceptable model for this molecule, so its contribution has been removed by applying the MASK routine of OLEX2. The routine showed the presence in the asymmetric unit of a void of about 87 cubic angstroms, large enough to fit 0.5 acetonitrile molecules, as estimated by the electron count (10 electrons). In the last cycles of the refinement, all non-H atoms were allowed to vibrate anisotropically. With respect to the hydrogen atoms positions, in the case of L^Ad^ they were located by means of difference Fourier maps and refined freely; in complex 3a, hydrogen atoms were introduced in calculated positions and refined with a riding model, with their U_iso_ values restrained to 1.2/1.5 times the U_eq_ values of the pertinent “parent” atoms. A summary of crystal and data collection parameters for the two molecules is given in Table 1.

**Table 1:** Summary of Crystal and Data Collection Parameters for L^Ad^ and [Ag­(L^2Ad^)­(PPh_3_)]­NO_3_·0.5CH_3_CN (3a)

Compound	L^Ad^	3a
Empirical formula	C_18_H_23_N_5_O	C_40_H_46_N_6_O_4_PAg·0.5C_2_H_3_N
Formula weight	325.41	834.19
Temperature/K	298.6(8)	273.15(1)
Crystal system	monoclinic	triclinic
Space group	I 2/a	P – 1
a/Å	9.8874(2)	10.6087(2)
b/Å	9.44570(10)	13.1015(2)
c/Å	37.2177(6)	15.4829(3)
α/deg	90.0	107.2609(8)
β/deg	90.620(2)	97.5644(9)
γ/deg	90.0	94.0530(8)
Volume/Å^3^	3475.68(10)	2023.43(6)
Z	8	2
ρ_calc_ Mg/m^3^	1.244	1.369
μ/mm^–1^	0.645	4.758
F(000)	1392	866
Crystal size/mm^3^	0.80 × 0.32 × 0.12	0.24 × 0.20 × 0.12
Reflections collected	27148	93724
Independent reflections/R_int_	3429/0.0310	7851/0.0350
Restraints/parameters	0/310	3/473
Goodness-of-fit^a^ on F^2^	1.058	1.075
Final R (R_1_; wR_2_)^b^ indexes [I > 2σ (I)]	0.0399, 0.1079	0.0372, 0.1049
Largest diff. peak/hole/e Å^–3^	0.176/–0.169	0.560/–0.456

a Goodness-of-fit = [Σ (w (F_o_^2^ – F_c_^2^)^2^]/(N_obsvns_ – N_params_)]^1/2^, based on all data.

b R_1_ = Σ ||F_o_| – |F_c_||/Σ |F_o_|; wR_2_ = [Σw (F_o_^2^ – F_c_^2^)^2^/Σw (F_o_^2^)^2^]^1/2^.

Complete listings of atomic coordinates, bond lengths and angles, anisotropic thermal parameters are available as Supporting Information (Tables S1 and S2). The same information can also be obtained free of charge from the Cambridge Crystallographic Data Center (CCDC), via www.ccdc.cam.ac.uk/structures, with deposition numbers 2423299 for L^Ad^ and 2457262 for complex 3a.

### 2.3 Spectroscopic Methods

#### 2.3.1 X-ray Photoelectron Spectroscopy

XPS measurements were carried out using a custom designed spectrometer, described in previous studies^59^ and equipped with a nonmonochromatized Mg Ka X-ray source (Photon Energy = 1253.6 eV, pass energy = 25 eV, step 0.1 eV). For this experiment, photoelectrons emitted by C 1s, O 1s, N 1s, P 2p, Ag 3d core levels were detected on solid state samples (powders). All spectra were energy referenced to the C 1s signal of aliphatic C atoms having a binding energy BE 285.00 eV.^60^ Atomic ratios were calculated from peak intensities using Scofield’s cross-section values.^61^ Curve-fitting analysis was performed using Gaussian profiles as fitting functions, after subtraction of a polynomial background. For qualitative data, the BE values were referred to NIST database.^62,63^

#### 2.3.2 Near-Edge X-ray Absorption Fine Structure Spectroscopy

NEXAFS spectra of complexes 4 and 5 were recorded at the C and N K edges; experiments were carried out at the ELETTRA storage ring, at the BEAR beamline (Bending magnet for Emission Absorption and Reflectivity). The beamline is installed at the left exit of the 8.1 bending magnet exit; beamline optics deliver photons in the 5–1600 eV range. In these experiments, ammeters were used to measure the drain current from the sample. Investigated samples were prepared as thick films deposited onto Au/Si(111). In order to maximize signal intensity, spectra were collected at grazing (20°) incidence of the photon beam relative to the sample surface. In order to normalize the spectra to the incident photon flux, raw spectra were divided by the spectrum of a clean gold surface used as reference; subsequently, a straight line fitting the part of the spectrum below the edge was subtracted and the values recorded at 330.00 and 420.00 eV for C and N K edge spectra, respectively, were assessed to 1. The experimental C K edge spectra were energy referenced to the π*_C=O_ transition of the amide function of the L^2Ad^ ligand; for complex 4, the calibration was checked by the position of the intense π*_C=C_ peak of the benzene rings of the triphenylphosphine ligand.^64^ The N K edge spectra were energy referenced to the π*_2_ transition of the pyrazole rings.^45^

#### 2.3.3 X-ray Absorption Spectroscopy

X-ray absorption spectroscopy (XAS) measurements were carried out at the LISA-BM08 beamline^65^ at ESRF (European Synchrotron Radiation Facility, ESRF proposal ihmd58 and CERIC proposal #20232011) probing the Ag K edge of silver complexes 2, 4, and 5. The beamline optics featured a Si (111) Double Crystal Monochromator equipped with two mirrors, a collimating one and a focusing one, respectively before and after the DCM. The two mirrors also allow for rejection of higher order harmonics due to Pt coating. Samples were mixed with a suitable amount of cellulose and pressed into homogeneous pellets prior to the measurements. XAS spectra were collected in ambient Pressure and Temperature conditions in Transmission mode. The beamline is equipped with a gas-filled (Ar_(g)_) ionization chamber (I_0_) to measure the intensity of the incoming X-ray beam prior to the sample. The absorption signal is then measured by a second ionization chamber (I_1_) after the samples. Energy calibration was achieved by measuring the absorption signal from a pure Ag metal foil placed after the sample. Two additional nitrogen-filled ionization chambers (I_1_ and I_2_) positioned between the sample and the foil, and after the foil, respectively, were utilized to measure simultaneously the beam intensity transmitted through the sample and through the reference foil during XAS data acquisition. Nine scans were acquired for each sample, checked for energy calibration and merged to enhance the signal-to-noise ratio. For each scan the absorption spectra from samples and reference material were calculated as follows:

$$\alpha_{\mathrm{sample}}=\ln\left(\frac{I_{0}}{I_{1}}\right)\alpha_{\mathrm{ref}}=\ln\left(\frac{I_{1}}{I_{2}}\right)$$

Experimental spectra were treated along standard procedures for background subtraction (α′ = α_exp_ – α_pre_), edge jump normalization and bare atom background subtraction (α_b_)^66^ to extract the EXAFS structural signals $\chi_{\exp}\left(k\right)=\frac{\alpha^{\prime }-\alpha_{b}}{\alpha_{b}}$ . The edge energy (E_0_) is defined as the first inflection point (first maximum of the first derivative) for all spectra and is related to the energy scale of photoelectron wavenumber $k\left[Å-1\right]=\hbar^{-1}\sqrt{2m_{e}\left(E-E_{0}\right)}$ ­(where m_e_ is the mass of the electron, E­(eV) the X-ray energy).

Quantitative analysis of the EXAFS signals was achieved fitting the k^2^-weighted theoretical curves k^2^χ_th_ to the raw experimental data k^2^χ_exp_ (k). The theoretical curves χ_th_(k) were calculated starting from a reasonable guess geometry, which was then optimized via QuasiNewton-DTF optimization (as described in the Structural Models paragraph below). Photoelectron scattering amplitudes and phase functions were calculated using the FEFF8.4 ^67^ software and selected partial contributions χ_i_ were summed up to obtain the theoretical curves χ_th_(k). The χ_i_ were calculated using the standard EXAFS formula with Gaussian disorder approximation^68–70^ applying a not linear least-squares procedure implemented in the program FiteEXA.^66^ For each sample, the relevant single (SS) and multiple (MS) scattering contributions to the EXAFS signal were identified and those with similar path length and amplitude were grouped with the aim of minimizing the free variables within the fit of each sample.

### 2.4 Stability Studies

The water stability of the newly developed complexes was assessed by dissolving a known quantity of each compound (approximately 4 mg) in DMSO-d_6_ (∼0.1 mL), followed by dilution with D_2_O to a final volume of 0.5 mL. ^1^H-NMR spectra were acquired on a Bruker Avance 300 MHz spectrometer (300.1 MHz for ^1^H) with a BBFO-z-ATMA probe. Spectra were collected at different time points t = 0, 24, 48, and 72 h to observe the behavior of the complexes in aqueous solution.

### 2.5 Experiments with Cultured Human Cancer Cells

Complexes were freshly dissolved in DMSO before each experiment and added to the cell growth medium to a final solvent concentration of 0.5%, which is demonstrated not to significantly affect cell viability. Cisplatin was dissolved in 0.9% sodium chloride solution. MTT (3-(4,5-dimethylthiazol-2-yl)-2,5-diphenyltetrazolium bromide), p-nitrophenyl phosphate, cisplatin, auranofin and doxorubicin were obtained from Sigma Chemical Co, St. Louis, MO, USA.

#### 2.5.1 Cell Cultures

Human colon (HCT-15), pancreatic (BxPC-3), SCLC (U-1285) and breast (MCF-7) carcinoma cell lines along with noncancer Chinese Hamster Ovary (CHO) cells were obtained by American Type Culture Collection (ATCC, Rockville, MD, USA). Human ovarian 2008 and C13* cancer cells were kindly provided by Prof. G. Marverti (Dept. of Biomedical Science of Modena University, Italy). Cell lines were maintained in the logarithmic phase at 37 °C in a 5% carbon dioxide atmosphere using the following culture media containing 10% fetal calf serum (EuroClone, Milan, Italy), antibiotics (50 units per mL penicillin and 50 μg mL^–1^ streptomycin), and 2 mM l-glutamine: (i) RPMI-1640 medium (EuroClone) for HCT-15, BxPC-3, U-1285 and 2008 and C13* cells; (ii) DMEM (EuroClone) for MCF-7 cells; (iii) F-12 HAMs for CHO cells.

#### 2.5.2 MTT Assay

The growth inhibitory effect toward tumor cells was evaluated by means of MTT assay as previously described.^71^ IC_50_ values, the drug concentrations that reduce the mean absorbance at 570 nm to 50% of those in the untreated control wells, were calculated by the four-parameter logistic (4-PL) model. For amantadine pretreatment experiments, BxPC-3 cells (1 × 10^4^ /well) were treated with amantadine (50 μM) for 3 h and subsequently the cell medium was replaced with fresh one containing the tested compound (10 μM) for supplementary 72 h.

#### 2.5.3 Spheroid Cultures and Acid Phosphatase (APH) Assay

Spheroid models were obtained by seeding 2.5 × 10^3^ BxPC-3 human cancer cells per well in a round-bottom nontreated tissue culture 96-well plate (Greiner Bio-one, Kremsmünster, Austria) in phenol red free RPMI-1640 medium (Sigma Chemical Co., St. Louis, MO, USA) containing 10% fetal calf serum and supplemented with 20% methyl cellulose stock solution. An improved APH assay was used for assessing cell viability in 3D spheroids. IC_50_ values (drug concentrations that reduce the mean absorbance at 405 nm 50% of those in the untreated control wells) were calculated by 4-PL model.^72^

#### 2.5.4 Cellular Uptake

BxPC-3 cells (2.5 × 10^6^) were seeded in 75 cm^2^ flasks in growth medium (20 mL). After overnight incubation, the medium was replaced and the cells were treated with tested compounds for 24 h. Cell monolayers were washed with cold PBS, harvested, and counted. The samples were treated with highly pure nitric acid (Ag ≤ 0.01 μg kg^–1^, TraceSELECT Ultra, Sigma Chemical Co.) and transferred into a microwave Teflon vessel. Afterward, samples were mineralized by using a speed wave MWS-3 Berghof instrument (Eningen, Germany). After cooling, each mineralized sample was analyzed for copper content by means of a Varian AA Duo graphite furnace atomic absorption spectrometer (Varian, Palo Alto, CA; USA), at 324 nm. The calibration curve was obtained using known concentrations of standard solutions purchased from Sigma Chemical Co.

#### 2.5.5 Inhibition of TrxR

##### 2.5.5.1 Cell-Free TrxR1 Inhibition

The assay was performed in 0.2 M Na–K-phosphate buffer pH 7.4, containing 5 mM EDTA, 0.250 mM nicotinamide adenine dinucleotide phosphate (NADPH) and 75 nmol of TrxR1 (IMCO, Sweden) as previously described.^48^

##### 2.5.5.2 Inhibition of TrxR in Pancreatic Cancer Cells

BxPC-3 cells (1 × 10^6^) were grown in 75 cm^2^ flasks at the confluence and treated for 24 h with the metal complexes at equimolar concentrations (5 μM). Cell monolayers were harvested, washed with PBS, and centrifuged. Samples were lysed with RIPA buffer (Roche, Basel, Switzerland) and the TrxR assay was performed as above-described for isolated enzyme.

#### 2.5.6 ROS Production

ROS production was evaluated in BxPC-3 (10^4^ per well) grown for 24 h in a 96-well plate in RPMI medium without phenol red (Sigma Chemical Co.) as already described.^48^

#### 2.5.7 Quantification of Thiols

BxPC-3 cells (1.5 × 10^5^) were seeded in a six-well plate in growth medium (4 mL). After 24 h, cells were incubated for 24 h with IC_50_ concentrations of tested compounds. Afterward, the thiol content was estimated as formerly described.^73^

#### 2.5.8 Statistical Analysis

All values are the means ± SD of no less than three measurements starting from three different cell cultures. Multiple comparisons were made by ANOVA followed by the Tukey-Kramer multiple comparison test (*p < 0.05, **p < 0.01), using GraphPad software.

## 3 Results and Discussion

### 3.1 Synthesis and Characterization

Amantadine was reacted with LH in the presence of TBTU and DIPEA at room temperature for 20 h, yielding the new ligand L^Ad^ in 86% yield (Scheme 1).

**Scheme 1:**
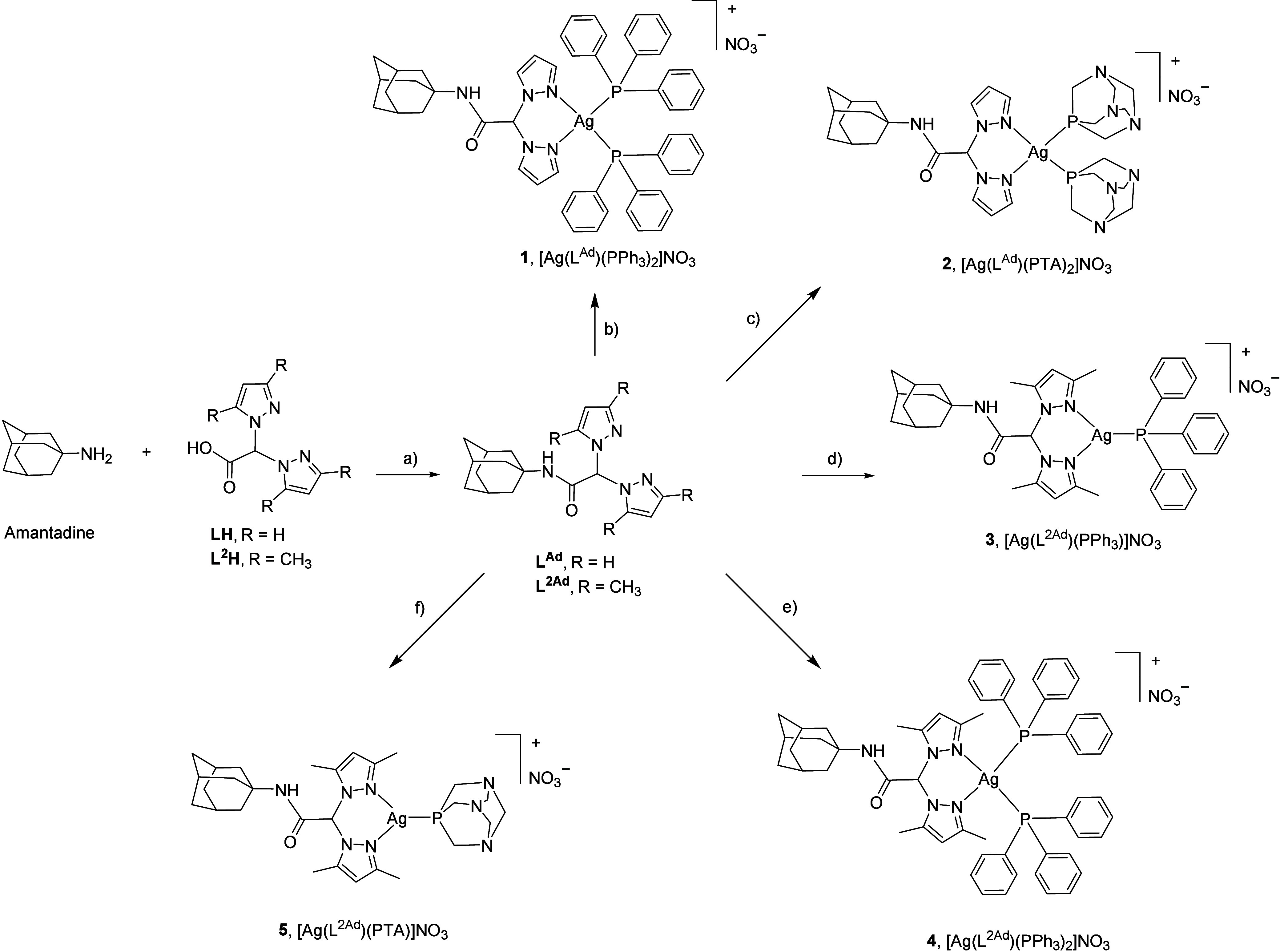
Reaction Scheme for the Synthesis of Ligand L^Ad^ and Complexes 1–5^a^

a Reagents: (a) TBTU, DIPEA, DMF, rt 20 h; (b) 1 eq AgNO_3_, 2 eq PPh_3_, CH_3_CN, rt 24 h; (c) 1 eq AgNO_3_, 2 eq PTA, CH_3_OH, rt 24 h; (d) 1 eq AgNO_3_, 1 eq PPh_3_, CH_3_CN, rt 24 h; (e) 1 eq AgNO_3_, 2 eq PPh_3_, CH_3_OH, CH_3_CN, rt 24 h; (f) 1 eq AgNO_3_, 2 eq PTA, CH_3_OH, rt 24 h.

The IR spectrum of a solid sample of L^Ad^ exhibited all the characteristic bands expected for the ligand: weak absorptions corresponding to C–H stretching were observed between 2849 and 3146 cm^–1^, while a broad peak at 3281 cm^–1^ was attributed to the N–H amide stretching. In addition, a very strong absorption band at 1670 cm^–1^ was observed, corresponding to the asymmetric stretching of the C=O group, consistent with typical amide functionality. The ^1^H- and ^13^C­{^1^H}-NMR spectra of L^Ad^ in CDCl_3_ and CD_3_CN solution displayed all predicted ligand signals. Furthermore, complete assignment of both ^1^H- and ^13^C­{^1^H}-NMR signals was achieved through a two-dimensional (2D) Heteronuclear Single Quantum Coherence (HSQC) experiment, which established proton-carbon single-bond correlations. The ^1^H-NMR spectrum displayed a single resonance pattern corresponding to the pyrazole rings, confirming their equivalence: a triplet at 6.36 ppm assigned to 4-CH_pz_ and two doublets at 7.64 and 7.74 ppm, corresponding to 5- and 3-CH_pz_. The ESI-MS study was carried out by dissolving the ligand in CH_3_OH and acquiring spectra in both positive- and negative-ion mode. The molecular structure of L^Ad^ was confirmed by the detection of molecular peaks at *m*/*z* 326, 348, and 673 in the positive-ions spectrum, due to the [L^Ad^ + H]^+^, [L^Ad^ + Na]^+^ and [2L^Ad^ + Na]^+^ adducts, respectively. Elemental analysis showed a strong correlation between the calculated and experimental values for C, H and N, further supporting the proposed structure.

The precursor L^2^H was conjugated to the drug amantadine to yield L^2Ad^, following our previously established procedures as reported in the literature,^47^ and the product was fully characterized.

The Ag­(I) complexes [Ag­(L^Ad^)­(PPh_3_)_2_]­NO_3_ (1) and [Ag­(L^Ad^)­(PTA)_2_]­NO_3_ (2) were synthesized via the reaction of silver nitrate, the ligand L^Ad^, PPh_3_ or PTA in a 1:1:2 stoichiometric ratio, respectively, using acetonitrile or methanol as solvent. Analogously, complex [Ag­(L^2Ad^)­(PPh_3_)_2_]­NO_3_ (4) was obtained from the reaction of AgNO_3_, PPh_3_ and the ligand L^2Ad^ in acetonitrile. The silver­(I) complexes [Ag­(L^2Ad^)­(PPh_3_)]­NO_3_ (3) and [Ag­(L^2Ad^)­(PTA)]­NO_3_ (5) were synthesized by reacting L^2Ad^, silver nitrate and either PPh_3_ or PTA, in a 1:1:1 molar ratio in methanol (Scheme 1). Attempts to synthesize the corresponding L^Ad^-based complex from AgNO_3_, PTA and L^Ad^ were unsuccessful, despite variation of solvents and stoichiometric ratios. All the complexes are soluble in methanol and DMSO. Complexes 1 and 3–5 additionally show solubility in acetonitrile, dichloromethane, and chloroform.

Conductivity measurements of complexes 1–5, which feature weakly coordinated NO_3_^–^ anions, align with their proposed ionic structures. Specifically, the molar conductance (Λ_m_) values for compounds 1–5, measured in acetonitrile, fall within the range of 118–172 S cm^2^ mol^–1^, consistent with their behavior as 1:1 electrolytes.

The IR spectra of the solid samples displayed all the characteristic bands associated with the chelating ligands and phosphane coligands. The Ag­(I) complexes exhibit strong absorptions due to the C=O stretching of the amide groups in the range 1671–1675 cm^–1^ with negligible shifts compared to the free ligand (1670 cm^–1^). Weak C–H stretching bands are observed in the 2849–3145 cm^–1^ region, while a series of strong bands in the 1290–1382 cm^–1^ region can be attributed to the stretching mode (ν_2_, ν_3_ and ν_4_ vibrations) of the NO_3_ group.^74,75^ These bands match those described earlier for related silver­(I) phosphane complexes.^48,76^ The ^1^H-NMR spectra of the Ag­(I) complexes, recorded in CDCl_3_, DMSO-d_6_, acetone-d_6_, CD_3_CN, or CD_3_OD at room temperature, a single set of resonances was observed for the pyrazole rings, indicating that the pyrazole protons are equivalent. Slight variation in the chemical shifts was observed, attributable to coordination with the silver center. In the triphenylphosphine complexes 1, 3 and 4, the PPh_3_ coligands showed characteristic multiplets in the range 7.26–7.66, with integrations consistent with a 1:1 (complex 3) and 1:2 (complexes 1 and 4) ligand-to-phosphane stoichiometry. Analogously, in the 1,3,5-triaza-7-phosphaadamantane complexes 2 and 5, the PTA coligands showed a doublet for the NCH_2_P protons at δ 4.16–4.48, and an AB quartet for the NCH_2_N protons at δ 4.41–4.79, consistent with literature reports. The ^31^P­{^1^H}-NMR spectra of complexes 1 and 2, supported by the L^Ad^ ligand and recorded in CD_3_CN at room temperature, showed signals at δ 10.26 and – 86.97, respectively, at lower field compared to those of the uncoordinated phosphanes PPh_3_ (δ = – 4.85) and PTA (– 102.07 ppm), confirming coordination to the Ag­(I) center. At 243 K the ^31^P­{^1^H}-NMR spectrum of [Ag­(L^Ad^)­(PPh_3_)_2_]­NO_3_ (1), recorded in CD_3_CN, shows a broad peak centered at δ 8.69 ppm. The ^31^P­{^1^H}-NMR spectrum of [Ag­(L^Ad^)­(PTA)_2_]­NO_3_ (2) in CD_3_CN at 233 K exhibited a broad doublet centered at δ – 85.78 ppm, with a ^1^J­(Ag–^31^P) coupling constant of 207 Hz, suggesting little to no phosphane exchange occurring at low temperature. The magnitude of this coupling constant is in line with values reported for analogous silver­(I) diphosphane complexes.^77–79^

The ^31^P­{^1^H}-NMR spectra of the triphenylphosphine Ag­(I) complexes 3 and 4, supported by the L^2Ad^ ligand and recorded in CD_3_CN at room temperature, gave signals in the range 10.03–14.02 ppm, while the PTA-containing complex 5 showed a resonance at δ – 80.97 ppm. All these signals are observed downfield relative to the chemical shifts of the free phosphanes PPh_3_ and PTA (δ = – 4.85 and – 102.07 ppm, respectively), confirming coordination to the silver center. The ^31^P­{^1^H}-NMR spectrum of complex 3 recorded in CD_3_OD at 223 K exhibited two doublets centered at 15.15 ppm, with ^1^J­(^107^Ag–^31^P) and ^1^J­(^109^Ag–^31^P) coupling constants of 649 and 734 Hz, respectively. Similarly, the spectrum of complex 5 under the same conditions showed two doublets centered at – 80.97 ppm, with ^1^J­(^107^Ag–^31^P) and ^1^J­(^109^Ag–^31^P) coupling constants of 630 and 727 Hz, respectively. The obtained values are in the same range as those described for related silver­(I) monophosphane complexes.^48,80^ The ^31^P­{^1^H}-NMR spectrum of complex 4, recorded in CD_3_OD at 223 K, revealed a doublet at 7.96 ppm, with a ^1^J­(Ag–^31^P) coupling constant of 338 Hz, in accordance. This is indicative of a slow phosphane exchange process and falls within the expected range for silver­(I) diphosphane species.^48,77–79^

The ESI-MS studies, conducted by dissolving the Ag­(I) complexes in CH_3_CN or CH_3_CN/DMSO mixtures and recording the spectra in both positive- and negative-ion modes, confirmed the formation of the PPh_3_ and PTA complexes as well as the presence of nitrate counterions. Specifically, the positive-ion ESI-MS spectra displayed peaks corresponding to the [Ag­(L^Ad^)­(PPh_3_)_2_]^+^, [Ag­(L^Ad^)­(PTA)]^+^, [Ag­(L^2Ad^)­(PPh_3_)]^+^, and [Ag­(L^2Ad^)­(PTA)]^+^ species, thereby confirming the successful synthesis of complexes 1–5. In addition, in the spectra of derivatives containing two PPh_3_ (complexes 1 and 4) or two PTA coligands (complex 2), peaks attributable to the fragments [Ag­(PPh_3_)_2_]^+^ and [Ag­(PTA)_2_]^+^ were also observed. In the negative-ion mode spectra, a peak at *m*/*z* 231, corresponding to the [Ag­(NO_3_)_2_]^−^ species, was consistently detected as the major ion for all complexes, further supporting the presence of nitrate as the counterion. Elemental analysis results were in good agreement with the calculated values, confirming both the proposed stoichiometry and the high purity of the isolated products.

The silver­(I) complexes [Ag­(L^2OiPr^)­(PPh_3_)]­NO_3_ (6) and [Ag­(L^2OiPr^)­(PTA)]­NO_3_ (7) were prepared by reaction of silver nitrate, the ligand L^2OiPr^ and triphenylphosphine or 1,3,5-triaza-7-phosphaadamantane in a 1:1:1 stoichiometric ratio, respectively, using methanol as solvent (Scheme 2).

**Scheme 2:**
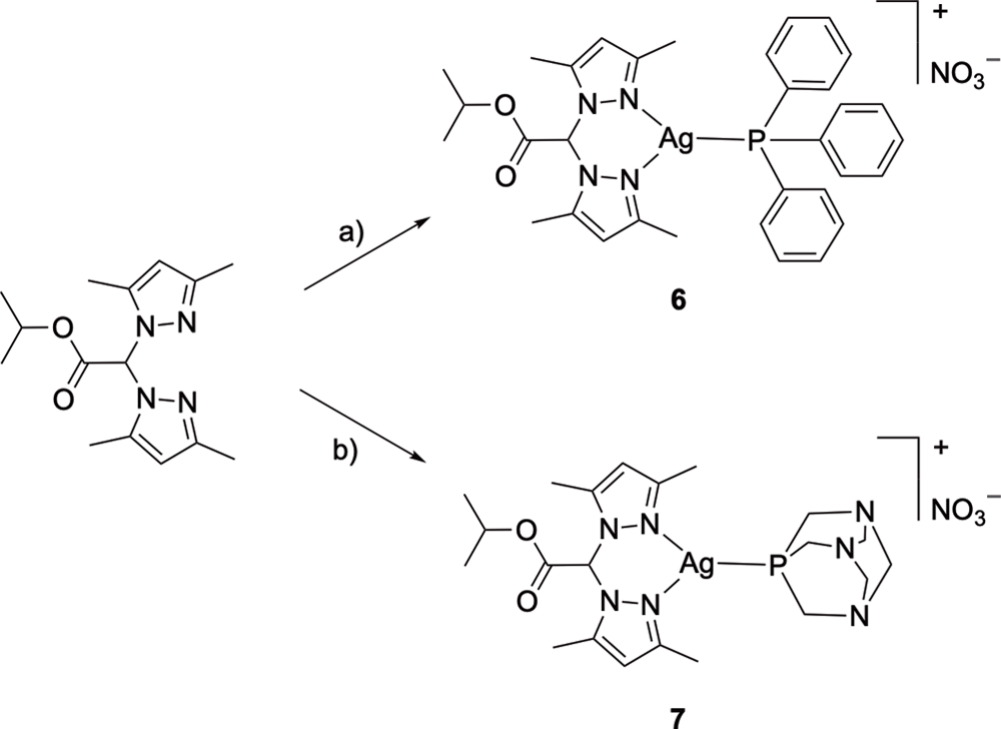
Reaction Scheme for the Synthesis of Complexes 6 and 7^a^

a Reagents: (a) 1 eq AgNO_3_, 1 eq PPh_3_, CH_3_OH, rt 24 h; (b) 1 eq AgNO_3_, 1 eq PTA, CH_3_OH, rt 24 h.

Complex 6 is soluble in diethyl ether, CHCl_3_, DMSO and acetone, while complex 7 is soluble in CH_3_OH, H_2_O and DMSO. The IR spectra of 6 and 7 revealed all characteristic bands attributable to both the chelating ligand and the phosphane coligand: weak C–H stretching absorptions were detected in the 2922–3144 cm^–1^ range, whereas the asymmetric C=O stretchings appeared at 1747–1750 cm^–1^, within the characteristic range for ester groups and showed no significant shift compared to the free ligand L^2OiPr^ (1748 cm^–1^).^45,48,50,81^ In addition, the strong bands in the 1300–1323 cm^–1^ region can be attributed to the ν_2_, ν_3_ and ν_4_ stretching mode vibrations of the NO_3_ group.^74,75^ The ^1^H NMR spectra of the Ag­(I) complexes 6 and 7, recorded in CD_3_CN and DMSO-d_6_ solution, respectively, displayed a single set of resonance signals for the pyrazole rings, suggesting that all pyrazole protons were equivalent, with a minor shift attributable to coordination with the metal center. The PPh_3_ and PTA moieties exhibited characteristic peak patterns at δ 7.47–7.58 and 4.32–4.65 ppm, respectively. The signal integration confirmed a 1:1 stoichiometric ratio between the ligand and the phosphane coligand. The ^31^P­{H}-NMR spectra of 6 and 7, recorded at room temperature in CD_3_CN and CD_3_OD solution, respectively, gave doublets at 13.59 and – 82.07 ppm, respectively, downfield shifted when compared with the uncoordinated phosphanes in the same solvents. At room temperature, the ^1^J­(Ag–^31^P) coupling constants are respectively of 636 Hz for compound 6 and 554 for compound 7, values comparable to those observed for similar silver­(I) monophosphane complexes.^48,77,82,83^ At 243 K, the spectrum of compound 6 in CD_3_CN shows two doublets, consistent with the coupling to the two silver isotopes (^1^J­(^107^Ag–^31^P) = 616 Hz and ^1^J­(^109^Ag–^31^P) = 663 Hz), values comparable to those reported for related silver­(I) monophosphane systems. The ^1^J­(^109^Ag–^31^P)/^1^J­(^107^Ag–^31^P) ratio matches well with the ^107^Ag/^109^Ag gyromagnetic ratio of 1.15. ESI-MS analyses, carried out by dissolving the Ag­(I) complexes in CH_3_CN and acquiring spectra in both positive- and negative-ion modes, confirmed the formation of the PPh_3_ and PTA adducts as well as the presence of nitrate counterions. Specifically, the positive-ion spectra displayed prominent peaks assignable to [Ag­(L^2OiPr^)­(PPh_3_)]^+^ and [Ag­(L^2OiPr^)­(PTA)]^+^ for complexes 6 and 7, respectively, while the negative-ion spectra for both species showed [Ag­(NO_3_)_2_]^−^ as the main signal.

### 3.2 X-ray Diffraction Studies

#### 3.2.1 Crystal Structure of Ligand L^Ad^

The structural characterization of the bis­(1H-pyrazol-1-yl)­acetyl-adamantan-1-amide ligand L^Ad^ reveals its similarity with the 3,5-dimethyl analogue (L^2Ad^) that we described in a recent work.^47^ Figure 1, obtained with the Mercury software,^84^ depicts an ORTEP^85^ representation of the solved structure. In the ligand, the N2/C5 ring and the adamantane moiety are syn-positioned with respect to the O1 amide oxygen, while the N4/C8 ring is positioned anticlinal. Not considering the adamantane group, the atoms in the molecule define three planes: those passing through the two pyrazolyl rings (N2–N3–C3–C4-C5 and N4–N5–C6–C7–C8, respectively, p1 and p2) and the one encompassing the acetamide group (C2–C1–O1–N1, p3). The three planes make with each other dihedral angles of 66.96° (p1-p3), 79.23° (p2-p3) and 79.22° (p1-p2). A search in the CCDC database^86^ for molecules incorporating a bis-pyrazolyl-acetamide residue returns 35 entries, most of them metal complexes in which the moiety chelates the metal.^44,87–103^

**Figure 1:**
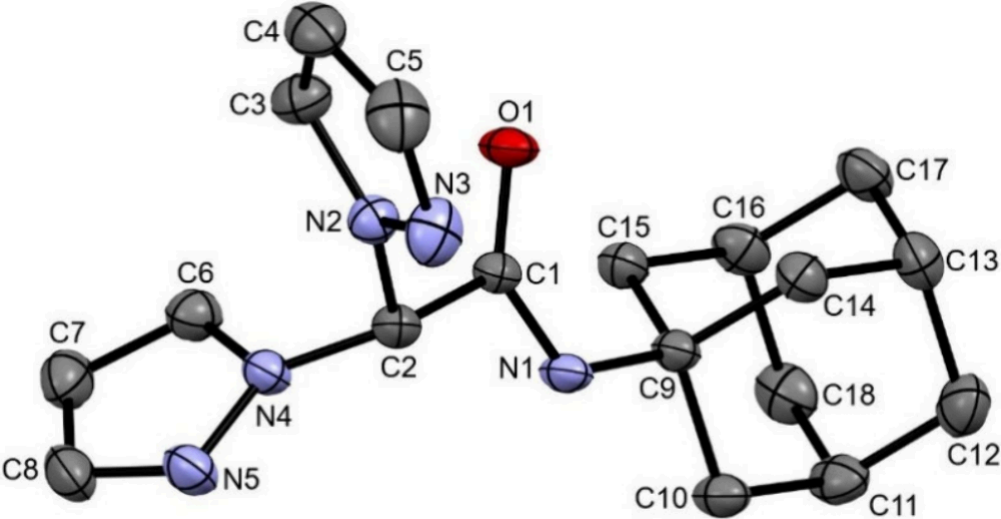
ORTEP drawing of L^Ad^, showing the selected numbering scheme. Hydrogen atoms omitted; thermal ellipsoids at the 30% probability. Selected bond lengths (Å): N2–N3 1.3478(16), N4–N5 1.3583(15), C2–N2 1.4477(16), C2–N4 1.4446(15), C1–C2 1.5521(16), C1–N1 1.3267(16), C1–O1 1.2196(15), N1–C9 1.4782(15). Selected bond angles (deg): C1–N1–C9 125.25(10), O1–C1–N1 125.60(11), N1–C1–C2 114.01(10), N2–C2–C1 110.72(9), N4–C2–C1 110.58(10), N4–C2–N2 111.10(10).

By comparing bond lengths and angles of reported structures with those of this work, it appears that the L^Ad^ ligand is quite similar (besides to the L^2Ad^ analogue) to 2,2-bis­(3,5-di-t-butyl-1H-pyrazol-1-yl)-N-phenylacetamide^92^ and bis­(N-t-butyl-2,2-bis­(3,5-dimethyl-1H-pyrazol-1-yl)­acetamide).^101^ In particular, the C1–O1 (1.2196(15) Å) and the C1–N1 (1.3267(16) Å) distances in L^Ad^ show, respectively, more and less double bond character when compared to the averages of reported structures (1.270 and 1.321 Å, respectively). Likewise, the C1–C2 and N1–C9 bonds in L^Ad^ (1.5521(16) and 1.4782(15) Å, respectively) have more single bond character (reported means of 1.507 Å and 1.435 Å, respectively). The C–C bond lengths in the adamantane residue lie instead in the narrow range of 1.52–1.53 Å. Due to a quite elongated *c* axis (>37 Å), the unit cell hosts 8 units of the ligand. However, few intermolecular contacts can be detected; they are listed in Table 2. A brief description of the contacts is given in the Supporting Information (Table S3 and Figure S56). Likely, the quite effective H-bond established by the O1 and H1 amide atoms prevents the formation of any other intramolecular contact and possibly also limits (looking at bond distances above) the tautomerism of the amide group.

**Table 2:** Nonbonding Interactions Parameters (Å and Degrees) For the Ligand L^Ad^

Donor (D)^a^	Contact (C)	Acceptor (A)	C····A	D····A	D–C–A^b^	Symmetry
N1	H1	O1	2.03	2.90	175.9	–1/2+x, – y, z
C3	H3	N4/C8^c^	2.73	3.58	129.7	–1/2+x, – y, z
C14	H14A	H10B	2.27	3.15	147.3	–1/2+x, – y, z

a Atom bound to contact atom.

b Donor–contact–acceptor angle.

c Geometric centroid of the N4/C8 or N2/C5 pyrazolyl rings.

#### 3.2.2 Crystal Structure of the Complex [Ag­(L^2Ad^)­(PPh_3_)]­NO_3_·0.5CH_3_CN (3a)

The structure of complex 3a was solved in the centrosymmetric P–1 space group (Figure 2).

**Figure 2:**
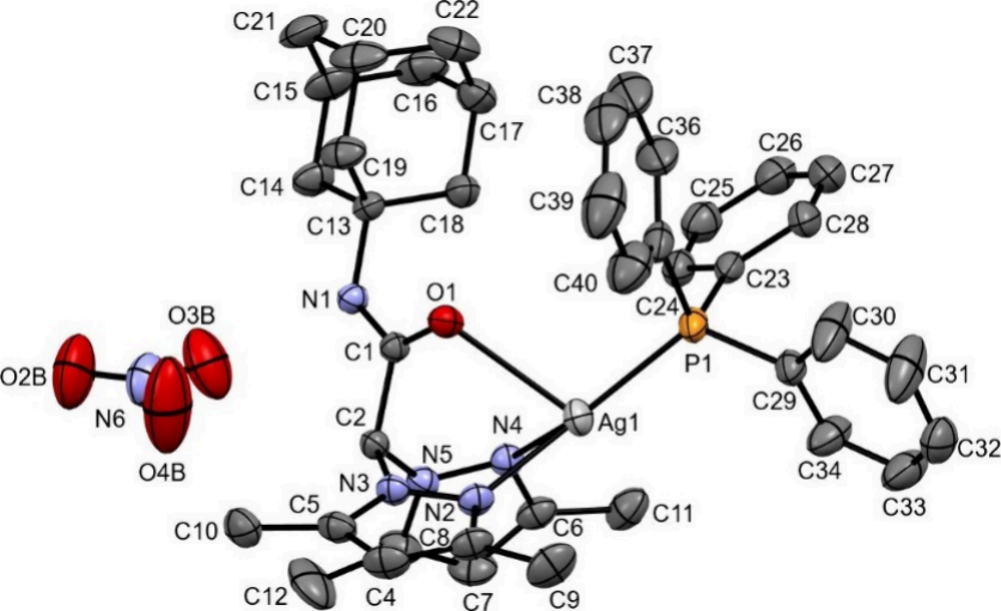
ORTEP drawing of complex 3a, showing the selected numbering scheme. Hydrogen atoms omitted; thermal ellipsoids at the 30% probability. Selected bond lengths (Å): Ag1–P1 2.3417(7), Ag1–N2 2.250(2), Ag1–N4 2.377(2), Ag1–O1 2.5684(19), N2–N3 1.367(3), N4–N5 1.367(3), C1–N1 1.322(3), C1–O1 1.222(3), C13–N1 1.480(3). Selected bond angles (deg): N2–Ag1–N4 81.15(8), N2–Ag1–P1 146.04(6), N4–Ag1–P1 132.69(6), O1–Ag1–P1 104.23(4), O1–Ag1–N2 73.89(7), O1–Ag1–N4 81.93(6), O1–C1–N1 125.4(2).

Disregarding the fractional acetonitrile molecule (see Materials and Methods), the asymmetric unit contains one complex [Ag­(L^2Ad^)­(PPh_3_)]^+^ cation and its nitrate counteranion. The latter is hold in position by efficient interactions involving the O3B, O4B atoms of the nitrate (Figure S57). In the complex, the silver atom is bound by the unidentate triphenylphosphine ligand and by the tridentate ligand L^2Ad^, in a markedly distorted tetrahedral environment. The distortion is accounted for by the values of the τ_4_ and τ_4_′ indexes^104,105^ of 0.58 and 0.53, respectively, compared to 1.00 for an ideal tetrahedron.

An inspection of the bond angles about silver also shows that the N2–Ag1–P1, N4–Ag1–P1 and N2–Ag1–N4 angles measure 146.04(6)°, 132.69(6)° and 81.15(8)°, respectively. The N2, N4, P1, Ag1 atoms are in fact coplanar within 0.03 Å. Such disposition resembles that found in (distorted) trigonal planar silver­(I) complexes showing an N,N,P-donor set made by a PPh_3_ and an N,N′-bidentate ligand found in the CCDC repository.^106–111^ The mean values for the named angles in reported compounds are 137.9°, 136.8° and 83.5°, respectively; our data compare well with those reported for silver­(I) complexes of azadipyrromethene and triphenylphosphine.^107,108^ However, it must be noted that although the Ag1–O1 distance of 2.5684(19) Å is very different from the Ag1–N2, Ag1–N4, Ag1–P1 bond lengths (2.250(2), 2.377(2), 2.3417(7) Å, respectively) it remains below the sum of the van der Waals radii for the Ag and O atoms (4.03 Å)^112^ and within the range (2.17–2.93 Å, mean 2.505 Å) for about 400 tetracoordinate silver­(I) complexes in the CCDC database. Hence, O1 oxygen effectively belongs to the silver coordination environment. Also notably, despite a wide number of scorpionate and pseudoscorpionate transition metal complexes deposited in the CCDC database, few tetra-coordinate mononuclear Group 11 derivatives showing the N,N,O,P-donor set have been reported so far;^113–115^ none of these contains a tridentate bis­(pyrazolyl) ligand. So, to the best of our knowledge, this is the first reported example of a silver­(I) tetra-coordinate complex of this kind.

By comparing bond distances and angles around silver (mean values of Ag–O, Ag–N, Ag–P distances in known similar compound are 2.545, 2.325, 2.352 Å, respectively) in silver­(I) tris­(pyrazolyl)­methanesulfonate and triphenylphosphine or 1,3,5-triaza-7-phosphadamantane complexes,^113^ where a tris­(pyrazolyl)­methanesulfonate ligand replaces the bis­(pyrazolyl)­acetamide one, show the closest similarity with complex 3a. It is worth noting that in all these cases the two Ag–N distances are slightly different, with the longest one comparable or sometimes longer than the Ag–P length and the shorter one always associated with the widest N–Ag–P bond angle.

To gain further insights about the silver­(I) coordination environment, a range separated-GGA hybrid HF-DFT calculation was also performed with the 6–31G* basis set and the ωB97X-D functional on the isolated complex cation by means of the Spartan software,^116^ allowing for optimization of the input X-ray coordinates. The optimization substantially preserves the overall reciprocal arrangement of the molecular moieties, however, the calculated bond distances and angles about silver change. In particular, the N2–Ag1–P1, N4–Ag1–P1, N2–Ag1–N4 angles assume the values of 141.07°, 138.35°, 80.55°, respectively; the Ag1–O1, Ag1–P1, Ag1–N2, Ag1–N4 bond lengths become 2.878 Å, 2.410 Å, 2,334 Å, 2.411 Å, respectively. Accordingly, the computation over the isolated cation maintains the difference between the Ag–N distances and the similarity between the longest Ag–N and the Ag–P lengths, while markedly diminishing the relevance of the Ag–O bond. This result suggests that packing forces in the solid state might bolster the effectiveness of the Ag–O bond.

Upon coordination, the L^2Ad^ ligand forms three six-membered rings; all of them show a twist-boat arrangement, with the C2 and Ag1 atoms taking the “stern” and “prow” positions, respectively. These atoms deviate from the mean plane of the remaining four (N3, N2, N4, N5 (I); N3, N2, O1, C1 (II); N5, N4, O1, C1 (III)) in the range of 0.51­(II)-0.66­(I) Å for C2, 0.94­(I)-1.42­(II) Å for Ag1. The twist follows the differences of bond lengths in the Ag coordination environment that makes the tridentate “bite” inhomogeneous. In the bound ligand, the orientation of the N2/C5, N4/C8 pyrazolyl rings and of the adamantane moiety with respect to the O1 amide oxygen matches that seen before for the isolated L^Ad^ ligand, that is, syn, anticlinal, syn, respectively. The mean planes passing through the N2/C4 and N4/C8 pyrazolyl rings make with each other a dihedral angle of 77.89°, and angles of 61.99°, 77.51°, respectively, with the mean plane incorporating the C2, C1, N1, O1 atoms of the amide moiety. As for the bond lengths in L^2Ad^, they do not differ from those of the L^Ad^ ligand, except a limited rearrangement of the C1–O1 and C1–N1 distances, which slightly elongate (1.222(3) Å) and shorten (1.322(3) Å) again due to O1 taking part in Ag coordination. Given its saline nature, the packing diagram of 3a reveals a 3D nonbonding interaction network; the more efficient contacts are listed in Table 3. A description of these interactions is given in the Supporting Information (Table S4 and Figures S56–S63).

**Table 3:** Nonbonding Interactions Parameters (Å and Degrees) for the Complex 3a

Donor (D)^a^	Contact (C)	Acceptor (A)	C····A	D····A	D–C–A^b^	Symmetry
N1	H1	O3B	2.05	2.90	171.0	x, y, z
C2	H2	O4B	2.41	3.33	156.1	x, y, z
C10	H10A	O4B	2.54	3.24	129.4	x, y, z
C10	H10B	O1	2.56	3.43	151.0	2–x, 1–y, 1–z
C26	H26	O2B	2.36	3.48	153.3	2–x, 1–y, 2–z
C10	H10A	N2/C5^c^	2.62	3.53	117.5	2–x, 1–y, 1–z
C7	H7	H31	2.32	3.23	164.5	x, –1+y, z
C9	H9A	Ag1	3.14	4.08	166.0	1–x, 1–y, 1–z

a Atom bound to contact atom.

b Donor–contact–acceptor angle.

c Geometric centroid of the N4/C8 or N2/C5 pyrazolyl rings.

### 3.3 XPS and NEXAFS Investigation of the Molecular and Electronic Structure

#### 3.3.1 XPS Analysis of Ag­(I) Coordination Compounds

Coordination compounds 1 and 4, differing for only two methyl groups on the pyrazolyl rings, are very similar from the point of view of the information about molecular and electronic structure attainable by XPS data analysis; similarly, complex 2 is structurally analogous to 1 and 4, but for the two PTA ligands instead of the two PPh_3_. Then, here we will discuss in detail the XPS data collected on sample 4, that can be considered representative of the three Ag­(I) coordination compounds.

Complexes 5 and 3 bearing only one phosphine ligand, either PTA (5) or PPh_3_ (3), are structurally similar therefore, XPS spectra measured for 5 will be taken as representative of both coordination compounds. As shown in Figure 3, C 1s, N 1s, P 2p and Ag 3d spectra of 4 and 5 are similar and fully confirm the molecular stability of L^Ad^ and phosphine ligands upon coordination with silver ions, as well as the Ag­(I) oxidation state. More in detail, C 1s spectra (first row of Figure 3) have three components respectively assigned to aliphatic and aromatic C–C bonds (285.0 eV BE), C–N (286.5 eV BE) and carbon atoms in NHCO groups (288 eV BE). As expected, the spectral component related to C–C/C=C groups in 4 is more intense than the same component in 5 due to the presence of two triphenylphosphines instead of one PTA ligand. As for N 1s spectra, two peaks can be individuated for all coordination compounds, in analogy with analogous complexes of Cu­(I/II),^46,47^ due to amine-like and amide-like N (400 eV), and imine-like N (401 eV BE). P 2p spectra (third row of Figure 3) fully confirm the stability of the phosphine ligands, showing a single pair of spin–orbit components due to the P atoms of PPh_3_ (4) and PTA (5) (P 2p_3/2_ BE = 131–131.5 eV).^62,63^ Finally, Ag 3d core-level spectra have a single pair of spin–orbit components at BE values fully consistent with the expected (+1) oxidation state (Ag 3d_5/2_ BE = 368.5 eV),^63^ confirming the stability of the investigated compounds.

**Figure 3:**
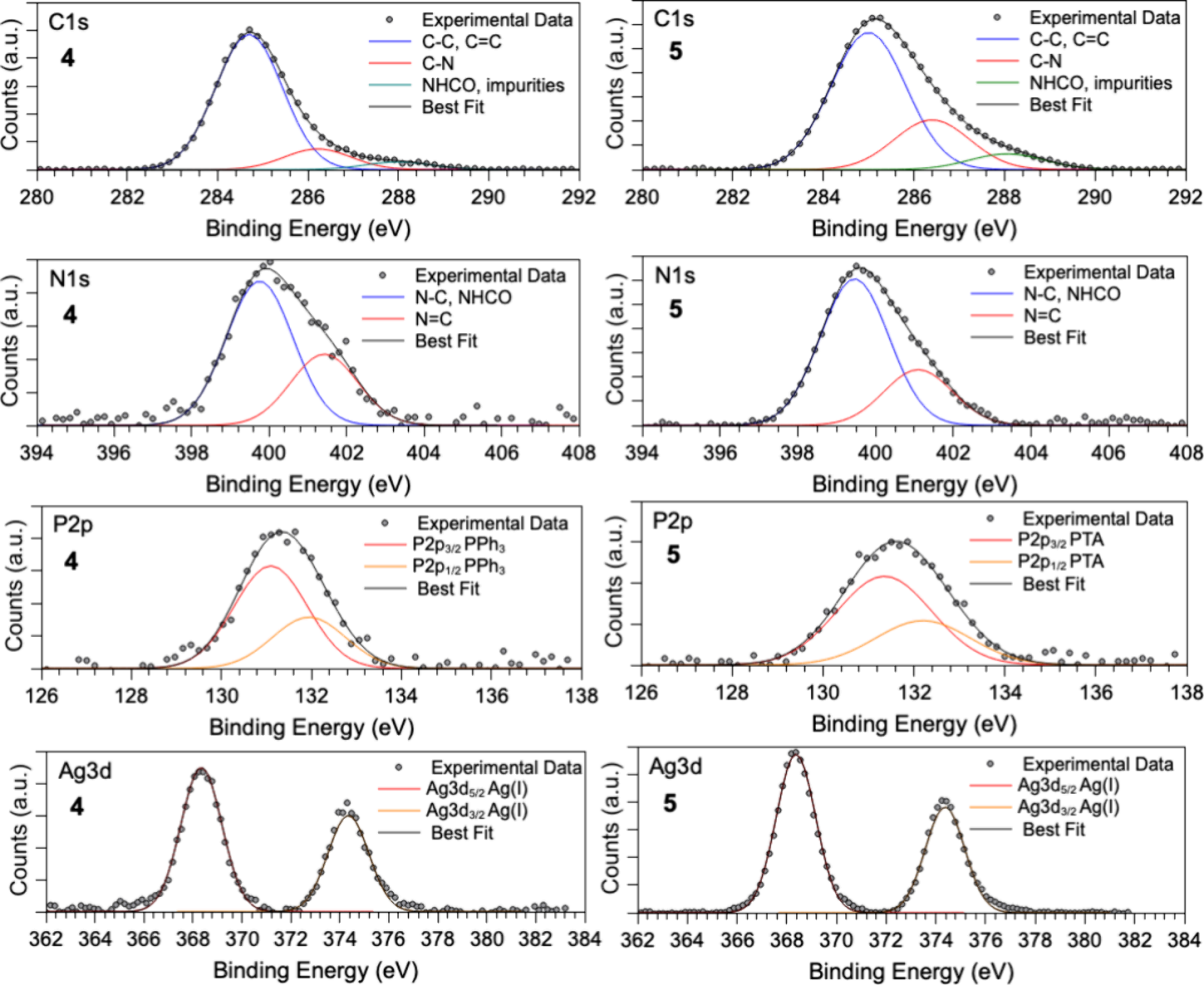
C 1s, N 1s, P 2p and Ag 3d core-level spectra collected for coordination compound 4 (considered representative of complexes 1, 2 and 4) (left) and 5 (representative for complexes 3 and 5) (right).

### 3.4 NEXAFS Spectroscopy

The C K edge and N K edge NEXAFS spectra of complexes 4 and 5 are shown in Figure 4.

**Figure 4:**
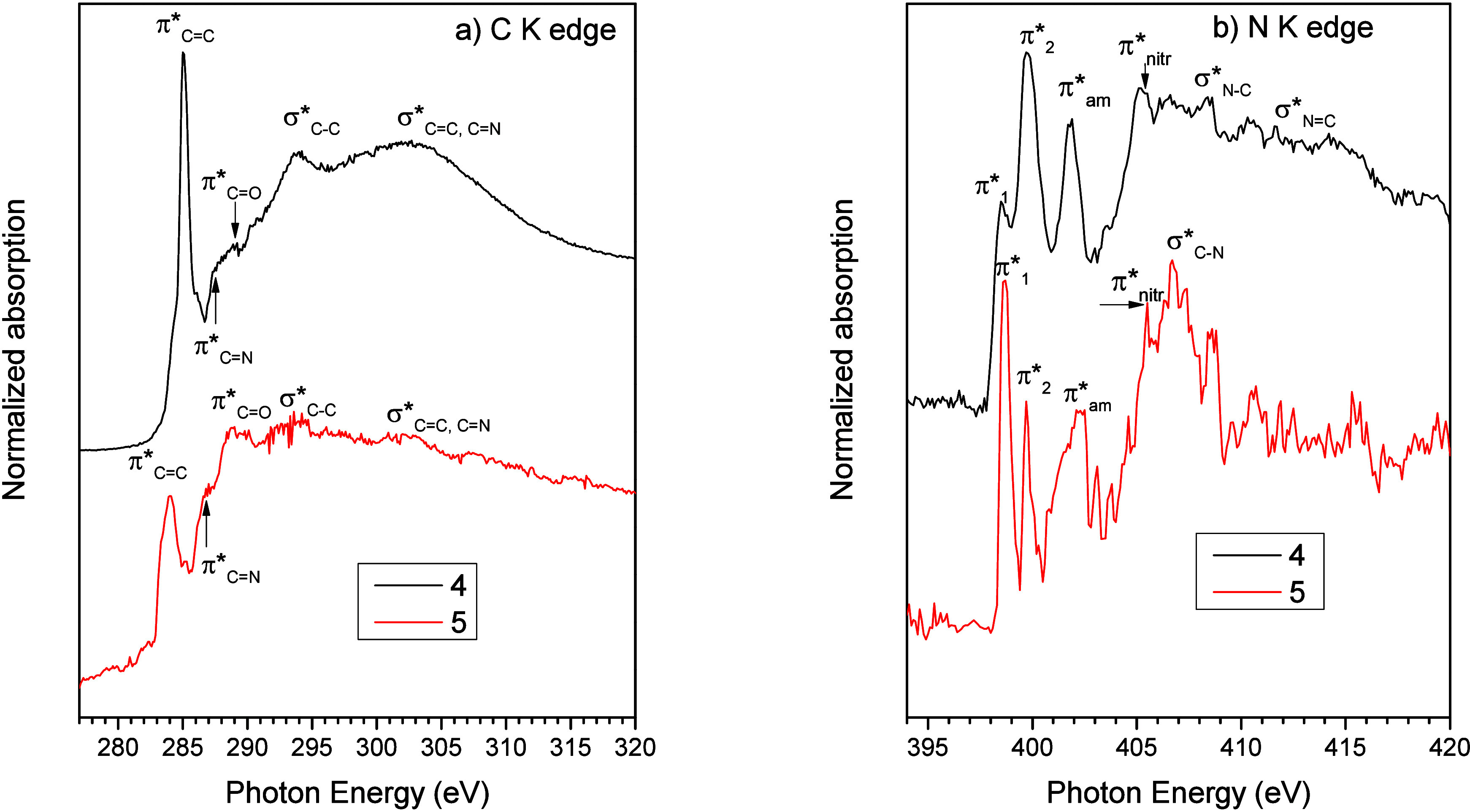
NEXAFS C K edge (a) and N K edge (b) NEXAFS spectra of complexes 4 (black lines) and 5 (red lines).

The C K edge spectrum of complex 4 (Figure 4a, black line) is dominated by an intense peak at 285.0 eV labeled π*_C=C_ in the figure and related to C 1s→π* transitions arising from C=C bonds of the benzene rings of the triphenylphosphine ligand.^64^ A peak related to C 1s→π* transitions, located at about 284 eV, is present also in the spectrum of complex 5 (Figure 4a red line), related to C=C carbons in the pyrazole moiety of the L^2Ad^ ligand; this peak can be seen as a shoulder on the low photon energy side of the more intense π*_C=C_ resonance related to the benzene rings of triphenylphosphine. Other peaks detected below the edge in the spectra of both complexes are the π*_C=O_ resonance located at 288.8 eV, related to the C=O bond of the amide function in the L^2Ad^ ligand^117^ and the π*_C=N_ resonance, located at about 286.7 eV and appearing as a shoulder on the low photon energy side of the π*_C=C_ resonance, related to the pyrazole rings in the L^2Ad^ ligand, as already reported for similar systems.^47^ Above the edge, two broad σ* resonances can be assigned to singly bonded (σ*_C–C_, at about 294 eV) and doubly bonded (σ*_C=C_, _C=N_, at about 294 eV) carbons.^64^ The N K edge spectra of all 4 and 5, shown in Figure 4b, appear quite noisy due to the lower intensity of the signals arising from the nitrogen atoms, present in lower concentration compared to carbons in the investigated samples; nevertheless, at least in the region below the edge peaks appear well resolved. The spectra of the two complexes are very similar to each other and strongly resemble analogous spectra of copper complexes with pyrazole ligands.^47^ In the spectra of both complexes, the two peaks detected below the edge at 398.7 and 399.7 eV, labeled π*_1_ and π*_2_ respectively, can be assigned to N 1s→π* transitions arising from nitrogen atoms of the pyrazole rings. A third N 1s→π* transition, located at about 401.8–402.0 eV and labeled π*_am_, can be assigned to the amide functions of the L^2Ad^ ligand. Near the edge, the peak located at 405.3–405 eV can be assigned (π*_nitr_) is related to the nitrate counterion of the investigated complexes.^118^ Above the edge, two broad σ* resonances arising from singly bonded (σ*_C–N_, 407 eV) and doubly bonded (σ*_C=N_, 413 eV) nitrogens are detected;^64^ the second resonance cannot be clearly detected in the spectrum of complex 5, due to low signal-to-noise ratio.

### 3.5 Average Local Geometry around Ag Ions: XAS Studies

X-ray absorption spectroscopy (XAS) was employed at the Ag K edge to describe the average valence state, coordination chemistry and local atomic environment around the Ag.^119^ This was achieved by analysis of the near edge region (XANES) as well as analysis for the extended (EXAFS) region. Quantitative EXAFS data analysis required the preparation of theoretical models representing the expected local structure of the complexes around the absorber atoms. Geometry optimization methods paired with Density Functional Theory (DFT) were used to generate the atomistic structures following an already established approach from previous studies^50,120^ (described in the following paragraph). Amplitude and phase functions required for EXAFS calculations were derived from these structural models and were then used to fit the experimental data. The most relevant scattering paths were identified and grouped in coordination shells with similar distance and amplitude (related to the atomic number Z) to reduce the amount of variables in the fit.

#### 3.5.1 Structural Models for XAS Data Analysis: Density Functional Theory (DFT) Calculations

Realistic atomic models of complexes 2, 4, and 5 were obtained by Quasi Newton optimization method paired with DFT. A reasonable starting geometry of the local environment of the Ag atom in the cation of these complexes was prepared using the 3D open-source software Avogadro.^121^ DFT calculations were achieved using the open-source software ORCA 5.0.1 (using Becke ‘88 exchange and Perdew ‘86 correlation integrals within the energy functional).^122,123^ Karlsruhe orbital basis sets were used, specifically def2-SVP (Valence Double Zeta) and def2-TZVP (Valence Triple Zeta), respectively for lighter atoms (H, C, N, O, P) and for Ag atoms. The structures were relaxed to an absolute minimum of energy and the obtained structures are shown in Figure 5. The atomic clusters were then used to calculate (using FEFF8.4 program)^67^ the theoretical amplitude and scattering functions required to build the theoretical model EXAFS functions χ_i_ (single scattering, SS, or multiple scattering, MS) and identify the main contributions to be used in the analysis.

**Figure 5:**
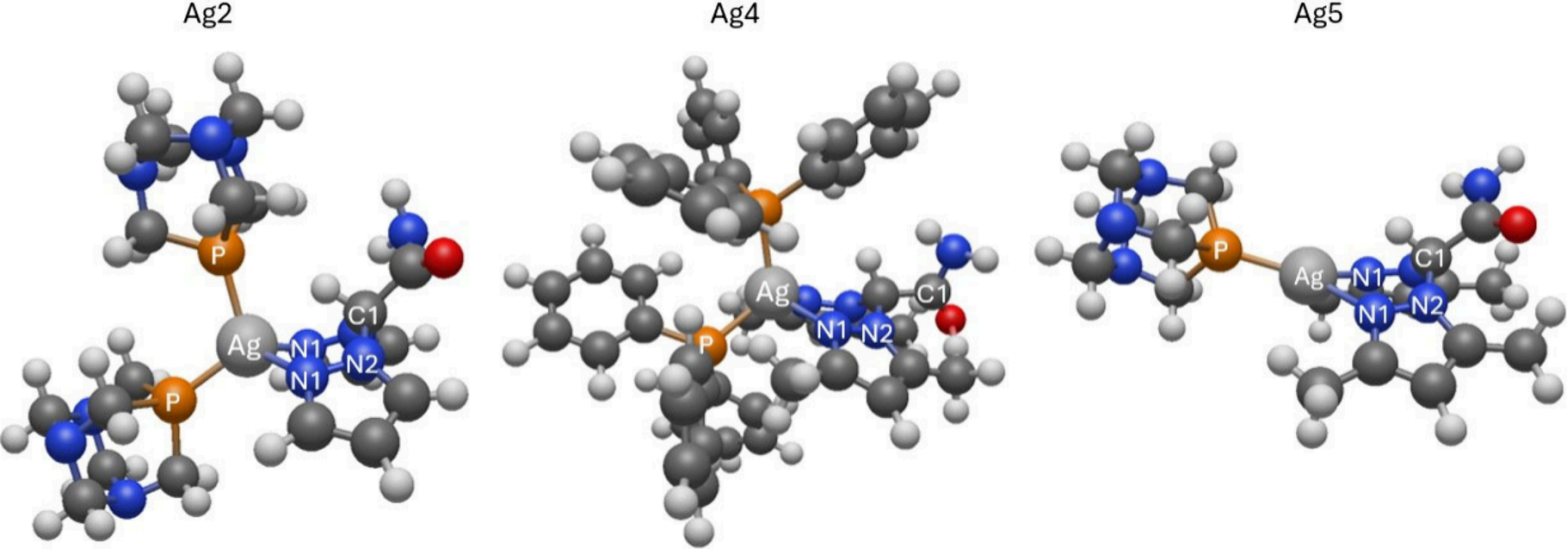
Structures of Ag coordination compounds 2, 4, and 5 obtained via QuasiNewton-DTF optimization. The absorber as well as the neighboring atoms involved in the most relevant scattering paths for EXAFS analysis are highlighted in the image. Numbering of the neighbors follows the increase in radial distance from the absorber.

#### 3.5.2 XAS Data Analysis

The XANES region of the spectra for complexes 2, 4, and 5 is reported in Figure 6a alongside the metallic reference material (Ag foil) as well as some spectra from Ag­(I) reference compounds. Analysis of the Ag K edge shows a slight change of the edge shape in the complexes with a reduced intensity in the pre-edge region and a shift of the edge (maximum of the first derivative) by 6 eV (complex 2), 2.6 eV (complex 4), 4.3 eV (complex 5) respect to the Ag-metal in agreement with the Ag^+^. Similar changes are evident in the XANES spectra of the reference compounds (from SSHADE).^124,125^ Additionally, the lack of any pre-edge peak, which is usually associated with transitions to partially empty d states, is another indication of the oxidation state in these systems since an Ag­(I) complex features a complete valence (4d^10^) shell and no transition from core levels can occur.

**Figure 6:**
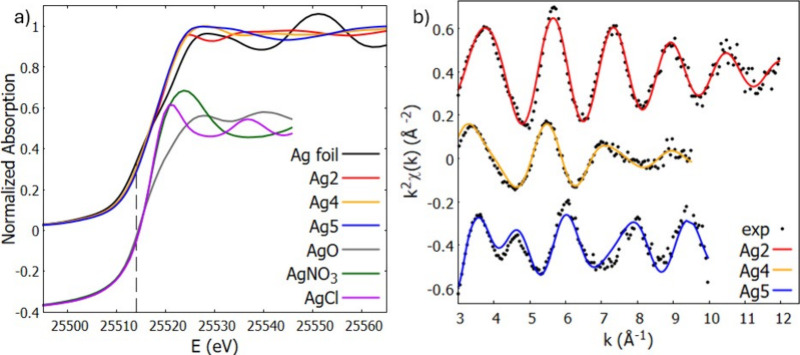
a) Ag K-edge XANES region for complexes 2, 4, and 5. Comparison between normalized data and AgO, AgNO_3_ and AgCl reference material (vertically shifted for clarity). b) Comparison of experimental EXAFS data and fit results in K space.

The spectra of complexes 2, 4, and 5 show a relatively smooth region above the edge, with damped structural features, likely due to larger structural disorder of the complexes with respect to bulk reference materials. The evident differences among the spectra of the different complexes in the region above 25525 eV point out differences in the local coordination geometries.

Quantitative EXAFS data analysis at the Ag K edge was carried out on complexes 2, 4, and 5 by selecting the photoelectron scattering amplitudes and phase functions from the most relevant single scattering contributions from the optimized theoretical models. k^2^-weighted experimental data k^2^χ_exp_(k) and best fit results are shown in Figure 6b. The analysis of the extended region of the spectra for the silver complexes was limited to the first two coordination shells due to the low signal-to-noise ratio and quite limited K range (12 Å^–1^ for complex 2 and 10 Å^–1^ for complexes 4 and 5). Up to 4 single scattering (SS) contributions were found relevant to fit the Ag local structure in the complexes. The structural parameters are resumed in Table 4. For all the samples, the Ag local coordination presents a similar bidentate ligand, a bis­(pyrazol-1-yl)­acetate for complex 2 and bis­(3,5-dimethyl-pyrazol-1-yl)­acetate for complexes 4 and 5, as well as a phosphine ligand being 1,3,5-triaza-7-phosphaadamantane for complexes 2 and 5, triphenylphosphine for complex 4, leading to the presence of common scattering paths in the EXAFS signal. In the first shell two single scattering contributions are found: the Ag–N1 shell with a multiplicity of 2 around 2.3 Å, and the Ag–P path around 2.4 Å with multiplicity 2 (complexes 2–4) or 1 (complex 5). Second shell contributions were found relevant in the analysis for complexes 4 and 5, they originate from single scattering paths with N2 and C1 neighbors. Since N and C have similar amplitude and phase functions in the EXAFS model and they are at similar distances, the two paths were grouped in a single contribution with multiplicity 3 (two N2 and one C1) and an average distance of 3.2 Å.

**Table 4:** Best Fit Results for Complexes 2, 4, and 5^a^

	Shell 1				Shell 2				Shell 3			
	Path	M	R [Å]	σ^2^ × 10^2^[Å^2^]	Path	M	R [Å]	σ^2^ × 10^2^[Å^2^]	Path	M	R [Å]	σ^2^ × 10^2^[Å^2^]
2	Ag–N1	2	2.37(1)	0.73(4)	Ag–P	2	2.43(3)	0.65(4)				
4	Ag–N1	2	2.28(1)	1.1(1)	Ag–P	2	2.40(4)	0.67(5)	Ag–N2+C1	3	3.29(2)	1.7(1)
5	Ag–N1	2	2.34(5)	0.65(8)	Ag–P	1	2.36(3)	0.25(4)	Ag–N2+C1	3	3.2(1)	1.6(3)

a S_0_^2^ was fixed to 0.9 for each sample, while the energy scale shift ΔE_0_ was fixed to 2 and 8 eV, respectively for complexes 2, 4, and 5. The scattering contributions are reported in terms of Path, multiplicity M, average distance R and mean square displacement σ^2^. Uncertainties on the last digit are reported in parentheses for refined parameters.

### 3.6 Stability Studies

The stability of the newly developed compound was assessed in 0.5% DMSO/saline solution by means of ^1^H-NMR analysis. Spectra were recorded over a range of time resembling biological studies (72h). All compounds proved to be suitably stable in physiological conditions, the changes observed in the ^1^H-NMR spectra being insignificant or only minimal (Figure S64).

### 3.7 Biological Studies

#### 3.7.1 Cytotoxicity Assays in 2D and 3D Cancer Cell Models

The new Ag­(I) compounds 1–7 together with free ligands and amantadine were evaluated for their cytotoxic effects by means of a 2D MTT assay against different human cancer cells, including colon (HCT-15), pancreatic (BxPC-3), lung (U-1285), breast (MCF-7), and cis-platin-sensitive ovarian (2008) carcinomas. Results expressed as IC_50_ values after 72 h of drug exposure are reported in Table 5. Cisplatin was employed as reference metallodrug. Table 5 also includes data obtained for human C13* ovarian cancer cells, a cisplatin-resistant variant of the 2008 line. The resistance factor (RF), defined as the ratio between IC_50_ values in resistant and sensitive cells, is indicated in parentheses.

**Table 5:** 2D Cytotoxic Activity^a^

IC_50_ (μM) ± SD							
	HCT-15	BxPC-3	U-1285	MCF-7	2008	C13* (R.F.)	CHO (S.I.)
Amantadine	>50	-	-	>50	-	-	-
L^Ad^	>50	-	-	>50	-	-	-
L^2Ad^	>50	-	-	>50	-	-	-
[Ag(L^Ad^)(PPh_3_)_2_]NO_3_ (1)	4.1 ± 1.0	2.5 ± 0.2	3.7 ± 1.1	12.2 ± 2.8	2.5 ± 0.1	6.3 ± 1.1 (2.5)	12.2 ± 2.2 (2.3)
[Ag(L^Ad^)(PTA)_2_]NO_3_ (2)	>50	22.0 ± 5.3	>50	>50	>50	>50 (−)	>50
[Ag(L^2Ad^)(PPh_3_)]NO_3_ (3)	4.7 ± 0.5	3.1 ± 0.6	4.5 ± 0.2	18.7 ± 0.6	4.9 ± 0.5	6.9 ± 0.7 (1.4)	13.5 ± 1.4 (1.9)
[Ag(L^2Ad^)(PPh_3_)_2_]NO_3_ (4)	4.6 ± 0.5	2.1 ± 0.8	4.8 ± 0.5	17.2 ± 0.3	3.1 ± 1.1	6.6 ± 0.6 (2.1)	11.8 ± 2.5 (1.8)
[Ag(L^2Ad^)(PTA)]NO_3_ (5)	20.1 ± 1.7	13.8 ± 4.5	28.5 ± 0.1	42.9 ± 1.8	32.1 ± 4.5	36.3 ± 5.9 (1.1)	75.6 ± 4.4 (2.6)
[Ag(L^2OiPr^)(PPh_3_)]NO_3_ (6)	7.8 ± 1.1	5.6 ± 1.8	10.7 ± 1.2	23.5 ± 3.1	4.9 ± 0.5	8.1 ± 1.0 (1.6)	12.6 ± 2.4 (1.2)
[Ag(L^2OiPr^)(PTA)NO_3_ (7)	>50	34.3 ± 5.4	>50	>50	33.2 ± 2.2	>50 (−)	>50
Cisplatin	13.9 ± 1.6	13.9 ± 5.9	6.9 ± 1.1	8.8 ± 0.2	2.1 ± 1.3	28.6 ± 3.0 (13.0)	19.2 ± 3.1 (1.56)

a Cells ((3–8) × 10^3^ cell/well) were treated for 72 h with tested compounds. Cell viability was evaluated by means of the MTT test. The IC_50_ values were obtained by a four-parameter (4-PL) logistic model (*p* < 0.05). S.D. = standard deviation. Resistance factor (RF) = IC_50_ (resistant cells)/IC_50_ (wild-type cells) is indicated in parentheses. Selectivity index values (SI) = ratio between the average IC_50_ in normal cells and that in malignant cells.

The results clearly demonstrate that uncoordinated ligands and amantadine were virtually ineffective in reducing cell viability. Conversely, Ag­(I) complexes, albeit to varying degrees, exhibited a significant cytotoxic efficacy against tested cancer cell lines, with IC_50_ values comparable to those of cisplatin and in the low micromolar range. In particular, [Ag­(L^Ad^)­(PPh_3_)_2_]­NO_3_ (1), [Ag­(L^2Ad^)­(PPh_3_)]­NO_3_ (3), [Ag­(L^2Ad^)­(PPh_3_)_2_]­NO_3_ (4) and [Ag­(L^2OiPr^)­(PPh_3_)]­NO_3_ (6) complexes, bearing PPh_3_ as phosphine coligand, were the most active ones, displaying higher cytotoxicity than cisplatin. In contrast, complexes containing the PTA ligand were all less active than cisplatin, with IC_50_ values in some cases exceeding 50 μM.

For the PPh_3_-bearing complexes, the cytotoxic activity appears to be quite comparable, with no substantial differences detectable in relation to either the number of phosphine ligands or the dimethylation of the bis­(pyrazolyl)­acetate moiety. Of note, the comparison between [Ag­(L^2Ad^)­(PPh_3_)]­NO_3_ (3) and its unfunctionalized analog [Ag­(L^2OiPr^)­(PPh_3_)]­NO_3_ (6) demonstrates that the functionalization of the bis­(pyrazolyl)­acetate ligand with amantadine results in only a slight increase in cytotoxic activity.

Among the tested cancer cell lines, human BxPC-3 pancreatic cancer cells were the most sensitive to treatment with Ag­(I) complexes. In particular, [Ag­(L^Ad^)­(PPh_3_)_2_]­NO_3_ (1) and [Ag­(L^2Ad^)­(PPh_3_)_2_]­NO_3_ (4) were both approximately five times more efficient than cisplatin in reducing cancer cell viability.

Table 5 also shows that all silver­(I) complexes exhibited similar activity levels on both cisplatin-sensitive (2008) and -resistant (C13*) cell lines. The calculated RF values indicated a promising ability to overcome cisplatin resistance. Notably, the RF values for the Ag­(I) derivatives were approximately six times lower than that of cisplatin, highlighting their potential, especially considering that drug resistance remains a critical challenge in anticancer therapy. The results obtained with the 2008/C13* cell pair clearly suggest that complexes may act through a mechanism distinct from that of conventional Pt­(II)-based drugs.

To obtain an initial indication of potential toxicity toward healthy cells, the cytotoxic activity of the newly synthesized Ag­(I) complexes was also examined in noncancerous CHO cells (Table 5). IC_50_ values obtained in CHO cells were consistently higher than the corresponding average values measured in tumor cell lines only for derivatives 1 and 5. For these compounds, the calculated selectivity index values (SI, defined as the ratio between the average IC_50_ in normal cells and that in malignant cells) were higher than that of cisplatin and exceeded a value of 2. This finding indicates a moderate but meaningful preference of these selected complexes for cancer cells over noncancerous cells. In contrast, the remaining compounds did not display a clear cancer-selective cytotoxic profile, showing only marginally greater activity against tumor cells compared to noncancerous cells.

To gain insight into the contribution of the amantadine moiety within the ligand scaffold to the antiproliferative efficacy of the phosphane Ag­(I) complexes, a pretreatment experiment with free amantadine was carried out in human pancreatic BxPC-3 cancer cells, which had shown the highest sensitivity to the novel silver compounds. BxPC-3 cells were pre-exposed to amantadine for 3 h, followed by a 24-h treatment with the corresponding amantadine-functionalized Ag­(I) complexes 1–5. Cell viability was subsequently assessed by MTT assay. As shown in Figure 7, the differences in cell viability between pretreated and non-pretreated samples were minimal and not statistically significant. These findings appear to rule out the involvement of saturable intracellular targets or transport systems specifically interacting with free amantadine in the cytotoxic potency of the Ag­(I) complexes. Rather than acting as a pharmacophore per se, it is more reasonable to assume that amantadine plays a structural or physicochemical role within the metal complex, such as modulating lipophilicity and, consequently, cellular uptake.

**Figure 7:**
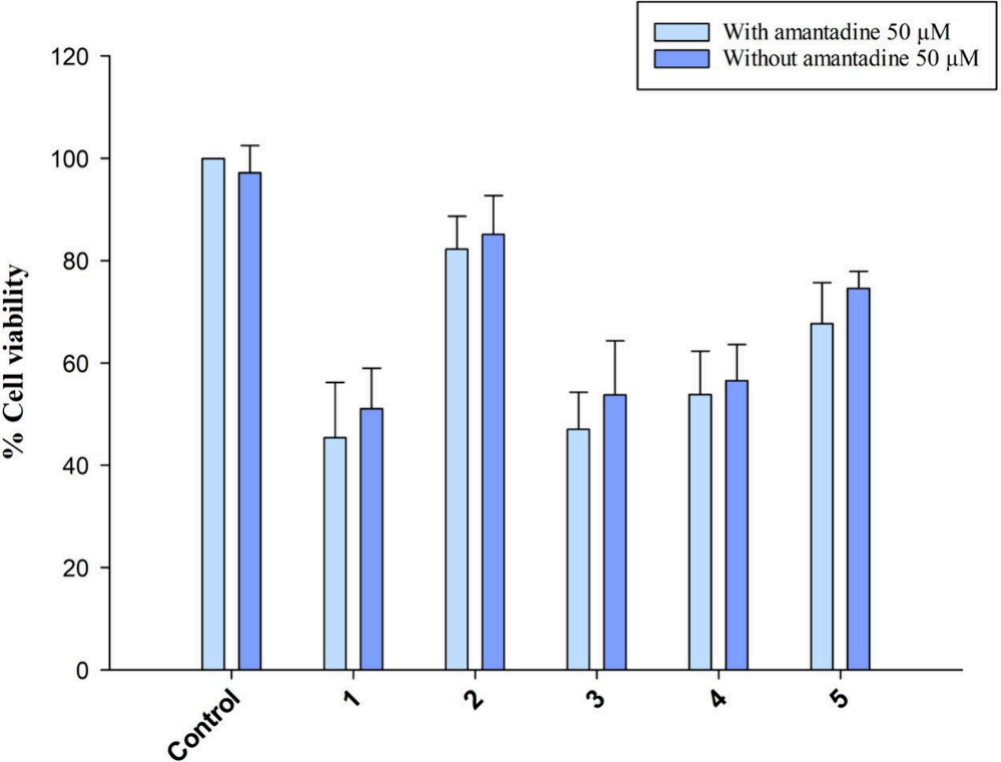
2D cytotoxic activity of the Ag­(I) complexes in presence or absence of pretreatment with amantadine. BxPC-3 cells (3 × 10^3^ cell/well) were treated for 3 h with amantadine (50 μM) followed by a 24-h treatment with tested complexes (10 μM). Cell viability was monitored by means of the MTT test. SD = standard deviation.

Given these encouraging findings, we further evaluated the activity of the Ag­(I) complexes in three-dimensional (3D) culture systems, using spheroids derived from human pancreatic BxPC-3 cancer cells, which had shown the highest sensitivity to the novel silver compounds.

3D culture models, providing a more faithful representation of the tumor microenvironment, offer a more physiologically relevant platform for assessing anticancer drug responses and have been shown to better predict in vivo therapeutic outcomes. BxPC-3 spheroids were treated with the tested compounds for 72 h, and cell viability was assessed using the acid phosphatase (APH) assay. The results, summarized in Table 6, showed IC_50_ values substantially higher than those obtained in 2D cultures, as expected. Indeed, cells grown in 3D typically exhibit reduced sensitivity to chemotherapeutic agents compared to 2D-cultured cells.^126,127^ Nonetheless, Ag­(I) complexes containing PPh_3_ as coligand were more efficacious than cisplatin in the 3D spheroid model, showing IC_50_ values roughly 1.5-fold lower than those of cisplatin. The most efficacious compound was [Ag­(L^2Ad^)­(PPh_3_)_2_]­NO_3_ (4), although this set of experiments confirmed that neither the number of phosphane ligands, nor the dimethylation or amantadine functionalization of the bis­(pyrazolyl)­acetate moiety resulted in marked differences in cytotoxic activity. The activity observed against the 3D cell culture model is particularly promising as it suggests that Ag­(I) complexes are capable of effectively penetrating the spheroid and reaching the central hypoxic core, thus exerting their cytotoxic effects. These results are consistent with the higher lipophilic character conferred by the triphenylphosphine ligand, which enhances the overall lipophilic character of the Ag­(I) complexes, as demonstrated by the experimental LogP values determined by the shake-flask method (Figure S65A). Indeed, an evident linear correlation between LogP and IC_50_ values can be observed (Figure S65B).

**Table 6:** Cytotoxicity of Ag­(I) Complexes and Cisplatin towards 3D Cell Culture Models^a^

IC_50_ (μM) ± SD	
	BxPC-3
[Ag(L^Ad^)(PPh_3_)_2_]NO_3_ (1)	43.4 ± 3.7
[Ag(L^Ad^)(PTA)_2_]NO_3_ (2)	>150
[Ag(L^2Ad^)(PPh_3_)]NO_3_ (3)	41.1 ± 3.2
[Ag(L^2Ad^)(PPh_3_)_2_]NO_3_ (4)	40.6 ± 3.3
[Ag(L^2Ad^)(PTA)]NO_3_ (5)	84.4 ± 3.1
[Ag(L^2OiPr^)(PPh_3_)]NO_3_ (6)	45.5 ± 3.6
[Ag(L^2OiPr^)(PTA)NO_3_ (7)	>150
Cisplatin	69.6 ± 8.2

a BxPC-3 cells (2.5 × 10^3^ × well) were treated for 72 h with tested compounds. Cell viability was monitored by means of the APH test. IC_50_ values were calculated from the dose-response curves by a 4-PL model (*p* < 0.05). S.D. = standard deviation.

#### 3.7.2 Cellular Uptake and Mechanistic Studies

To compare cytotoxic efficacy with intracellular silver content, cancer cell uptake studies were carried out in human BxPC-3 pancreatic cancer cells. Cells were treated for 24 h with 5 μM of tested complexes and silver content, quantified by graphite furnace atomic absorption spectrometry (GF-AAS) analysis and expressed as μg of metal per 10^6^ cells, is depicted in Figure 8A. The results clearly show that, unlike Ag­(I) complexes bearing the hydrophilic PTA phosphine ligand, all silver compounds containing the PPh_3_ ligand effectively accumulated within BxPC-3 cells. Notably, the highest intracellular silver content was observed in pancreatic cancer cells treated with [Ag­(L^2Ad^)­(PPh_3_)_2_]­NO_3_ (4), the complex containing the 3,5-dimethyl-bis­(pyrazolyl)­acetate ligand functionalized with amantadine and two triphenylphosphine coligands, presumably the most lipophilic compound in the series. When comparing the cellular uptake data with cytotoxicity results in BxPC-3 cells (Figure 8B), a general correlation emerges between the extent of cellular internalization and cytotoxic potency. This supports the notion that intracellular accumulation is a key determinant of the cytotoxic efficacy of these compounds. In particular, [Ag­(L^2Ad^)­(PPh_3_)]­NO_3_ (3) and [Ag­(L^2Ad^)­(PPh_3_)_2_]­NO_3_ (4), which were the most active complexes in terms of cytotoxicity, also showed the highest levels of cellular internalization, further suggesting that their superior biological performance is closely linked to their increased lipophilicity. These findings point to a strong dependence of biological activity on lipophilic properties, which may govern both membrane permeability and intracellular accumulation.

**Figure 8:**
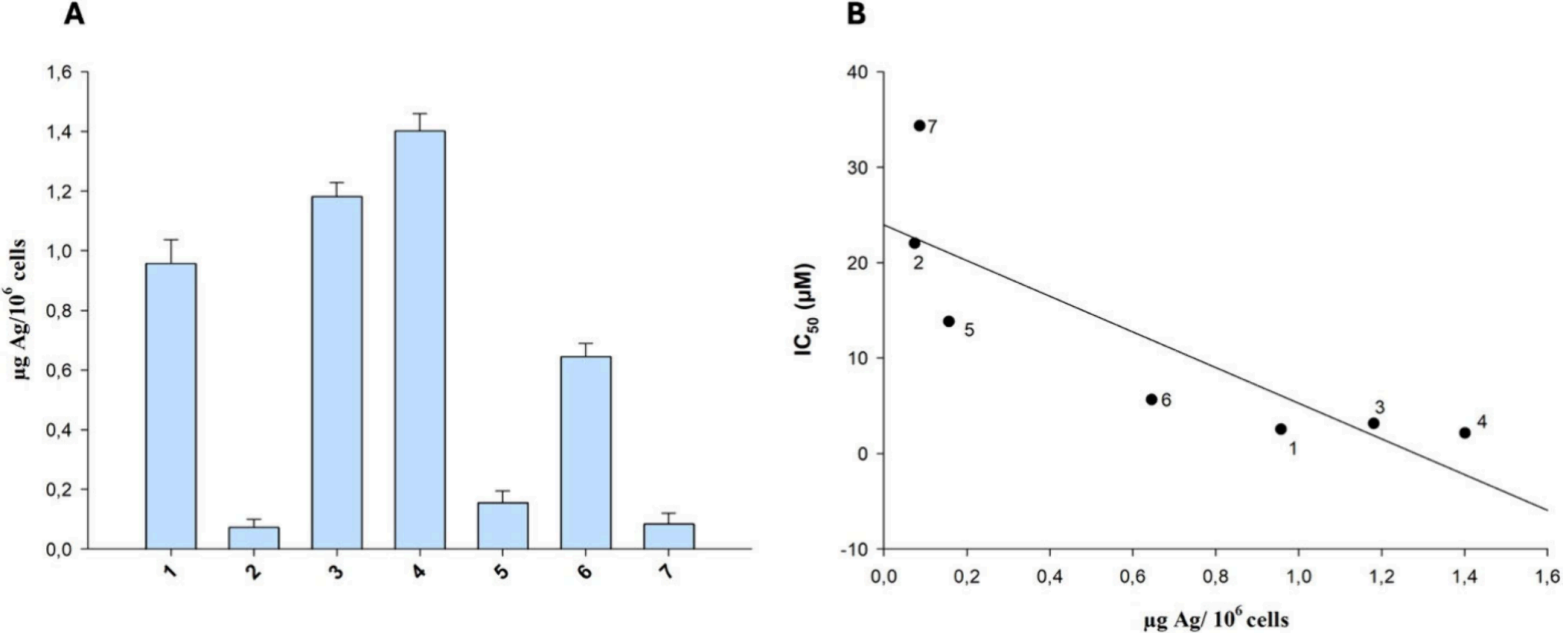
Cellular uptake studies. Intracellular content of silver (A) and correlation between silver content and cytotoxicity (B). BxPC-3 cells were incubated with 5 μM of tested complexes for 24 h, and cellular silver content was detected by GF-AAS analysis. Error bars indicate the standard deviation. ** < 0.01 compared with the control.

Ag­(I) complexes have already been assumed as inhibitors of redox-active selenoenzyme Thioredoxin Reductase. Due to its overexpression in many tumors and its involvement in cancer cell proliferation, survival, and resistance to therapy, TrxR is regarded as a promising target for the development of novel anticancer therapeutics.^128,129^ In this contest, we estimated the ability of the newly synthesized Ag­(I) compounds to inhibit TrxR activity, both in cell-free experiments and in human pancreatic BxPC-3 cells. Auranofin, a well- characterized metal-based TrxR inhibitor, was employed as a positive control. The inhibitory effects on TrxR activity were measured using standard procedures detailed in the Materials and Methods, and results (reported as the percentage of residual enzyme activity relative to the untreated control) are shown in Figure 9 (A and B). In cell-free assays, all Ag­(I) complexes were highly efficient in inhibiting cytosolic mammalian TrxR, although with varying degrees of potency, thus confirming literature data reported for other classes of Ag­(I) compounds. Among them, [Ag­(L^Ad^)­(PPh_3_)_2_]­NO_3_ (1) and [Ag­(L^2Ad^)­(PPh_3_)]­NO_3_ (3) exhibited the strongest TrxR inhibition, albeit their efficacy remained lower than that of the reference inhibitor auranofin (Figure 9A).

**Figure 9:**
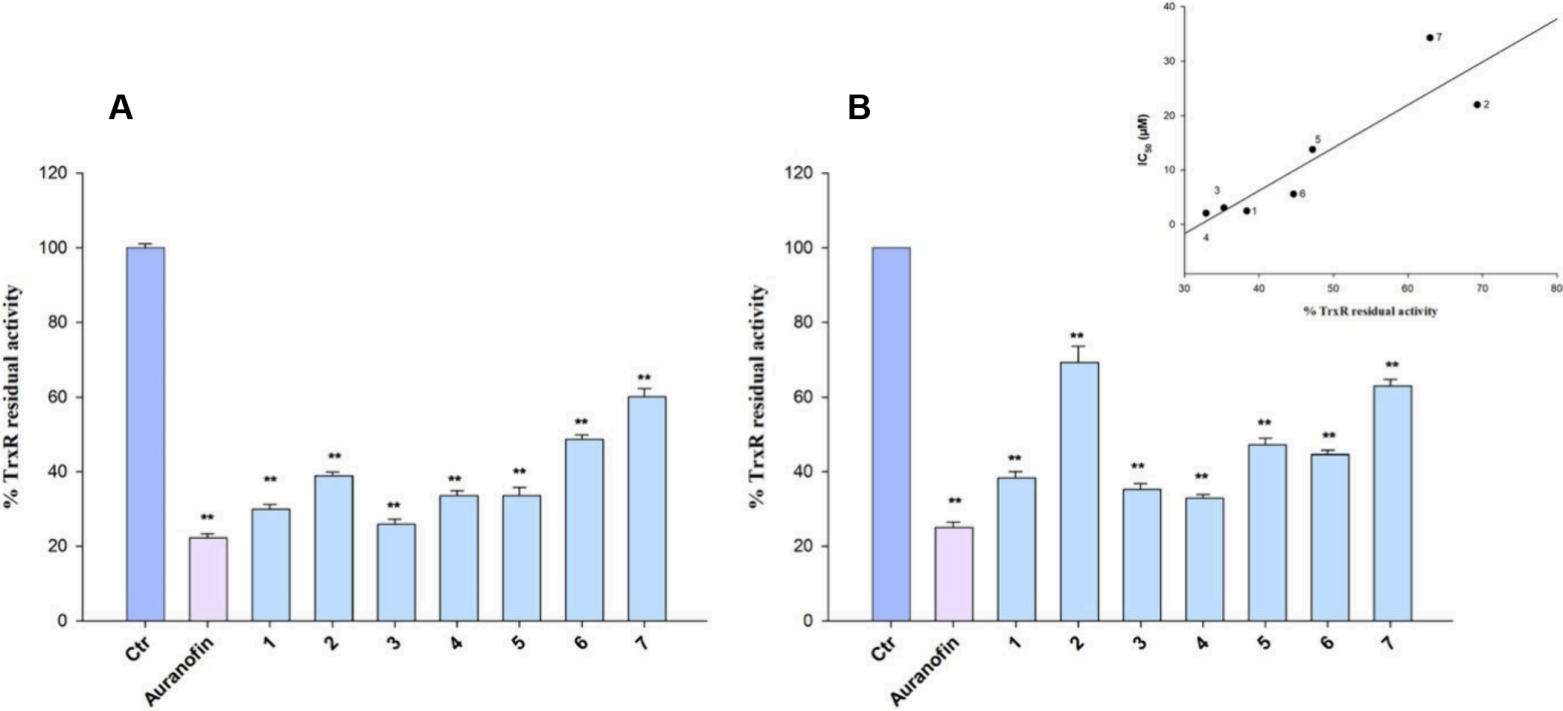
TrxR inhibition. (A) TrxR1 activity was assayed by measuring NADPH-dependent reduction of DTNB at 412 nm as described in Materials and Methods. Error bars indicate SD. (B) BxPC-3 cells were incubated for 24 h with Ag­(I) complexes (5 μM) or auranofin (1 μM). Subsequently, TrxR activity was tested in cell lysates by measuring NADPH-dependent reduction of DTNB at 412 nm. The inset shows the correlation between the IC_50_ values calculated for BxPC-3 cells and the percentage of residual TrxR activity. Error bars indicate SD * *p* < 0.05; ** *p* < 0.01.

In BxPC-3 cells, complexes 1, 3, 4 and 6, all bearing the PPh_3_ ligand, were effective in reducing the activity of the selenocysteine-containing redox enzyme TrxR by approximately 40%, clearly corroborating the hypothesis that they act as TrxR inhibitors in intact cancer cells (Figure 9B). Notably, TrxR inhibition was directly correlated with the cytotoxic profile of the compounds (see the inset in Figure 9B), thus underlying that TrxR can represent a key molecular target of these Ag­(I) complexes.

It is widely documented that the Trx system plays a crucial role in modulating cellular redox reactions thus strongly contributing to intracellular redox homeostasis. The inhibition of this redox-regulating pathway is known to disrupt the intracellular thiol–disulfide balance and to boost the accumulation of ROS, ultimately compromising cellular viability.^130^

The effects of the newly synthesized Ag­(I) complexes on cellular thiols content and ROS generation were investigated in BxPC-3-treated cells (Figure 10). Total free sulfhydryl groups were quantified using the DTNB assay after treatment of BxPC-3 cells with the silver complexes (5 μM), while auranofin (1 μM) served as a positive control.

**Figure 10:**
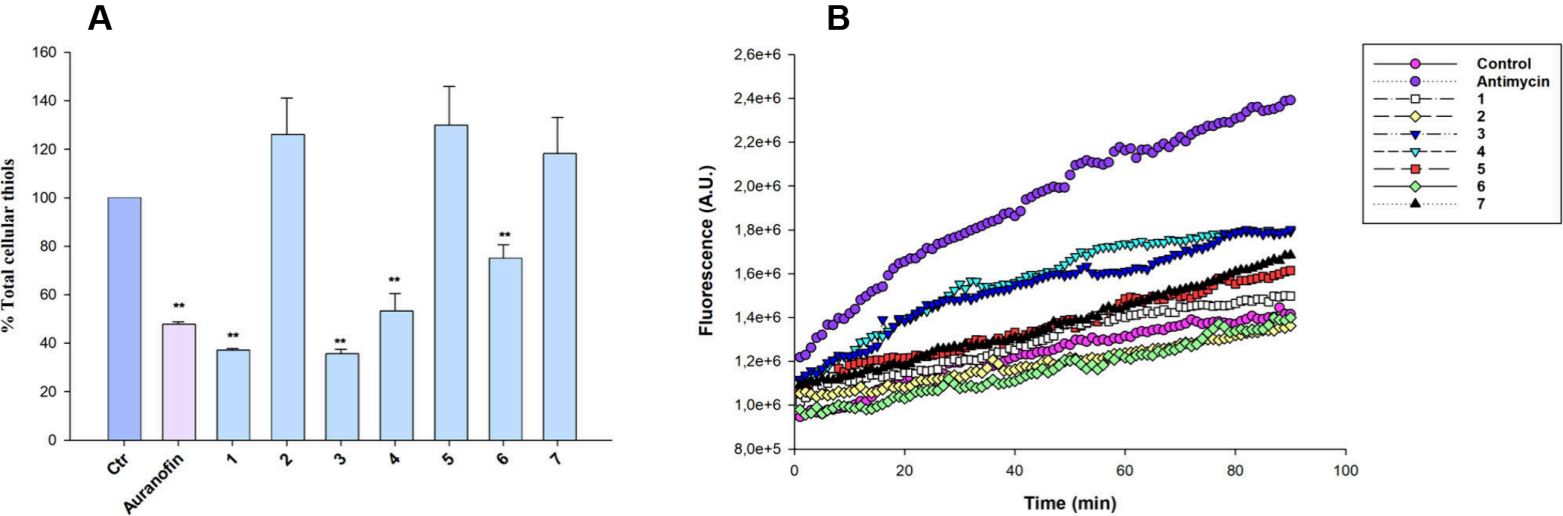
Effects on sulfhydryl content and ROS production. (A) Sulfhydryl content in BxPC-3-treated cancer cells incubated for 24 h with Ag­(I) complexes (5 μM) and auranofin (1 μM). The sulfhydryl group amount was determined by the DTNB assay. Error bars indicate SD * *p* < 0.05, ** *p* < 0.01. (B) Effect of silver­(I) compounds on hydrogen peroxide formation in BxPC-3 cells. Cells were preincubated in PBS/10 mM glucose medium for 20 min at 37 °C in the presence of 10 μM CM–DCFDA and then treated with Ag­(I) complexes (25 μM) or antimycin (3 μM).

As shown in Figure 10A, [Ag­(L^2Ad^)­(PPh_3_)]­NO_3_ (3) and [Ag­(L^2Ad^)­(PPh_3_)_2_]­NO_3_ (4), the most cytotoxic and potent TrxR inhibitors, were the most effective in depleting intracellular thiol groups. In addition, they were also the most effective in promoting ROS accumulation. Specifically, treatment of BxPC-3 cells with these complexes resulted in a marked, time- and dose-dependent increase in hydrogen peroxide levels (Figure 10B), albeit to a lesser extent than that caused by antimycin A, a well-known complex III inhibitor of the mitochondrial respiratory chain.

These findings further support the hypothesis that TrxR inhibition and thiol depletion are tightly linked to oxidative stress induction. Altogether, the data suggest that disruption of the Trx system represents a key pharmacodynamic mechanism driving the antiproliferative activity of this class of Ag­(I) complexes.

## 4 Conclusions

In this study, the chelating species bis­(pyrazol-1-yl)- and bis­(3,5-dimethyl-pyrazol-1-yl)-acetic acid were conjugated to the drug amantadine to obtain the ligands L^Ad^ and L^2Ad^, which were used for the synthesis of the new silver­(I) complexes 1–5. The hydrophilic 1,3,5-triaza-7-phosphaadamantane and the lipophilic triphenylphosphine, selected as coligands to stabilize silver in the +1 oxidation state, also impart distinct hydro-lipophilic balances to the resulting complexes. In addition, two unfunctionalized silver­(I) analogs [Ag­(L^2OiPr^)­(PPh_3_)]­NO_3_ (6) and [Ag­(L^2OiPr^)­(PTA)]­NO_3_ (7), were synthesized and studied for useful comparison. All complexes were fully characterized in the solid state and in solution. In solid state, XPS and NEXAFS measurements allowed to assess the molecular stability of L^2Ad^, L^Ad^ and phosphane ligands upon coordination with silver ions, as well as to unambiguously confirm the Ag­(I) oxidation state. The XRD investigations revealed that [Ag­(L^2Ad^)­(PPh_3_)]­NO_3_·0.5CH_3_CN (3a) is the first reported example of a silver­(I) tetra-coordinate complex containing a tridentate-N,N,O bis­(pyrazolyl) ligand. In the latter, hybrid HF-DFT calculations suggest that the effective participation of the neutral acetamide oxygen to the silver coordination sphere might be favored by crystal packing forces.

All synthesized Ag­(I) complexes and free ligands were assessed in order to evaluate their cytotoxic potential on various human cancer cell lines. Among them, complexes 1, 3, 4 and 6, all bearing the triphenylphosphine coligand, emerged as the most effective compounds. These achievements are in line with our previous findings on phosphane copper complexes bearing the same amantadine-functionalized bis­(pyrazolyl)­acetate ligand.^47^ Variations in the coordination environment, including the number of phosphane ligands and dimethylation of the bis­(pyrazolyl)­acetate moiety, did not result in significant differences in cytotoxicity, as well as the functionalization of the bis­(pyrazolyl)­acetate ligand with amantadine only led to a modest increase in cytotoxic activity. Accordingly, pretreatment experiments with free amantadine indicate that, rather than acting as a pharmacophore per se, amantadine only modulates complex lipophilicity and, consequently, cellular uptake.

Indeed, lipophilicity emerged as a critical determinant of biological activity for this class of Ag­(I) based compounds. Complexes 3 and 4, which were the most active in terms of cytotoxicity, also showed the highest levels of cellular internalization. Their enhanced lipophilicity, largely attributable to the triphenylphosphine moiety, appears to facilitate membrane permeability and intracellular accumulation. In line with these observations, the same complexes exhibited superior efficacy in the 3D spheroid model of BxPC-3 pancreatic cancer cells, where complexes 1, 3, 4, and 6 were about 1.5-fold more effective than cisplatin. Cytotoxicity assays performed on 2008/C13* cell pair suggested that the Ag­(I) complexes act via a mechanism of action distinct from platinum-based drugs. Enzymatic assays revealed potent inhibition of TrxR, both in cell-free systems and intact BxPC-3 cells, indicating this selenoenzyme as a primary molecular target. The changes observed in total cellular sulfhydryl content and ROS levels further support the involvement of the TrxR system disruption as a key mechanism underlying the antiproliferative effects of these Ag­(I) complexes, as also observed for other series of silver containing agents.^128,129^

In summary, this study highlights the critical role of physicochemical properties, particularly lipophilicity, and TrxR inhibition in shaping the cytotoxic properties of this class of phosphane Ag­(I) compounds, paving the way for the rational design of next-generation silver-complexes with improved cellular uptake and selective redox-modulating properties.

## Supplementary Material



## Notes

The data can be obtained free of charge from the Cambridge Crystallographic Data Centre via www.ccdc.cam.ac.uk/structures. The Supporting Information is available free of charge at https://pubs.acs.org/doi/10.1021/acsomega.5c10692.

Spectra and crystallographic data (PDF)
