# Supplementary material for: Synthesis, Structural Insights, and In Vitro Evaluation of Novel Silver Complexes Supported by Amantadine-Functionalized Bis(pyrazolyl)acetate Ligands as Anticancer Agents
Source: ACS Omega. 2026 May 6;11(19):27816–37. doi: 10.1021/acsomega.5c10692 (PMC13191531; doi:10.1021/acsomega.5c10692)
Supplement: Supplementary file 1 [file ao5c10692_si_001.pdf]

SUPPORTING INFORMATION

Synthesis, Structural Insights and In Vitro Evaluation of Novel Silver Complexes Supported by Amantadine-Functionalized Bis(pyrazolyl)acetate Ligands as Anticancer Agents

Maura Pellei,[a] Michele De Franco,[b] Carlo Santini,[a*] Miriam Caviglia,[a] Jo' Del Gobbo,[a] Luca Barigelli,[a] Fabio Del Bello,[c] Wilma Quaglia,[c] Chiara Battocchio,[d] Giovanna Iucci,[d] Iole Venditti,[d] Carlo Meneghini,[d] Simone Amatori,[d] Valentina Gandin,[b] Cristina Marzano,[b*] Alessandro Dolmella[b]

[a]School of Science and Technology, Chemistry Division, University of Camerino, via Madonna delle Carceri (ChIP), 62032 Camerino, Italy

[b]Department of Pharmaceutical and Pharmacological Sciences, University of Padova, via Marzolo 5, 35131 Padova, Italy

[c]School of Pharmacy, Medicinal Chemistry Unit, University of Camerino, via Madonna delle Carceri (ChIP), 62032 Camerino, Italy

[d]Department of Science, Roma Tre University, Via della Vasca Navale 79, 00146 Roma, Italy

* Correspondence: cristina.marzano@unipd.it (C.M.); carlo.santini@unicam.it (C.S.)

Table of Contents:

Figure S1. FT-IR spectrum of L^{Ad}.
Figure S2. ^{1}H-NMR spectrum of L^{Ad} in $CDCl_3$.
Figure S3. ^{1}H-NMR spectrum of L^{Ad} in CD_3CN.
Figure S4. $^{13}C\{^{1}H\}$-NMR spectrum of L^{Ad} in $CDCl_3$.
Figure S5. $^{13}C\{^{1}H\}$-NMR spectrum of L^{Ad} in CD_3CN.
Figure S6. HSQC spectrum of L^{Ad} in $CDCl_3$.
Figure S7. FT-IR spectrum of $[Ag(L^{Ad})(PPh_3)_2]NO_3$ (1).
Figure S8. ^{1}H-NMR spectrum of $[Ag(L^{Ad})(PPh_3)_2]NO_3$ (1) in $CDCl_3$.
Figure S9. ^{1}H-NMR spectrum of $[Ag(L^{Ad})(PPh_3)_2]NO_3$ (1) in CD_3CN.
Figure S10. $^{13}C\{^{1}H\}$-NMR spectrum of $[Ag(L^{Ad})(PPh_3)_2]NO_3$ (1) in CD_3CN.

Figure S11. $^{31}P\{^1H\}$-NMR spectrum of $[Ag(L^{Ad})(PPh_3)_2]NO_3$ (1) in CD_3CN at 293 K.
Figure S12. $^{31}P\{^1H\}$-NMR spectrum of $[Ag(L^{Ad})(PPh_3)_2]NO_3$ (1) in CD_3CN at 243 K.
Figure S13. HR-MS spectrum of $[Ag(L^{Ad})(PPh_3)_2]NO_3$ (1).
Figure S14. FT-IR spectrum of $[Ag(L^{Ad})(PTA)_2]NO_3$ (2).
Figure S15. ^1H-NMR spectrum of $[Ag(L^{Ad})(PTA)_2]NO_3$ (2) in DMSO-d_6.
Figure S16. $^{13}C\{^1H\}$-NMR spectrum of $[Ag(L^{Ad})(PTA)_2]NO_3$ (2) in DMSO-d_6.
Figure S17. $^{31}P\{^1H\}$-NMR spectrum of $[Ag(L^{Ad})(PTA)_2]NO_3$ (2) in CD_3CN at 293 K.
Figure S18. $^{31}P\{^1H\}$-NMR spectrum of $[Ag(L^{Ad})(PTA)_2]NO_3$ (2) in CD_3CN at 233 K.
Figure S19. HR-MS spectrum of $[Ag(L^{Ad})(PTA)_2]NO_3$ (2).
Figure S20. FT-IR spectrum of $[Ag(L^{2Ad})(PPh_3)]NO_3$ (3).
Figure S21. ^1H-NMR spectrum of $[Ag(L^{2Ad})(PPh_3)]NO_3$ (3) in CD_3CN.
Figure S22. ^1H-NMR spectrum of $[Ag(L^{2Ad})(PPh_3)]NO_3$ (3) in acetone-d_6.
Figure S23. $^{13}C\{^1H\}$-NMR spectrum of $[Ag(L^{2Ad})(PPh_3)]NO_3$ (3) in CD_3CN.
Figure S24. $^{31}P\{^1H\}$-NMR spectrum of $[Ag(L^{2Ad})(PPh_3)]NO_3$ (3) in CD_3CN at 293 K.
Figure S25. $^{31}P\{^1H\}$-NMR spectrum of $[Ag(L^{2Ad})(PPh_3)]NO_3$ (3) in CD_3OD at 223 K.
Figure S26. HR-MS spectrum of $[Ag(L^{2Ad})(PPh_3)]NO_3$ (3).
Figure S27. FT-IR spectrum of $[Ag(L^{2Ad})(PPh_3)_2]NO_3$ (4).
Figure S28. ^1H-NMR spectrum of $[Ag(L^{2Ad})(PPh_3)_2]NO_3$ (4) in $CDCl_3$.
Figure S29. ^1H-NMR spectrum of $[Ag(L^{2Ad})(PPh_3)_2]NO_3$ (4) in CD_3CN.
Figure S30. $^{13}C\{^1H\}$-NMR spectrum of $[Ag(L^{2Ad})(PPh_3)_2]NO_3$ (4) in CD_3CN.
Figure S31. $^{31}P\{^1H\}$-NMR spectrum of $[Ag(L^{2Ad})(PPh_3)_2]NO_3$ (4) in CD_3CN at 293 K.
Figure S32. $^{31}P\{^1H\}$-NMR spectrum of $[Ag(L^{2Ad})(PPh_3)_2]NO_3$ (4) in CD_3OD at 223 K.
Figure S33. HR-MS spectrum of $[Ag(L^{2Ad})(PPh_3)_2]NO_3$ (4).
Figure S34. FT-IR spectrum of $[Ag(L^{2Ad})(PTA)]NO_3$ (5).
Figure S35. ^1H-NMR spectrum of $[Ag(L^{2Ad})(PTA)]NO_3$ (5) in DMSO-d_6.
Figure S36. ^1H-NMR spectrum of $[Ag(L^{2Ad})(PTA)]NO_3$ (5) in CD_3CN.
Figure S37. ^1H-NMR spectrum of $[Ag(L^{2Ad})(PTA)]NO_3$ (5) in CD_3OD.
Figure S38. $^{13}C\{^1H\}$-NMR spectrum of $[Ag(L^{2Ad})(PTA)]NO_3$ (5) in CD_3CN.
Figure S39. $^{31}P\{^1H\}$-NMR spectrum of $[Ag(L^{2Ad})(PTA)]NO_3$ (5) in CD_3CN at 293 K.
Figure S40. $^{31}P\{^1H\}$-NMR spectrum of $[Ag(L^{2Ad})(PTA)]NO_3$ (5) in CD_3OD at 223 K.
Figure S41. HR-MS spectrum of $[Ag(L^{2Ad})(PTA)]NO_3$ (5).
Figure S42. FT-IR spectrum of $[Ag(L^{2OiPr})(PPh_3)]NO_3$ (6).
Figure S43. ^1H-NMR spectrum of $[Ag(L^{2OiPr})(PPh_3)]NO_3$ (6) in CD_3CN
Figure S44. ^{31}P-NMR spectrum of $[Ag(L^{2OiPr})(PPh_3)]NO_3$ (6) in CD_3CN at 293 K.

Figure S45. ^{31}P-NMR spectrum of $[Ag(L^{2OiPr})(PPh_3)]NO_3$ (6) in CD_3CN at 243 K.
Figure S46. HR-MS spectrum of $[Ag(L^{2OiPr})(PPh_3)]NO_3$ (6).
Figure S47. FT-IR spectrum of $[Ag(L^{2OiPr})(PTA)]NO_3$ (7).
Figure S48. ^{1}H-NMR spectrum of $[Ag(L^{2OiPr})(PTA)]NO_3$ (7) in DMSO-d_6.
Figure S49. ^{31}P-NMR spectrum of $[Ag(L^{2OiPr})(PTA)]NO_3$ (7) in CD_3OD at 293 K.
Figure S50. ^{31}P-NMR spectrum of $[Ag(L^{2OiPr})(PTA)]NO_3$ (7) in CD_3OD at 263 K.
Figure S51. FT-IR spectrum of crystals of $[Ag(L^{2Ad})(PPh_3)]NO_3$ (3a).
Figure S52. ^{1}H-NMR spectrum of crystals of $[Ag(L^{2Ad})(PPh_3)]NO_3$ (3a) in CD_3CN.
Figure S53. $^{13}C\{^{1}H\}$-NMR spectrum of crystals of $[Ag(L^{2Ad})(PPh_3)]NO_3$ (3a) in CD_3OD.
Figure S54. $^{31}P\{^{1}H\}$-NMR spectrum of crystals of $[Ag(L^{2Ad})(PPh_3)]NO_3$ (3a) in CD_3OD at 293 K.
Figure S55. HR-MS spectrum of crystals of $[Ag(L^{2Ad})(PPh_3)]NO_3$ (3a).
Figure S56. Packing diagram of intermolecular interactions for the ligand L^{Ad}.
Figure S57. Pairing between the $[Ag(L^{2Ad})(PPh_3)]^+$ cation and the nitrate anion in complex 3a.
Figure S58. Cation/anion paired pairs in complex 3a.
Figure S59. Paired cations in complex 3a.
Figure S60. One-dimensional chain of cation/anion paired pairs originated by H10B···O1 and H26····O2B contacts in complex 3a.
Figure S61. One-dimensional chain of cations originated by H10B···O1 and H9A····Ag1 contacts in complex 3a.
Figure S62. One-dimensional chain originated by H7····H31 contact in complex 3a.
Figure S63. Unit cell voids hosting the crystallization acetonitrile molecules in complex 3a.
Figure S64. Stability studies: ^{1}H-NMR spectra of compounds 1-7 in DMSO-d_6 (~0.4 mL), followed by dilution with D_2O to a final volume of 0.5 mL. Spectra were recorded at time points: a) t = 0; b) t = 24 h; c) t = 48 h; d) t = 72 h.
Figure S65. LogP values (A) and correlation with cytotoxicity in 3D system (B).
Table S1. List of crystallographic data for the ligand L^{Ad}.
Table S2. List of crystallographic data for the complex $[Ag(L^{2Ad})(PPh_3)]NO_3$ (3).
Table S3. Main nonbonding interactions (Å and degrees) for L^{Ad}.
Table S4. Main nonbonding interactions (Å and degrees) for the complex $[Ag(L^{2Ad})(PPh_3)]NO_3$ (3).

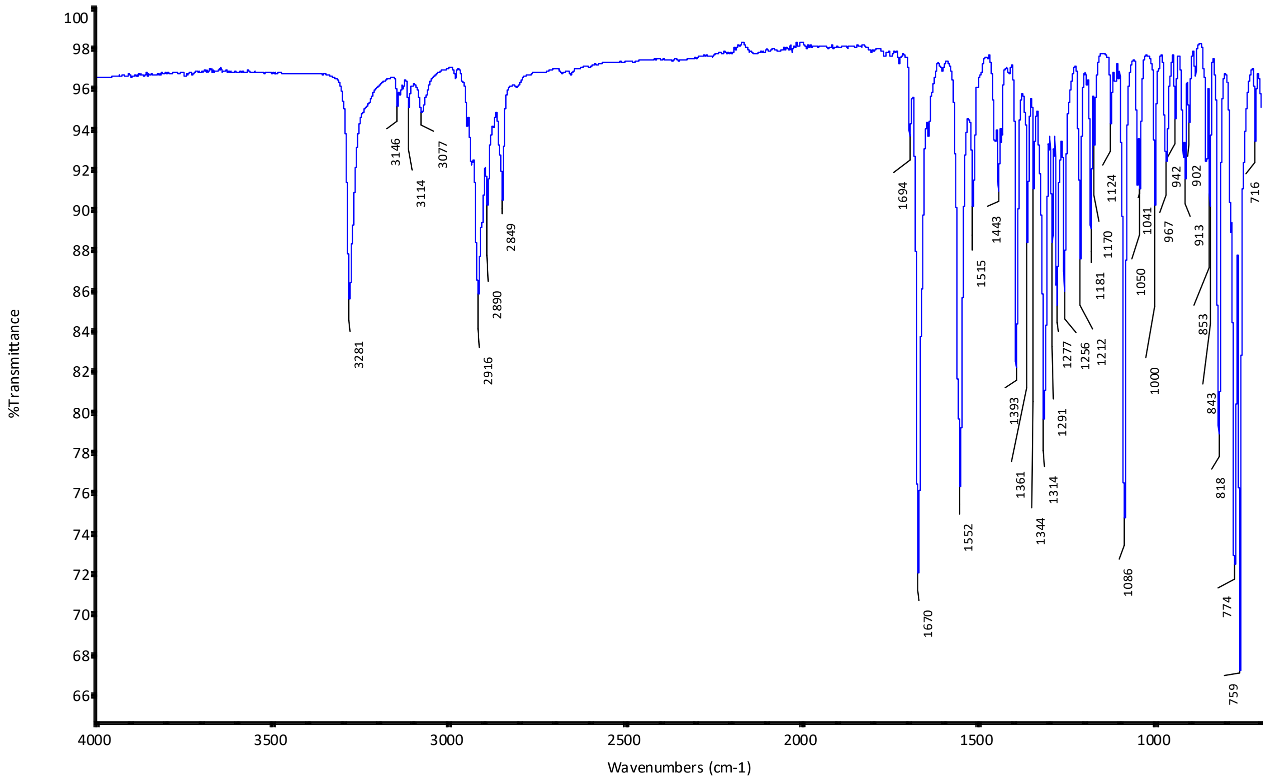

Figure S1. FT-IR spectrum of L^{Ad}.

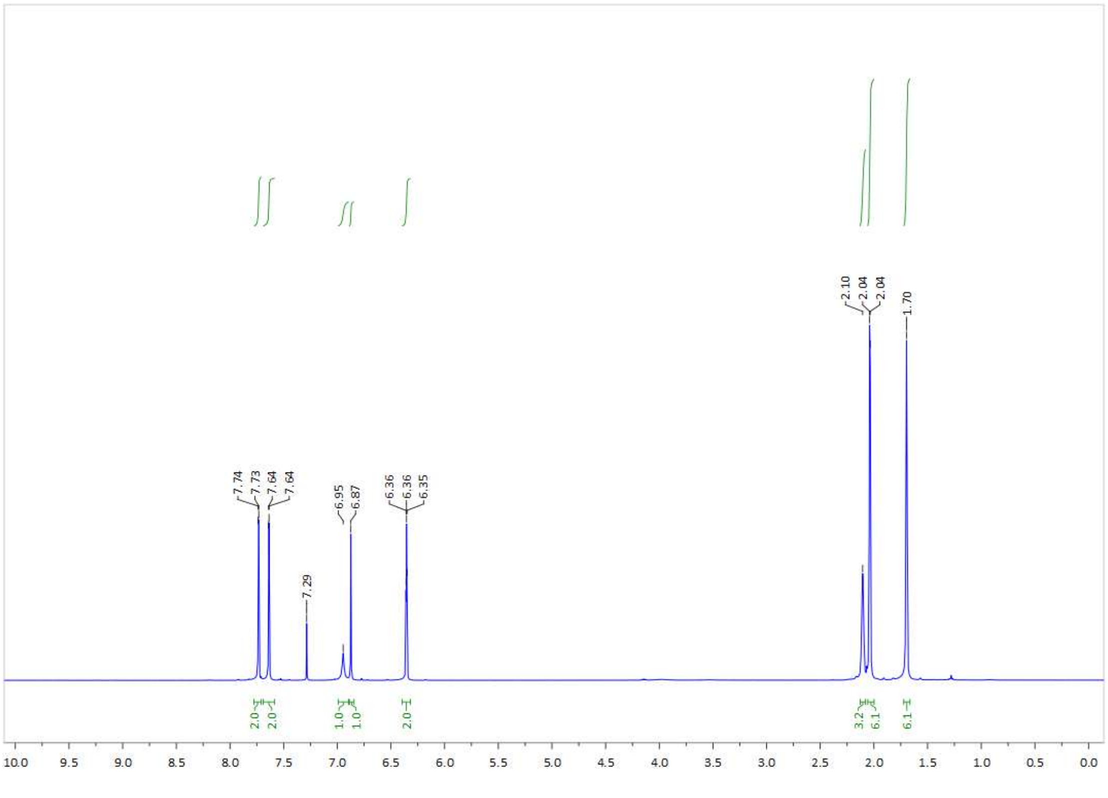

Figure S2. ^{1}H-NMR spectrum of L^{Ad} in $CDCl_3$.

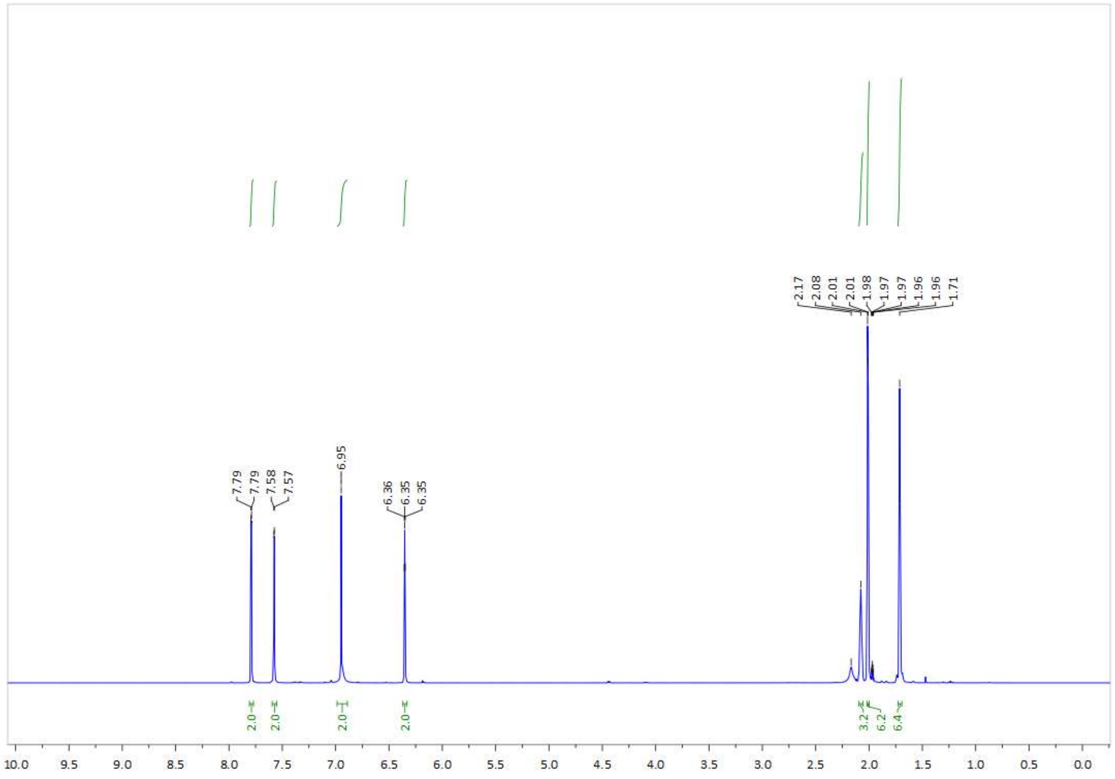

Figure S3. ^{1}H-NMR spectrum of L^{Ad} in CD_3CN.

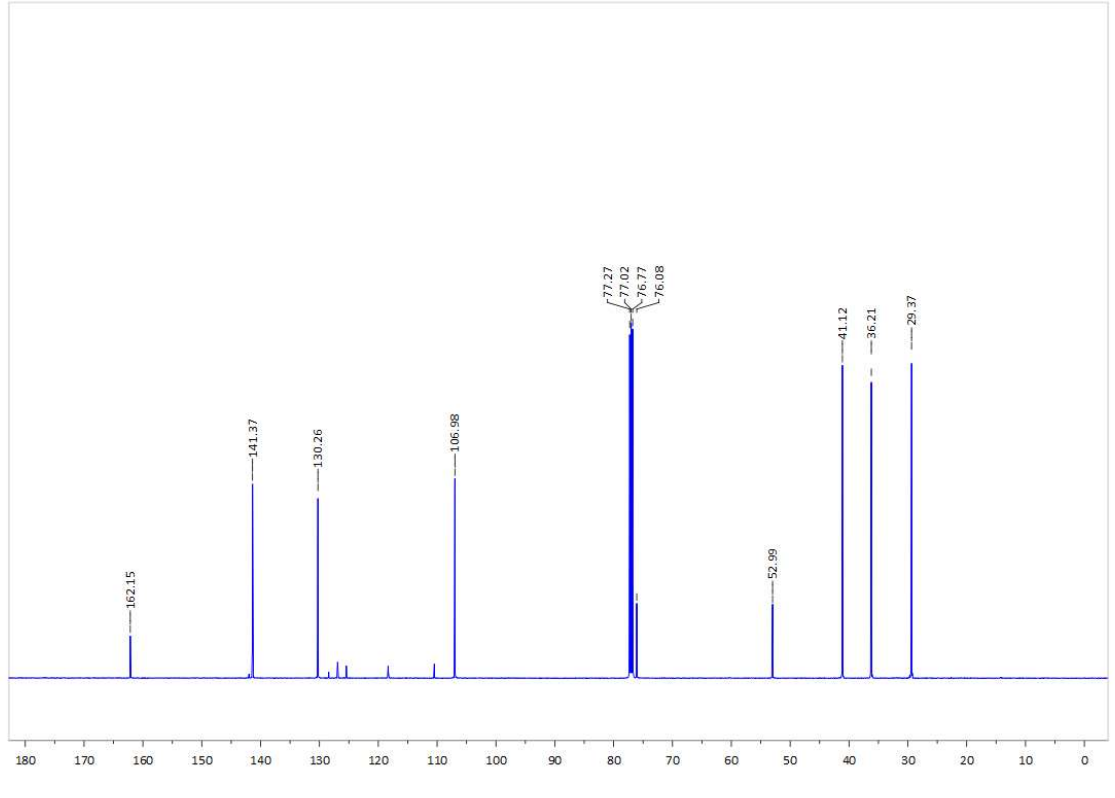

Figure S4. $^{13}C\{^{1}H\}$-NMR spectrum of L^{Ad} in $CDCl_3$.

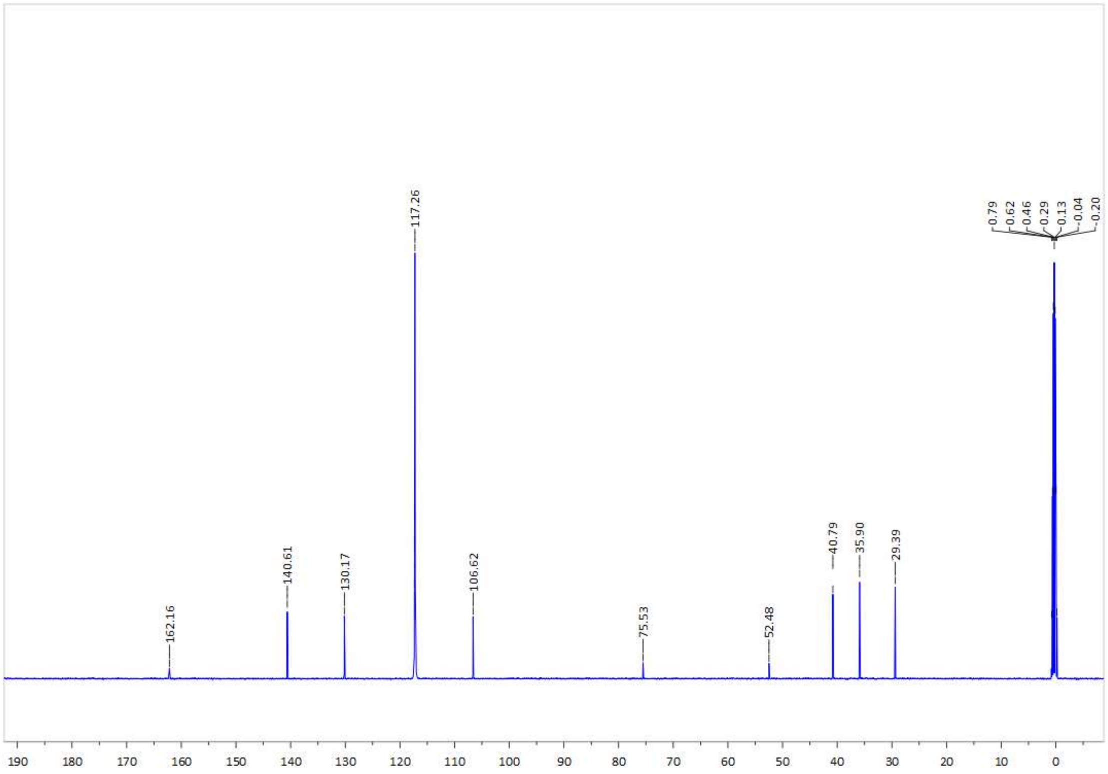

Figure S5. $^{13}C\{^1H\}$-NMR spectrum of L^{Ad} in CD_3CN.

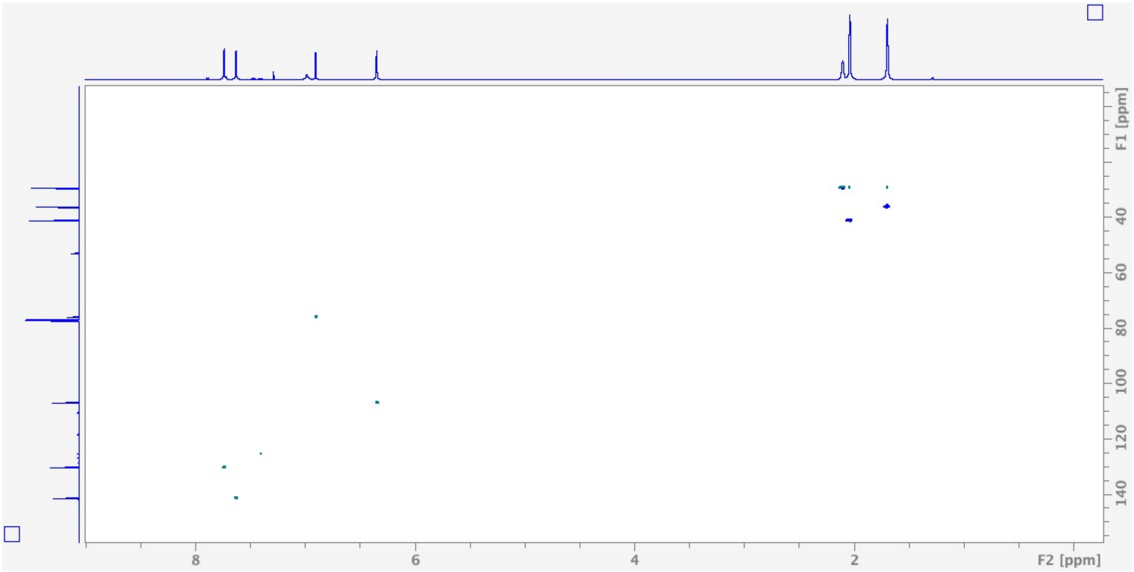

Figure S6. HSQC spectrum of L^{Ad} in $CDCl_3$.

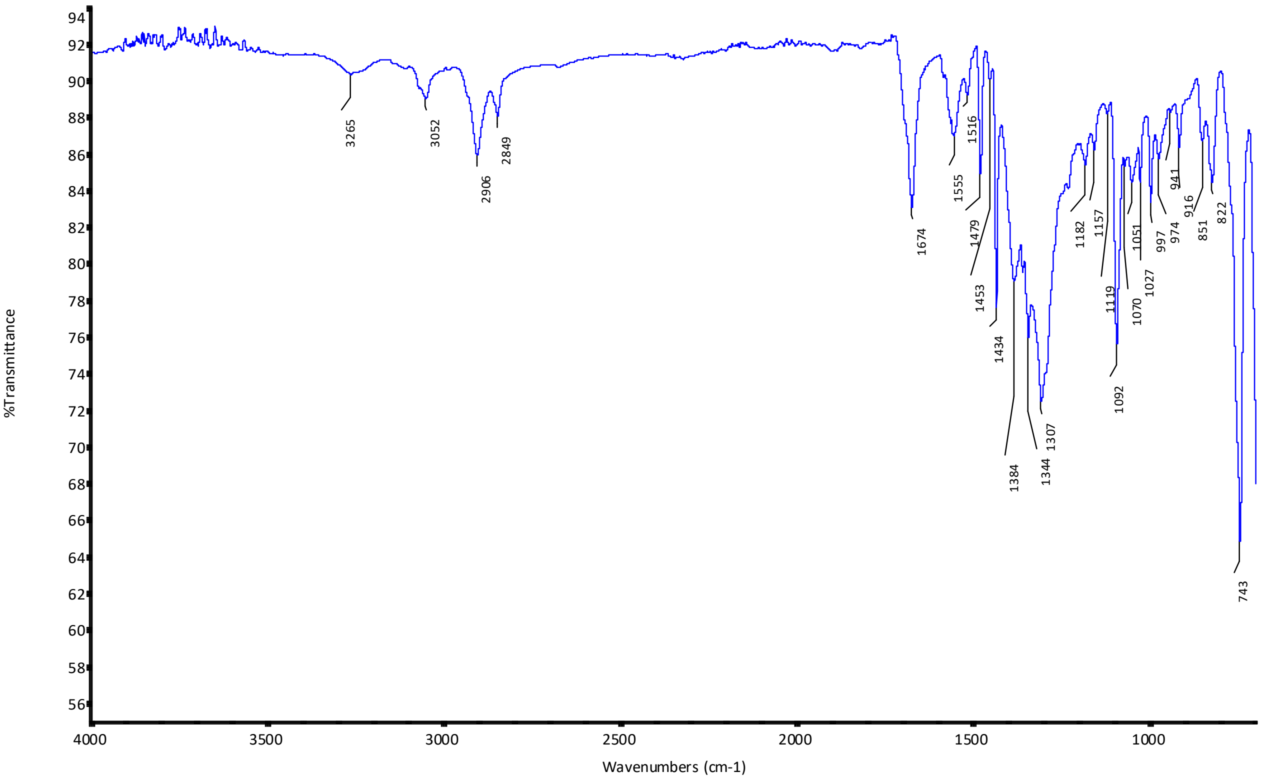

Figure S7. FT-IR spectrum of $[Ag(L^{Ad})(PPh_3)_2]NO_3$ (1).

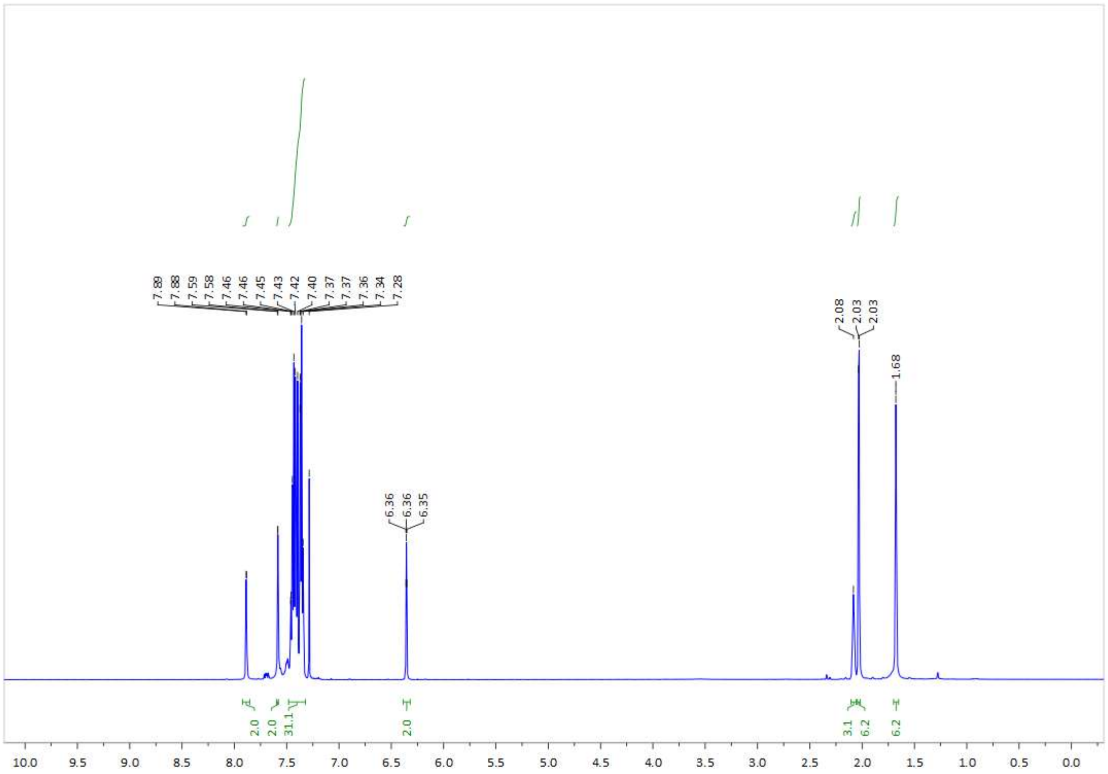

Figure S8. ^{1}H-NMR spectrum of $[Ag(L^{Ad})(PPh_3)_2]NO_3$ (1) in $CDCl_3$.

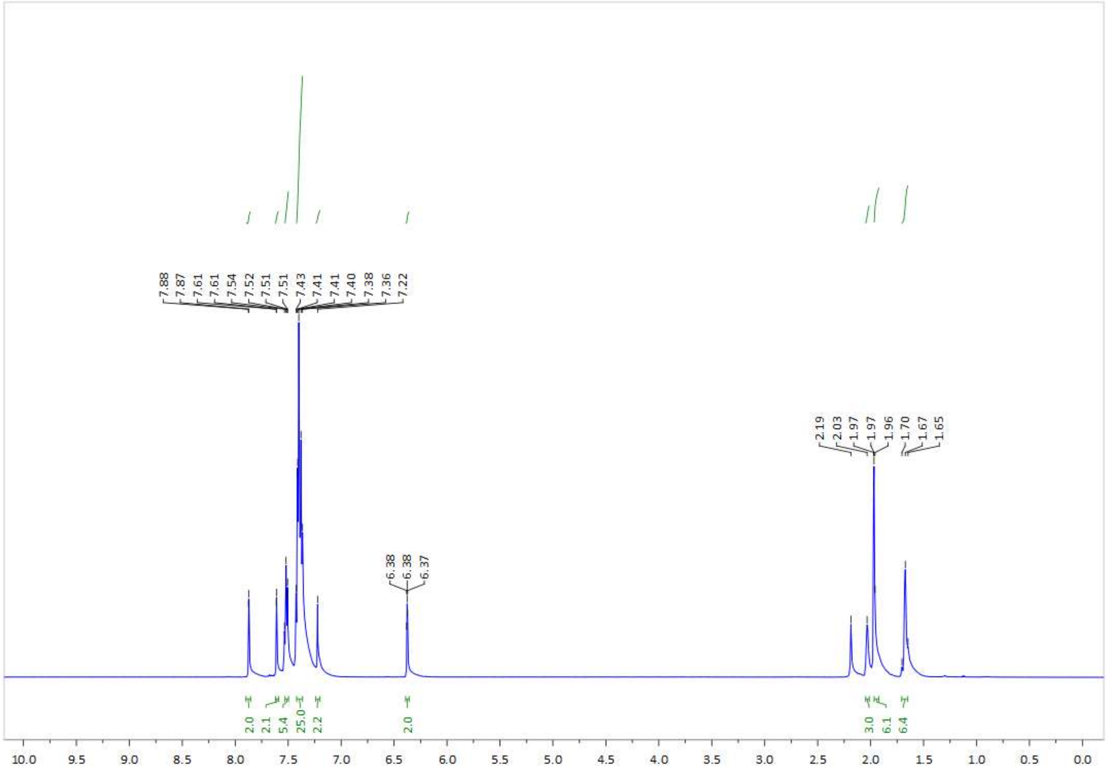

Figure S9. ^{1}H-NMR spectrum of $[Ag(L^{Ad})(PPh_3)_2]NO_3$ (1) in CD_3CN.

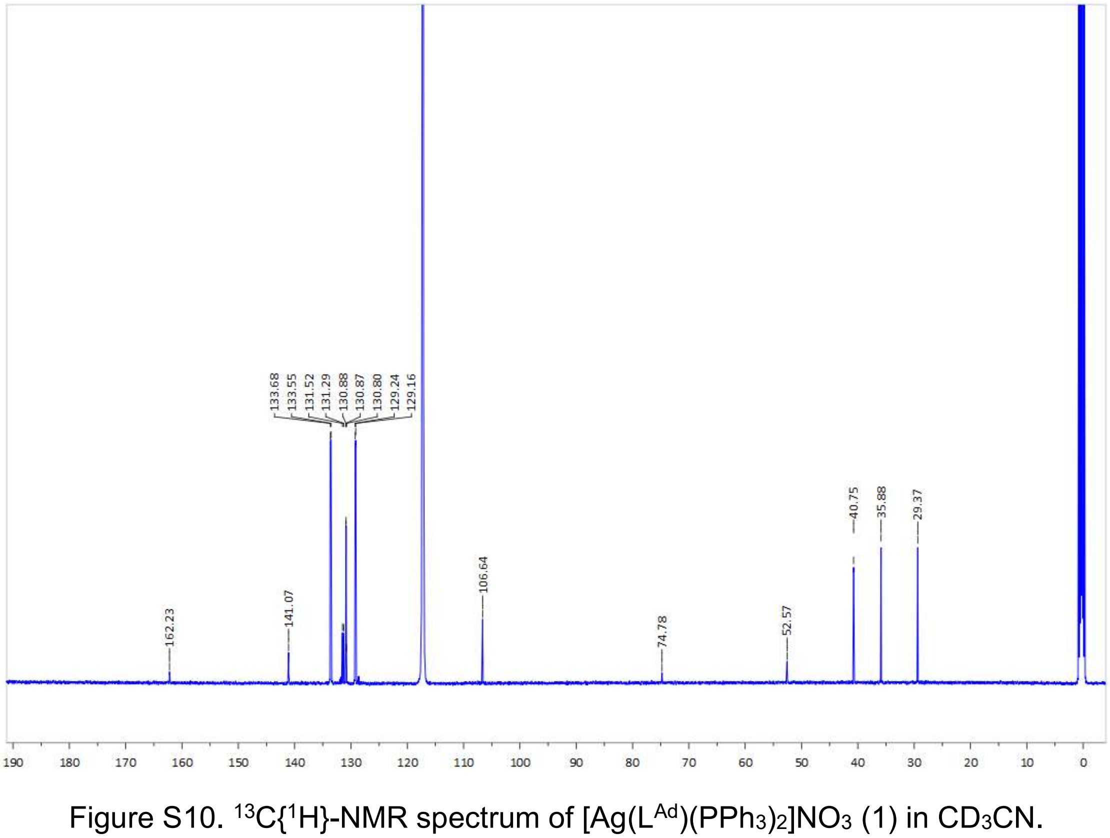

Figure S10. $^{13}C\{^{1}H\}$-NMR spectrum of $[Ag(L^{Ad})(PPh_3)_2]NO_3$ (1) in CD_3CN.

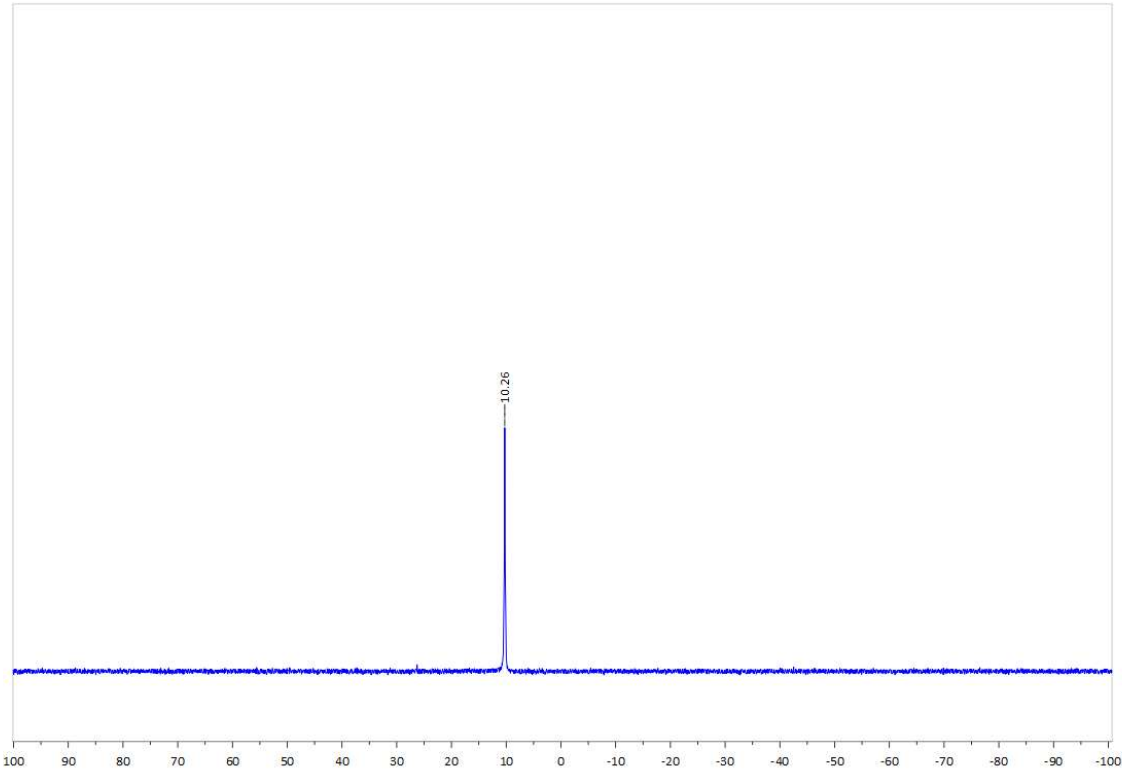

Figure S11. $^{31}P\{^{1}H\}$-NMR spectrum of $[Ag(L^{Ad})(PPh_3)_2]NO_3$ (1) in CD_3CN at 293 K.

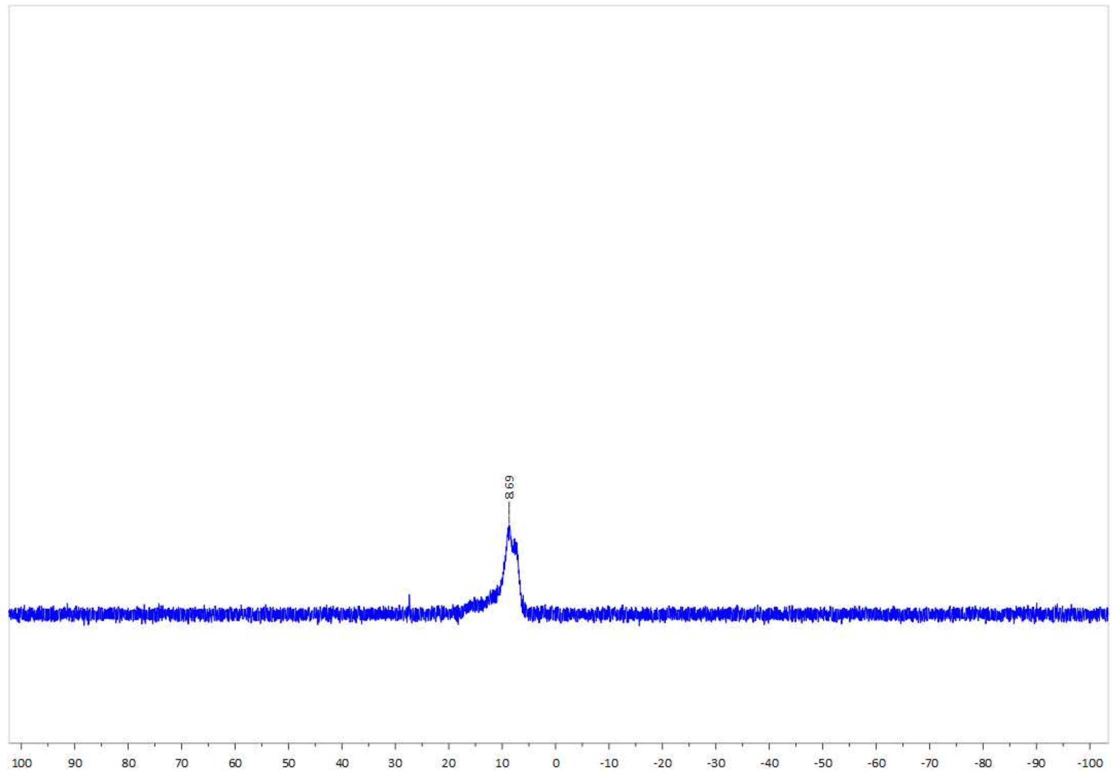

Figure S12. $^{31}P\{^{1}H\}$-NMR spectrum of $[Ag(L^{Ad})(PPh_3)_2]NO_3$ (1) in CD_3CN at 243 K.

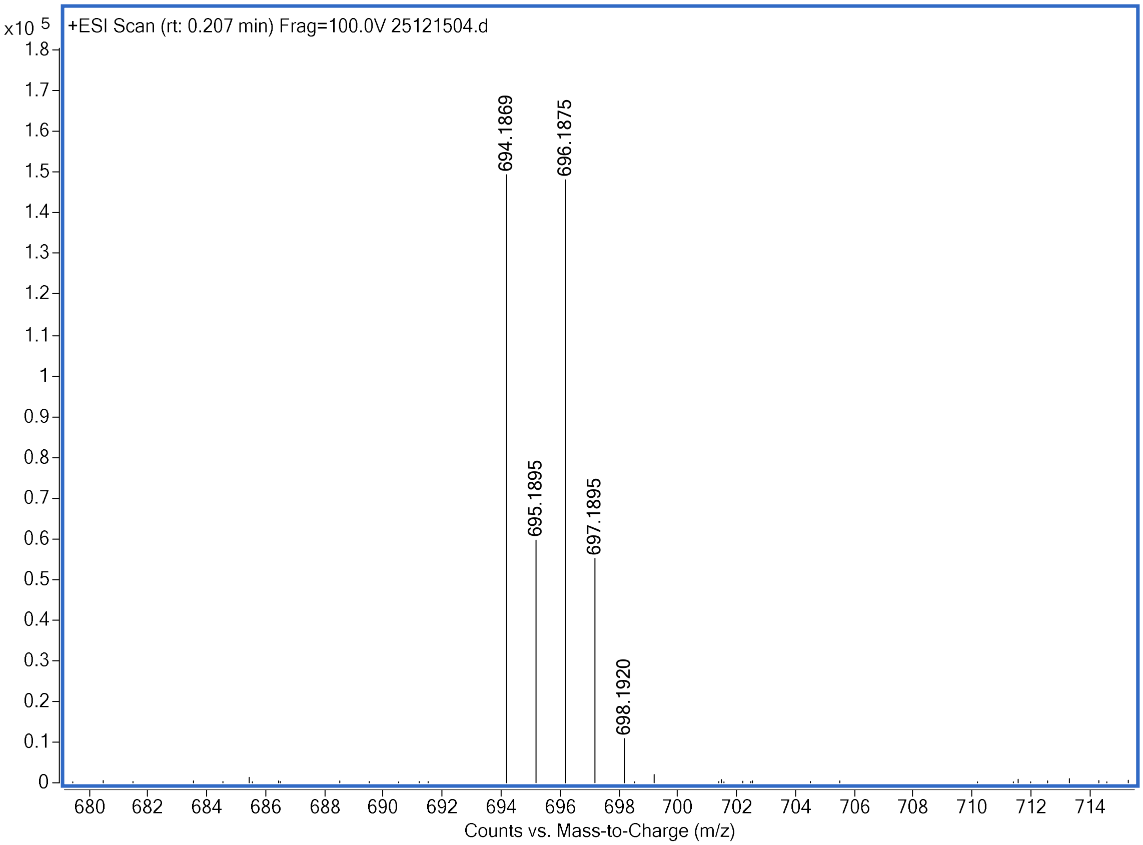

Figure S13. HR-MS spectrum of $[Ag(L^{Ad})(PPh_3)_2]NO_3$ (1), molecular monoisotopic mass ion peaks at m/z 694.1869 $[C_{36}H_{38}Ag^{106.9051}N_5OP]^+$ and 696.1875 $[C_{36}H_{38}Ag^{108.9048}N_5OP]^+$; HRMS-ESI(+) (Agilent 6545 – qTOF), $[M – PPh_3]^+$.

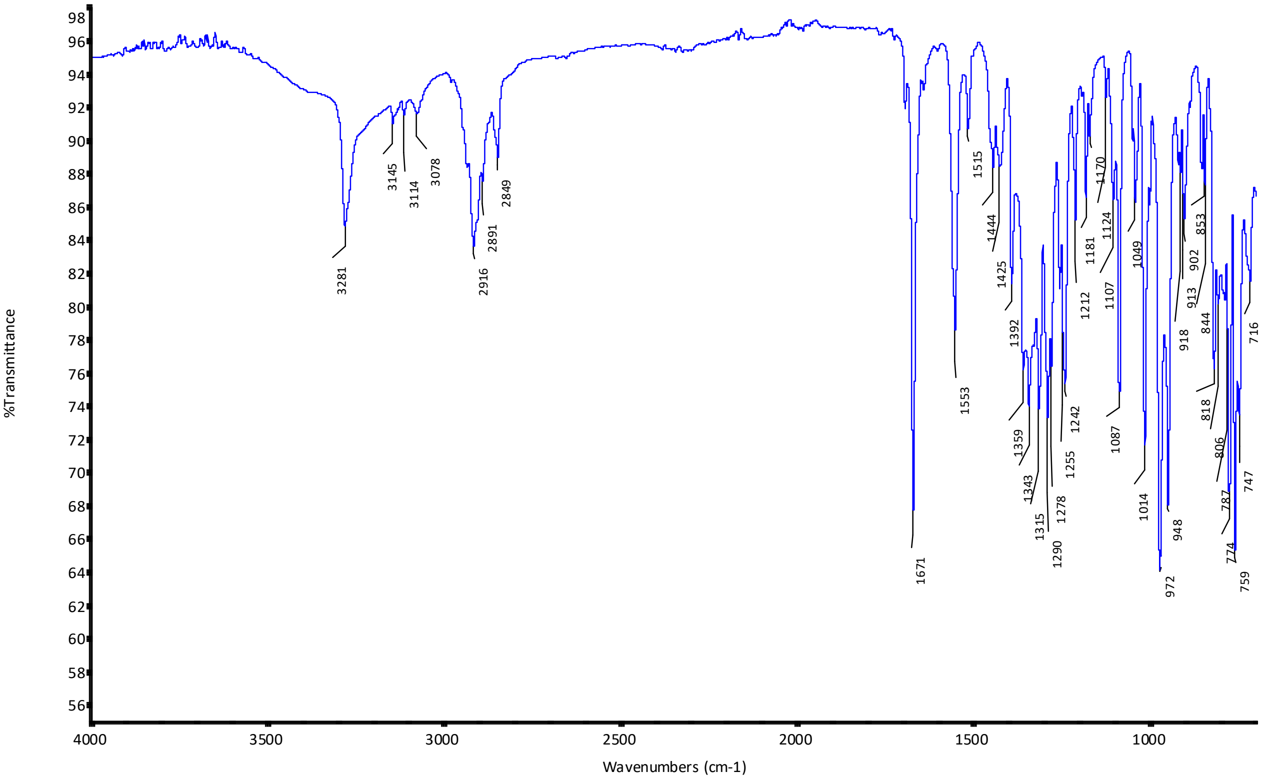

Figure S14. FT-IR spectrum of $[Ag(L^{Ad})(PTA)_2]NO_3$ (2).

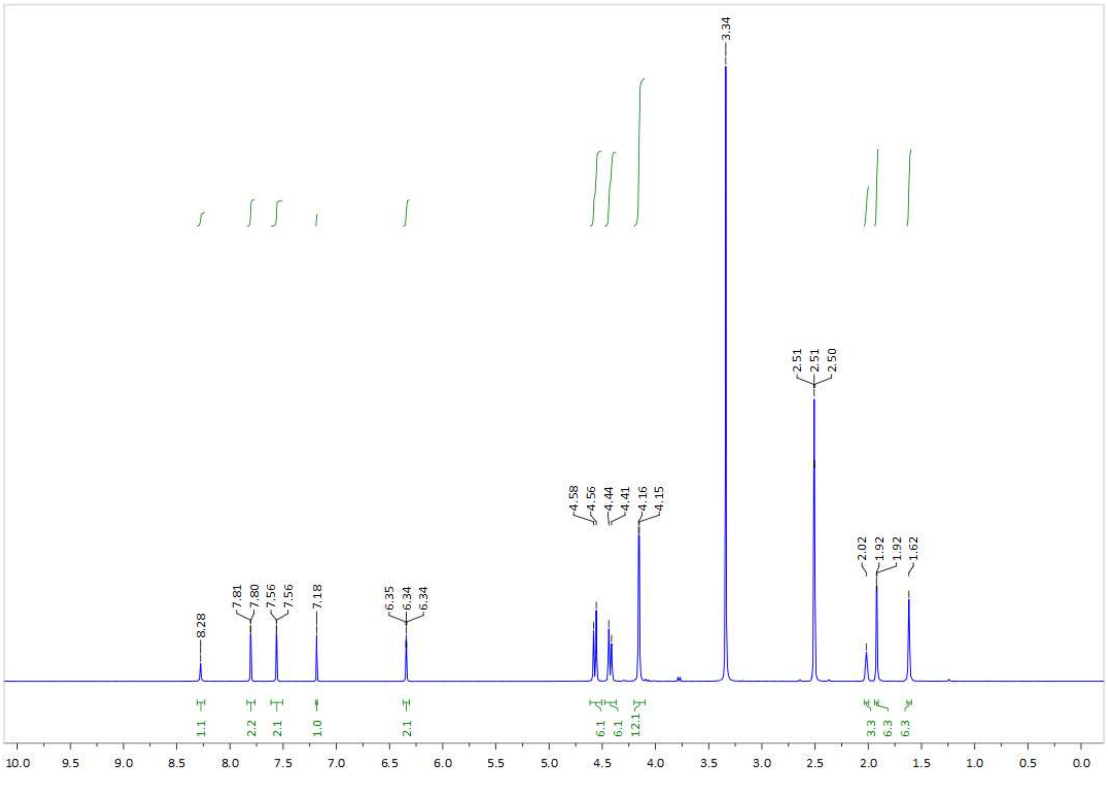

Figure S15. ^{1}H-NMR spectrum of $[Ag(L^{Ad})(PTA)_2]NO_3$ (2) in DMSO-d_6.

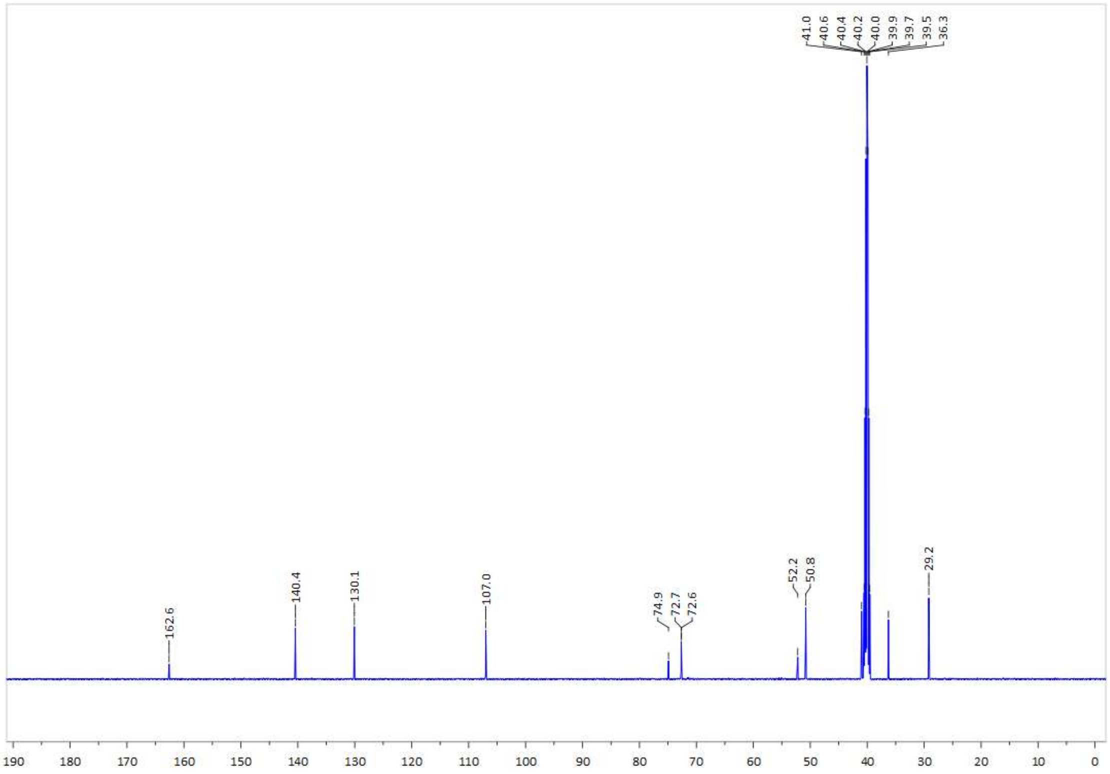

Figure S16. $^{13}C\{^1H\}$-NMR spectrum of $[Ag(L^{Ad})(PTA)_2]NO_3$ (2) in DMSO-d_6.

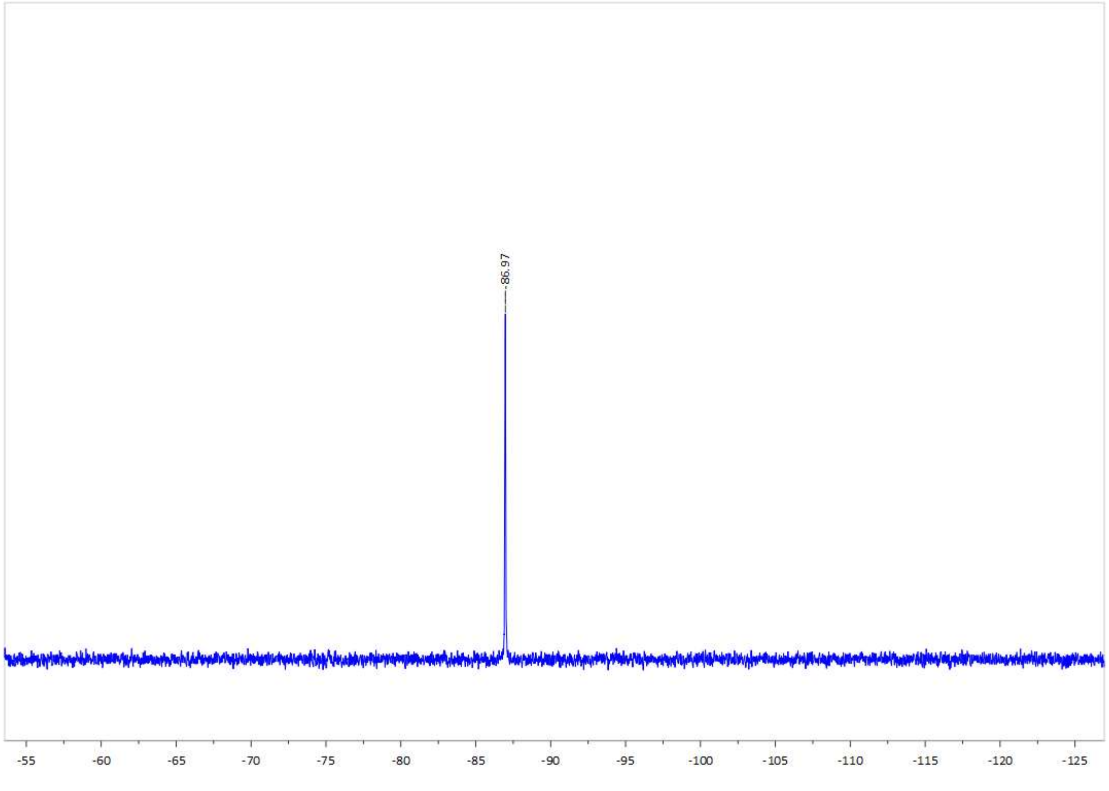

Figure S17. $^{31}P\{^1H\}$-NMR spectrum of $[Ag(L^{Ad})(PTA)_2]NO_3$ (2) in CD_3CN at 293 K.

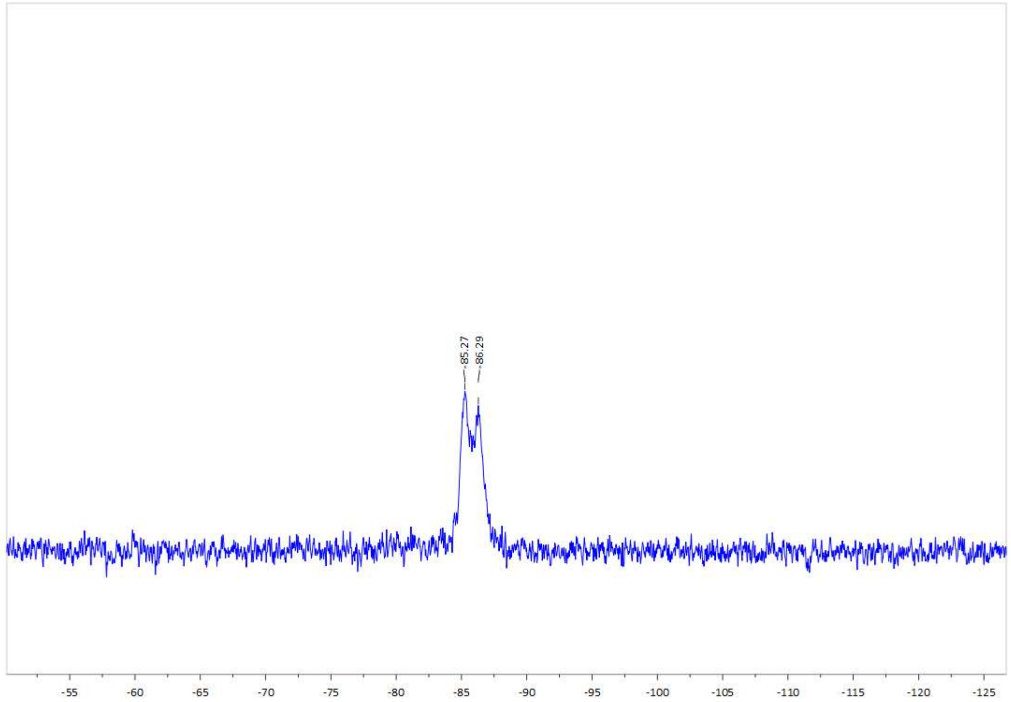

Figure S18. $^{31}P\{^1H\}$-NMR spectrum of $[Ag(L^{Ad})(PTA)_2]NO_3$ (2) in CD_3CN at 233 K.

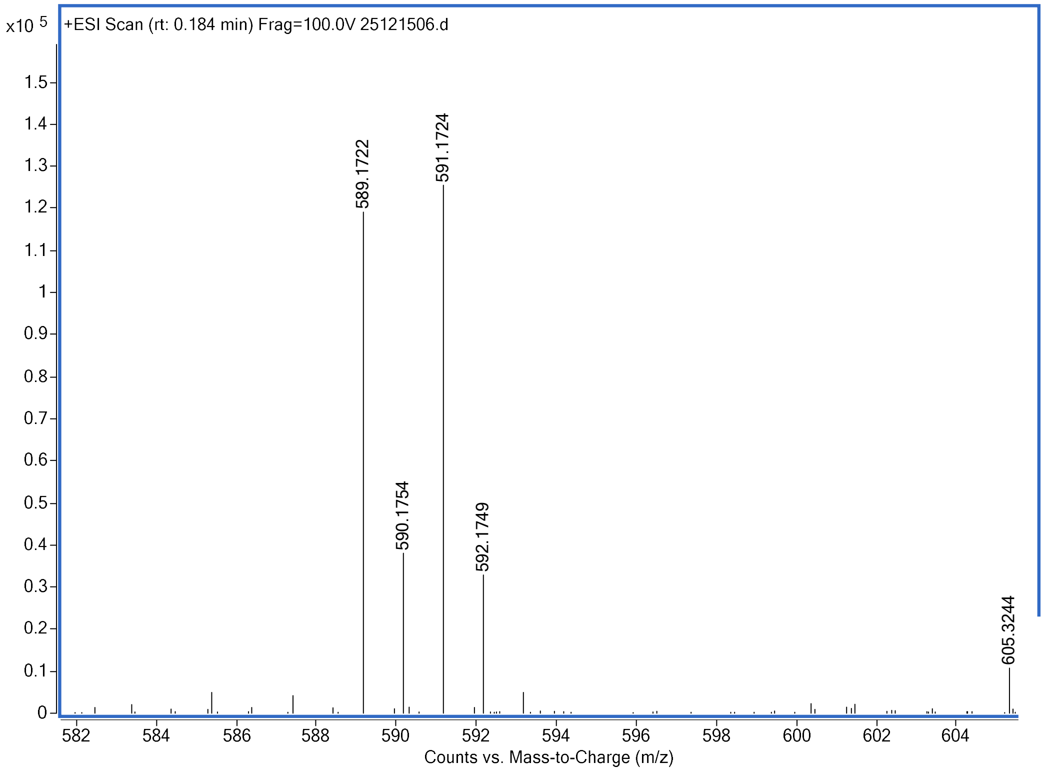

Figure S19. HR-MS spectrum of $[Ag(L^{Ad})(PTA)_2]NO_3$ (2), molecular monoisotopic mass ion peaks at m/z 589.1722 $[C_{24}H_{35}Ag^{106.9051}N_8OP]^+$ and 591.1724 $[C_{24}H_{35}Ag^{108.9048}N_8OP]^+$; HRMS-ESI(+) (Agilent 6545 – qTOF), $[M – PTA]^+$.

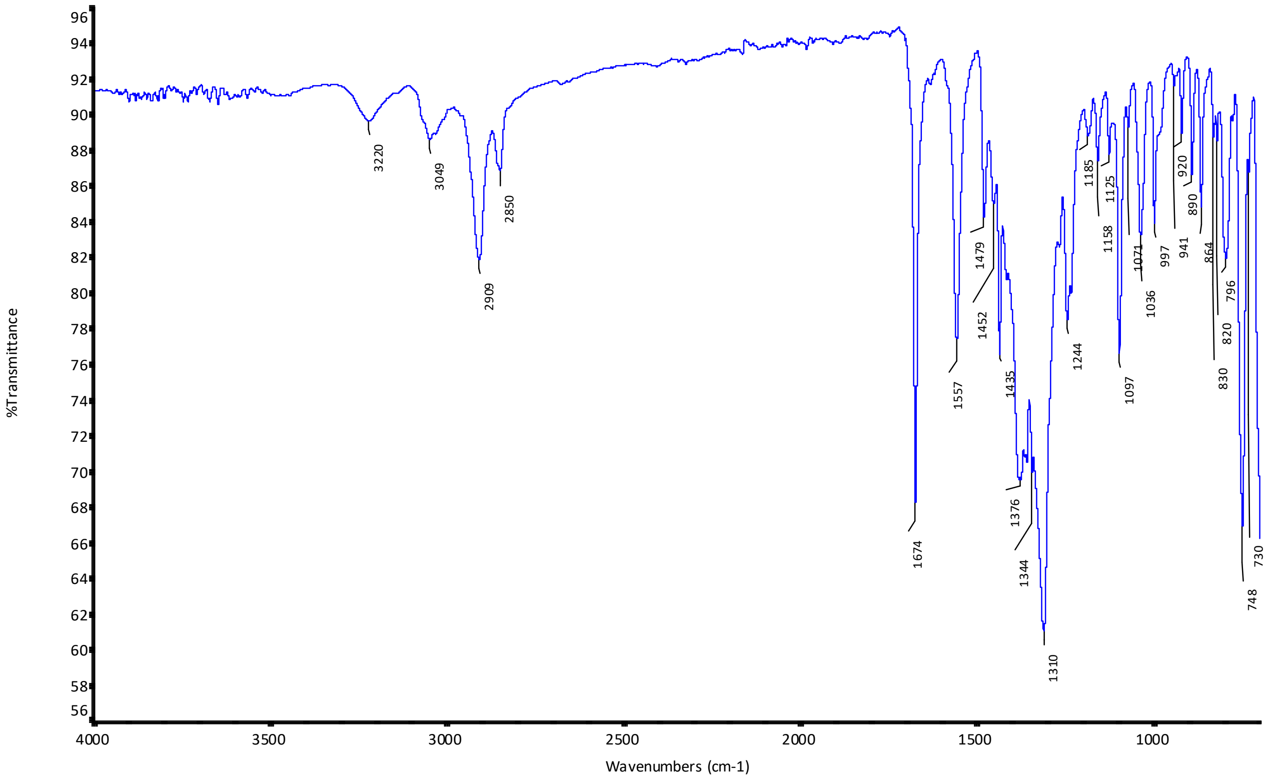

Figure S20. FT-IR spectrum of $[Ag(L^{2Ad})(PPh_3)]NO_3$·(3).

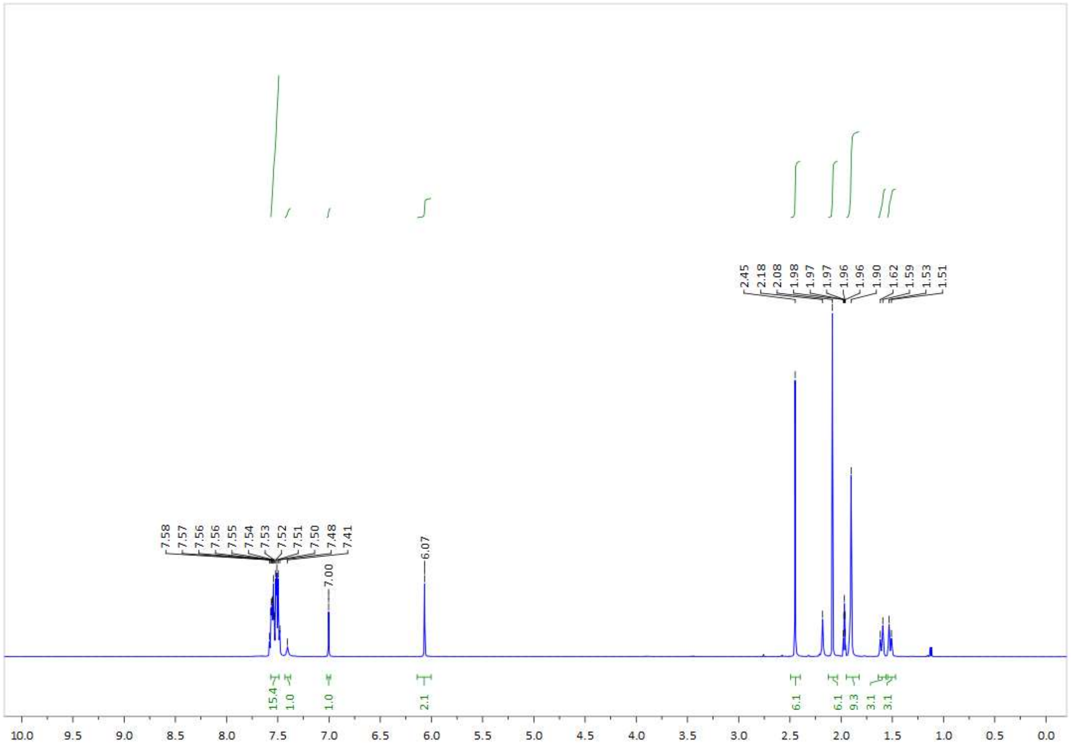

Figure S21. ^{1}H-NMR spectrum of $[Ag(L^{2Ad})(PPh_3)]NO_3$ (3) in CD_3CN.

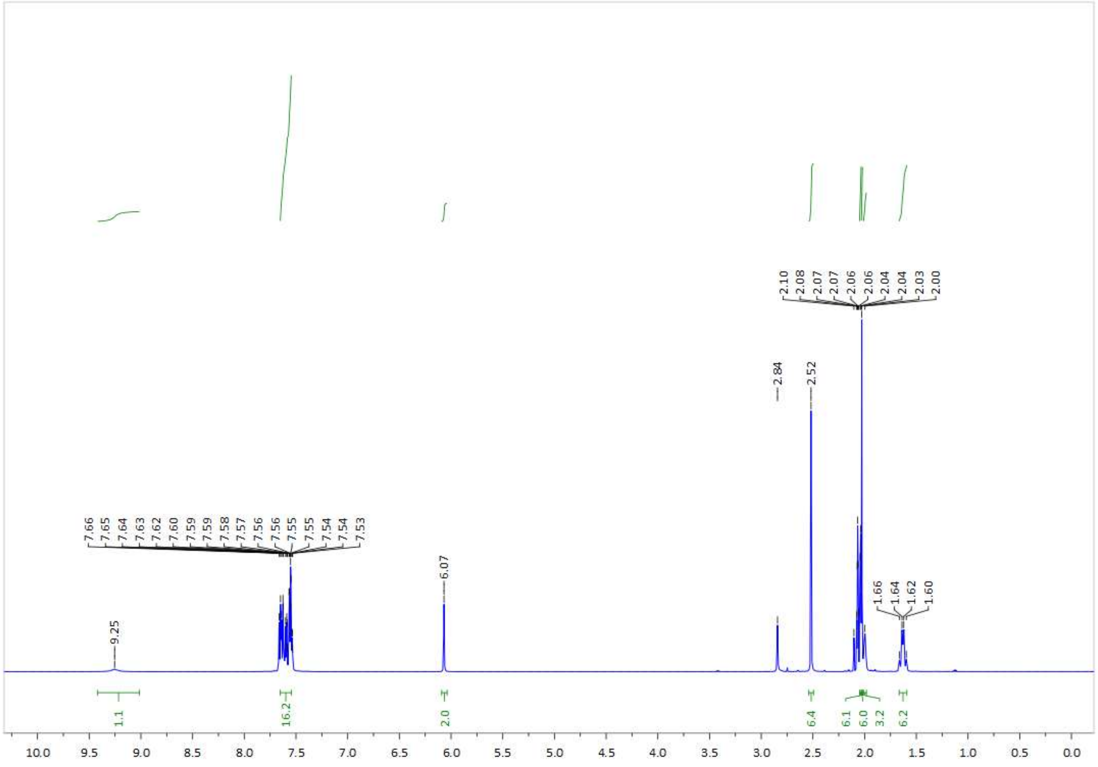

Figure S22. ^{1}H-NMR spectrum of [Ag(L^{2Ad})(PPh$_3$)]NO$_3$ (3) in acetone-d$_6$.

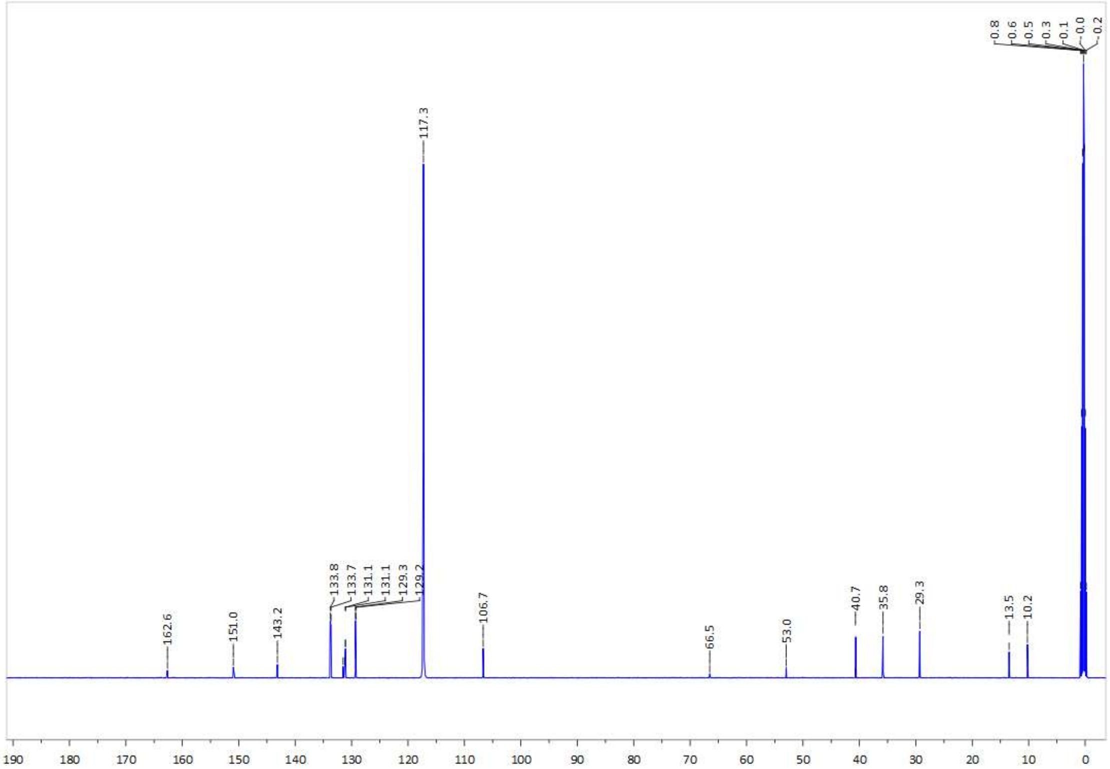

Figure S23. ^{13}C{^{1}H}-NMR spectrum of [Ag(L^{2Ad})(PPh$_3$)]NO$_3$ (3) in CD$_3$CN.

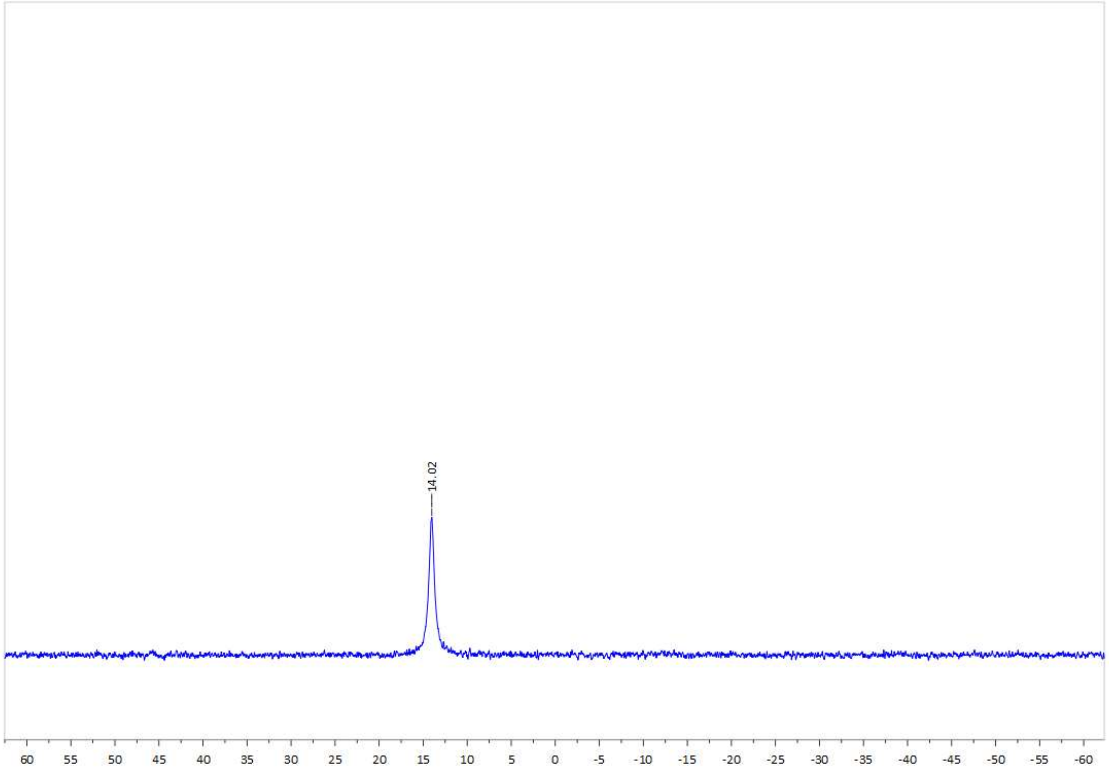

Figure S24. $^{31}P\{^{1}H\}$-NMR spectrum of $[Ag(L^{2Ad})(PPh_3)]NO_3$ (3) in CD_3CN at 293 K.

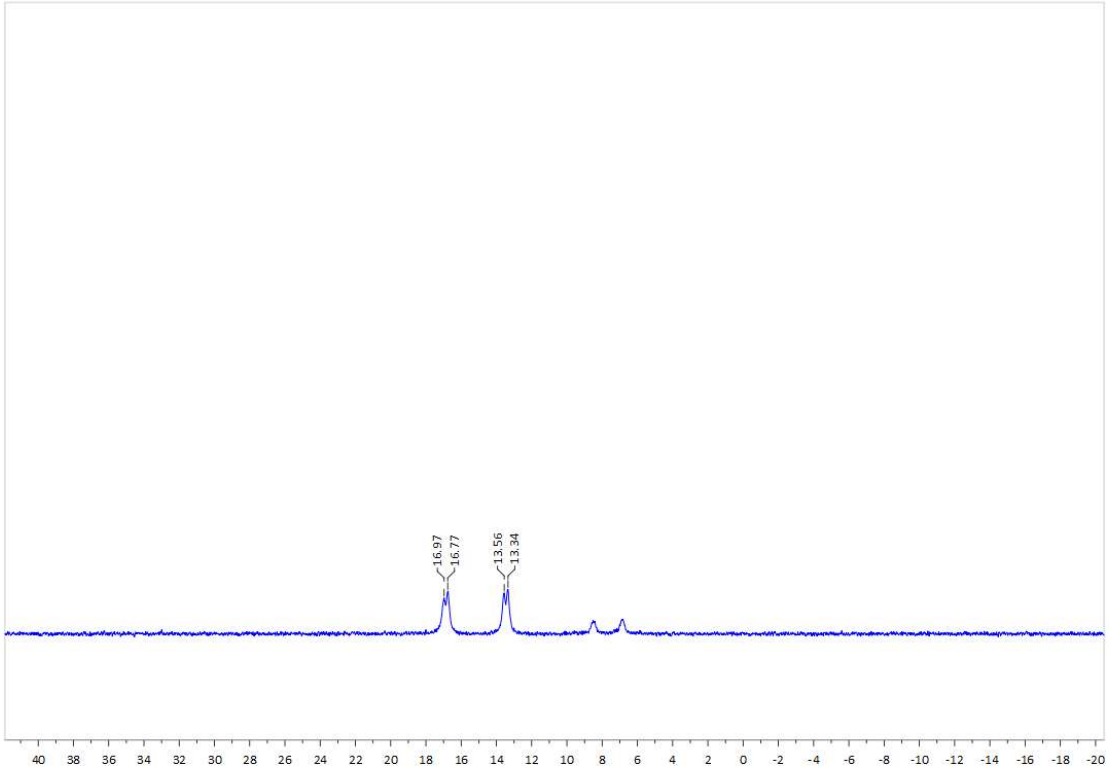

Figure S25. $^{31}P\{^{1}H\}$-NMR spectrum of $[Ag(L^{2Ad})(PPh_3)]NO_3$ (3) in CD_3OD at 223 K.

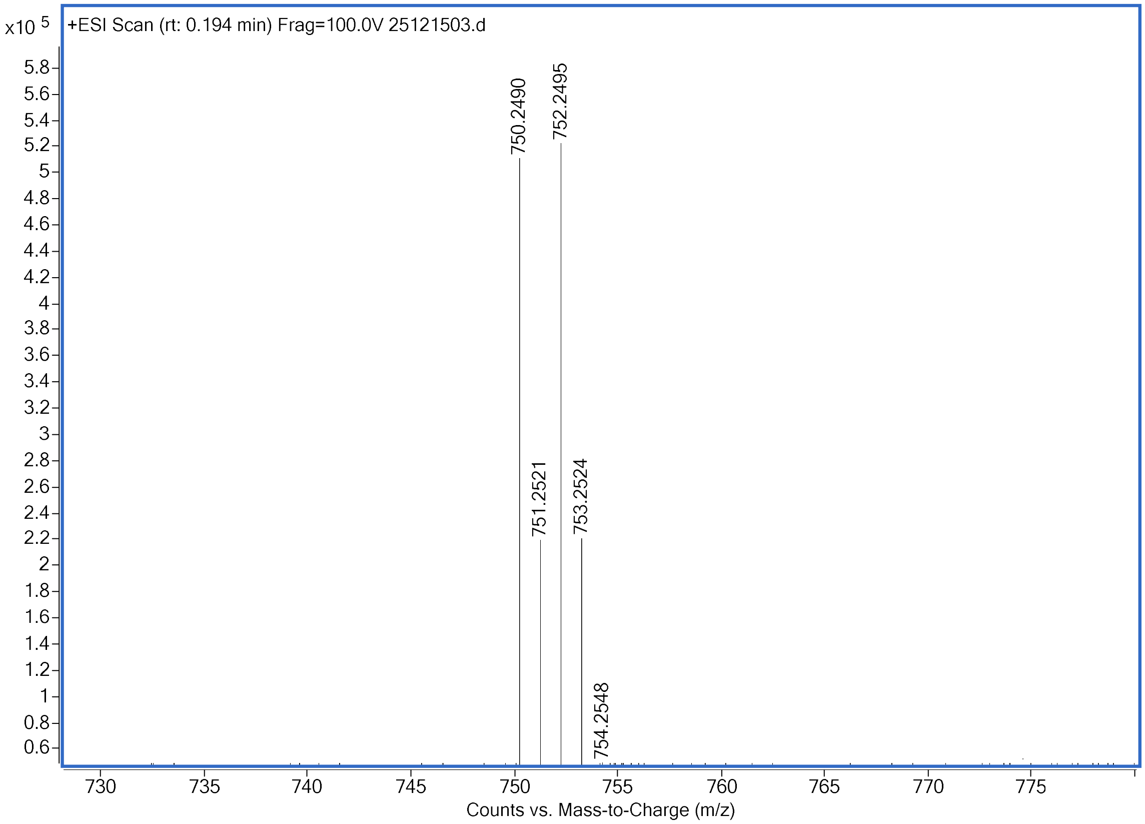

Figure S26. HR-MS spectrum of $[Ag(L^{2Ad})(PPh_3)]NO_3$ (3), molecular monoisotopic mass ion peaks at m/z 750.2490 $[C_{40}H_{46}Ag^{106.9051}N_5OP]^+$ and 752.2495 $[C_{40}H_{46}Ag^{108.9048}N_5OP]^+$; HRMS-ESI(+) (Agilent 6545 – qTOF), $[M]^+$.

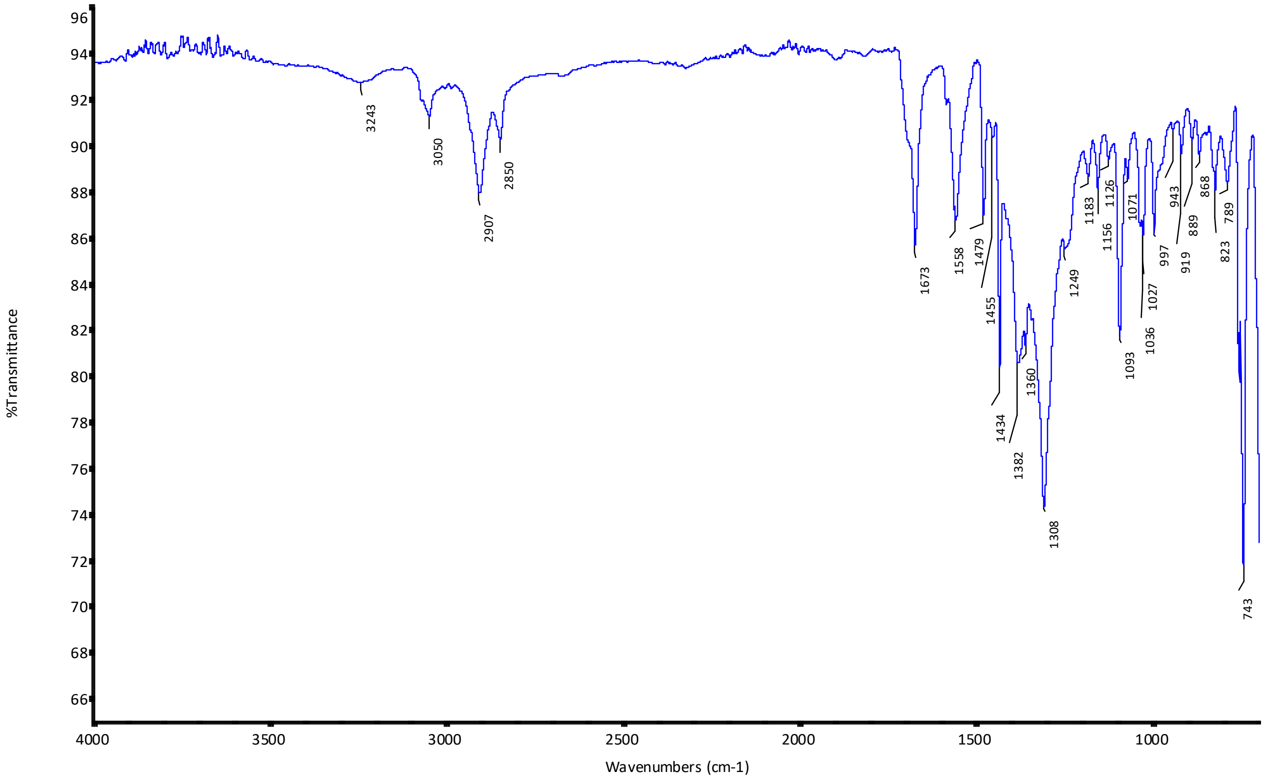

Figure S27. FT-IR spectrum of $[Ag(L^{2Ad})(PPh_3)_2]NO_3$ (4).

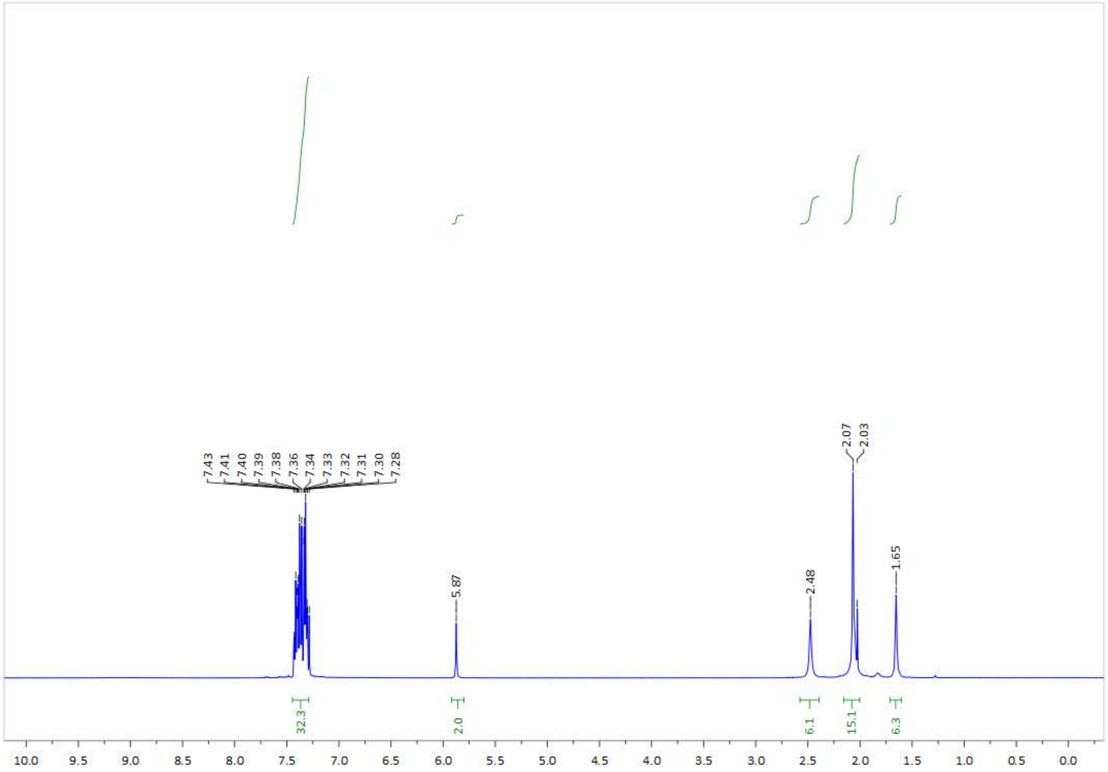

Figure S28. ^{1}H-NMR spectrum of $[Ag(L^{2Ad})(PPh_3)_2]NO_3$ (4) in $CDCl_3$.

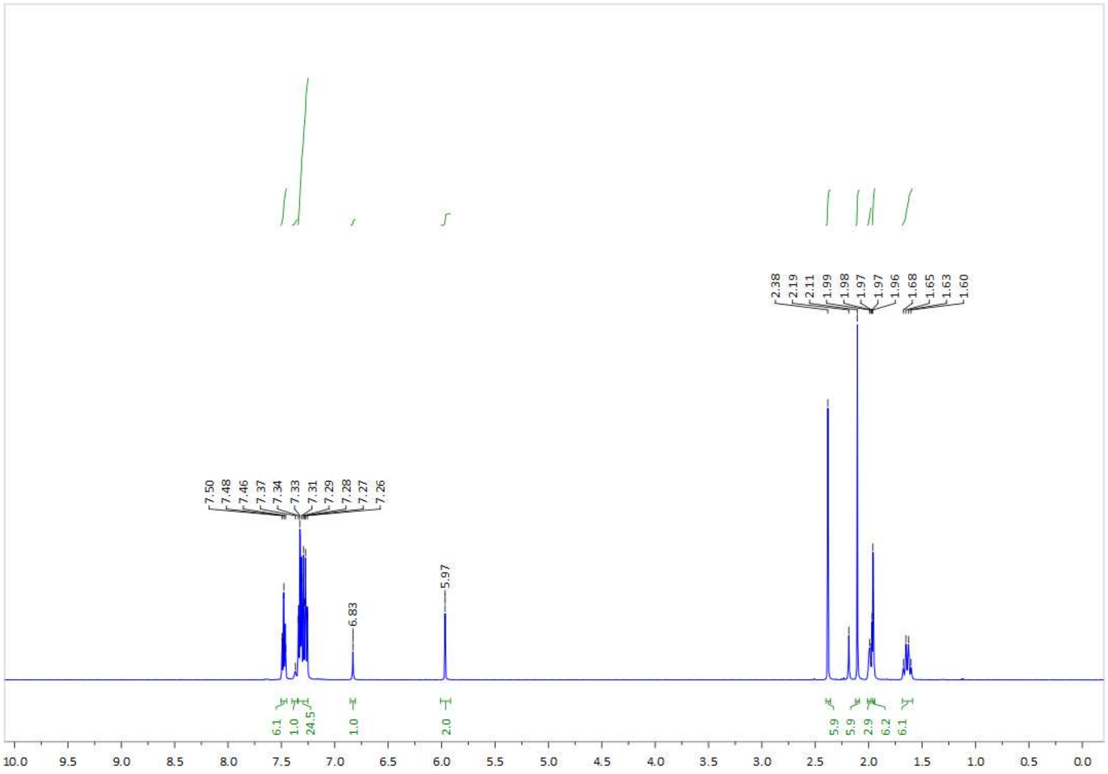

Figure S29. ^{1}H-NMR spectrum of $[Ag(L^{2Ad})(PPh_3)_2]NO_3$ (4) in CD_3CN.

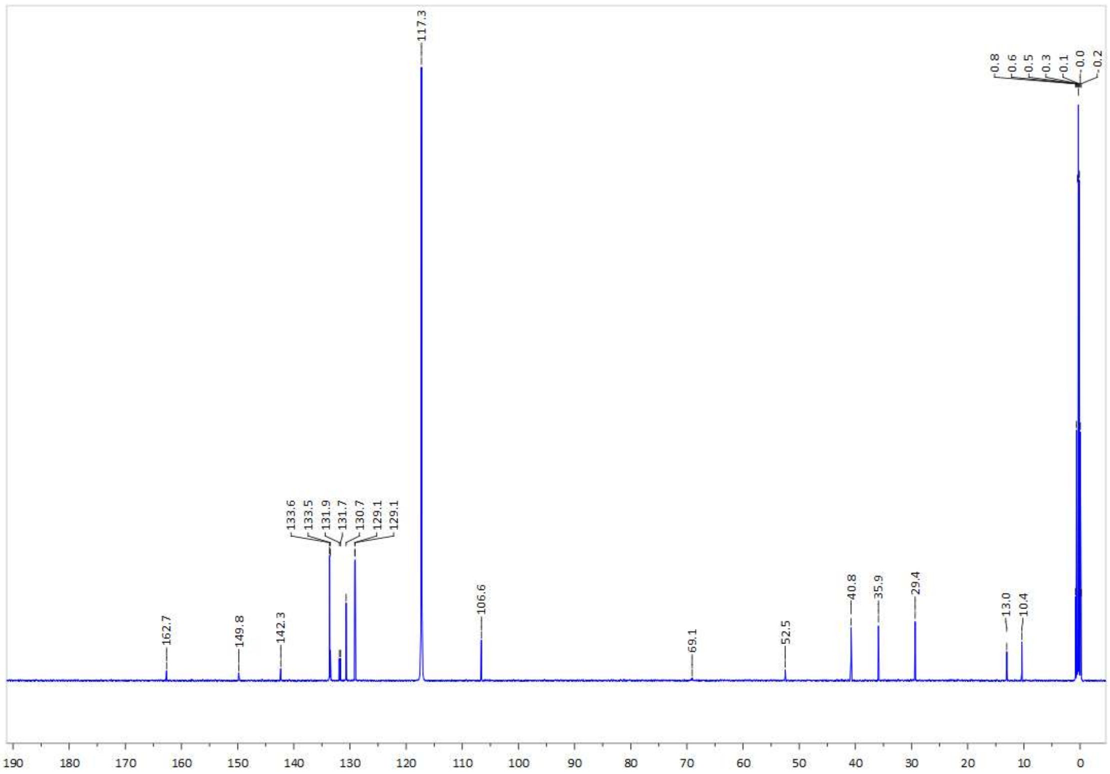

Figure S30. $^{13}C\{^{1}H\}$-NMR spectrum of $[Ag(L^{2Ad})(PPh_3)_2]NO_3$ (4) in CD_3CN.

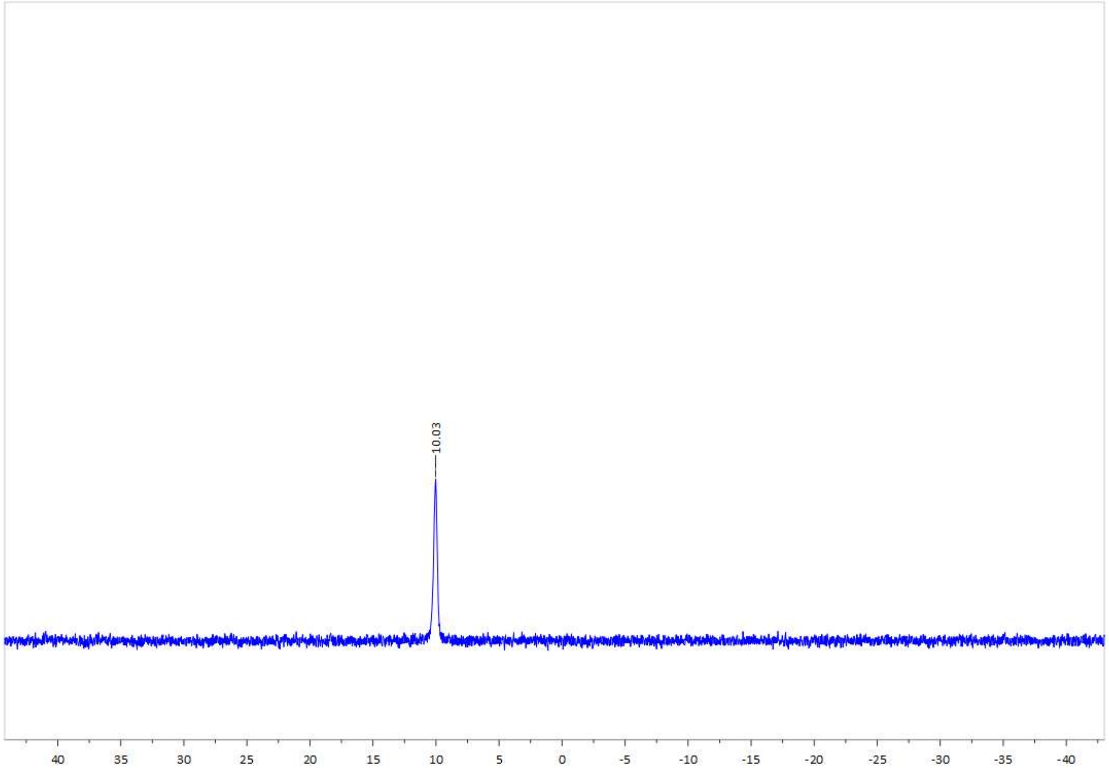

Figure S31. $^{31}P\{^{1}H\}$-NMR spectrum of $[Ag(L^{2Ad})(PPh_3)_2]NO_3$ (4) in CD_3CN at 293 K.

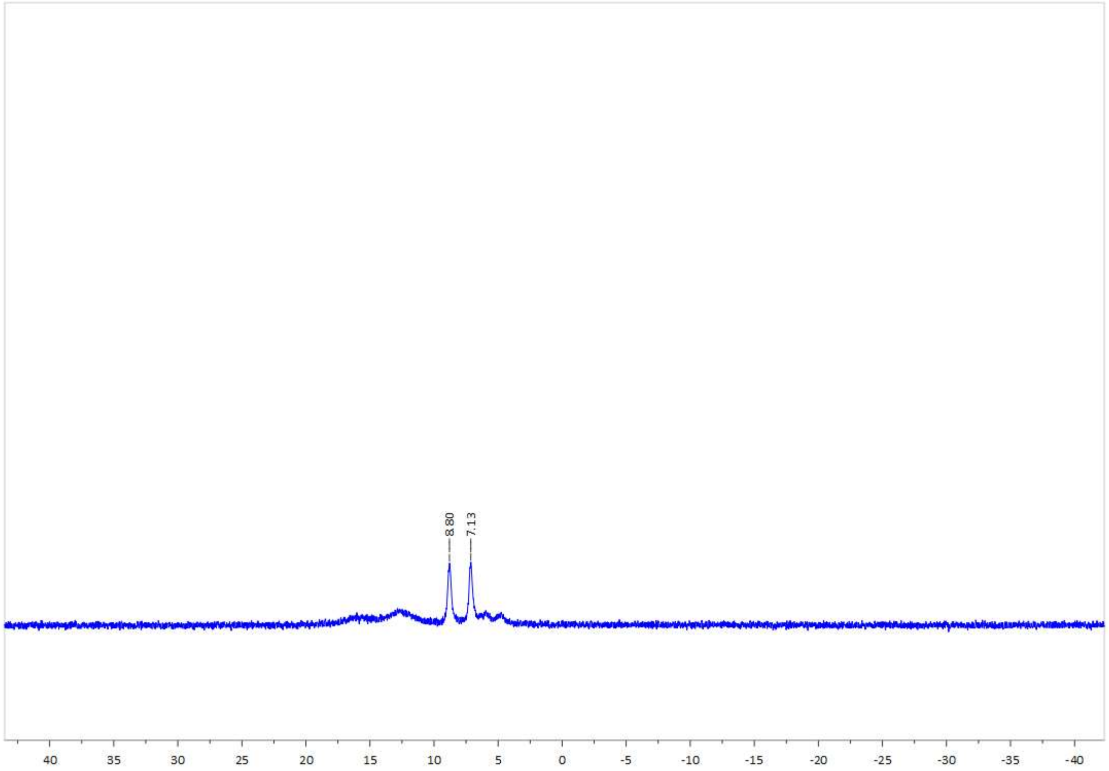

Figure S32. $^{31}P\{^{1}H\}$-NMR spectrum of $[Ag(L^{2Ad})(PPh_3)_2]NO_3$ (4) in CD_3OD at 223 K.

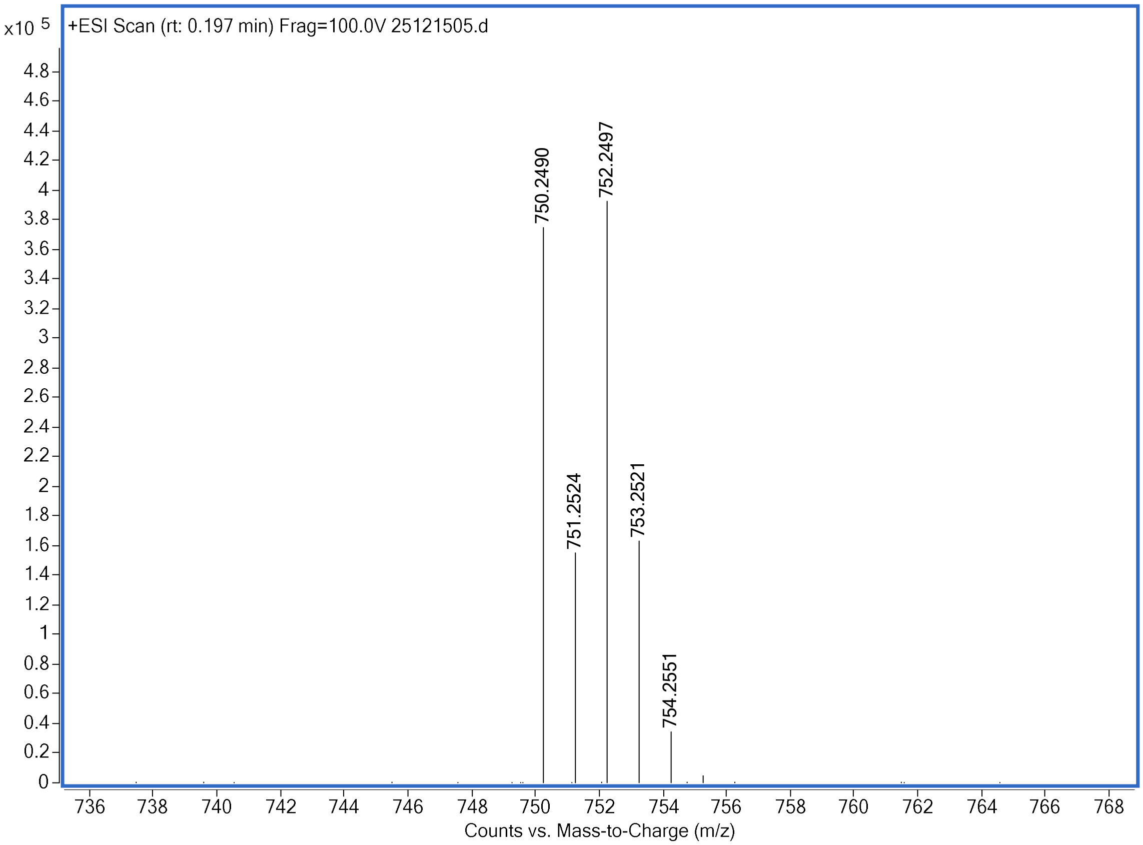

Figure S33. HR-MS spectrum of $[Ag(L^{2Ad})(PPh_3)_2]NO_3$ (4), molecular monoisotopic mass ion peaks at m/z 750.2490 $[C_{40}H_{46}Ag^{106.9051}N_5OP]^+$ and 752.2497 $[C_{40}H_{46}Ag^{108.9048}N_5OP]^+$; HRMS-ESI(+) (Agilent 6545 – qTOF), $[M – PPh_3]^+$.

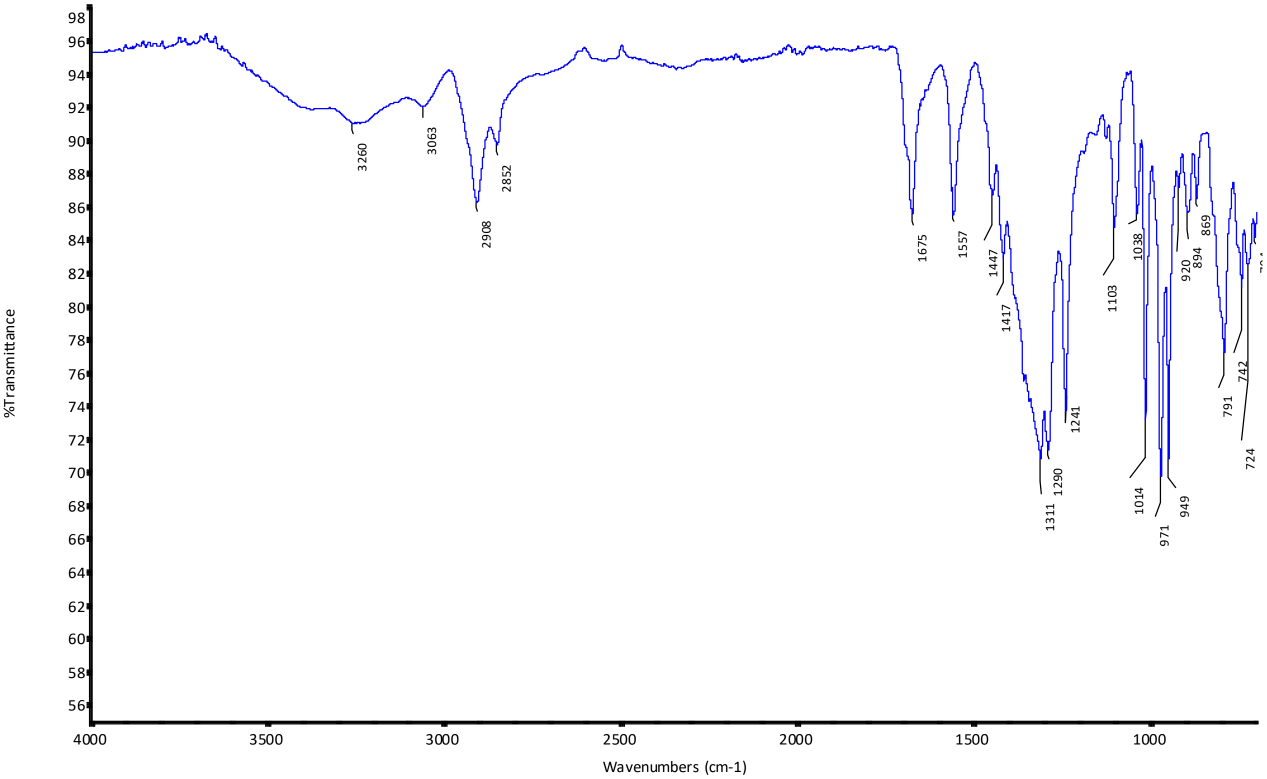

Figure S34. FT-IR spectrum of $[Ag(L^{2Ad})(PTA)]NO_3$ (5).

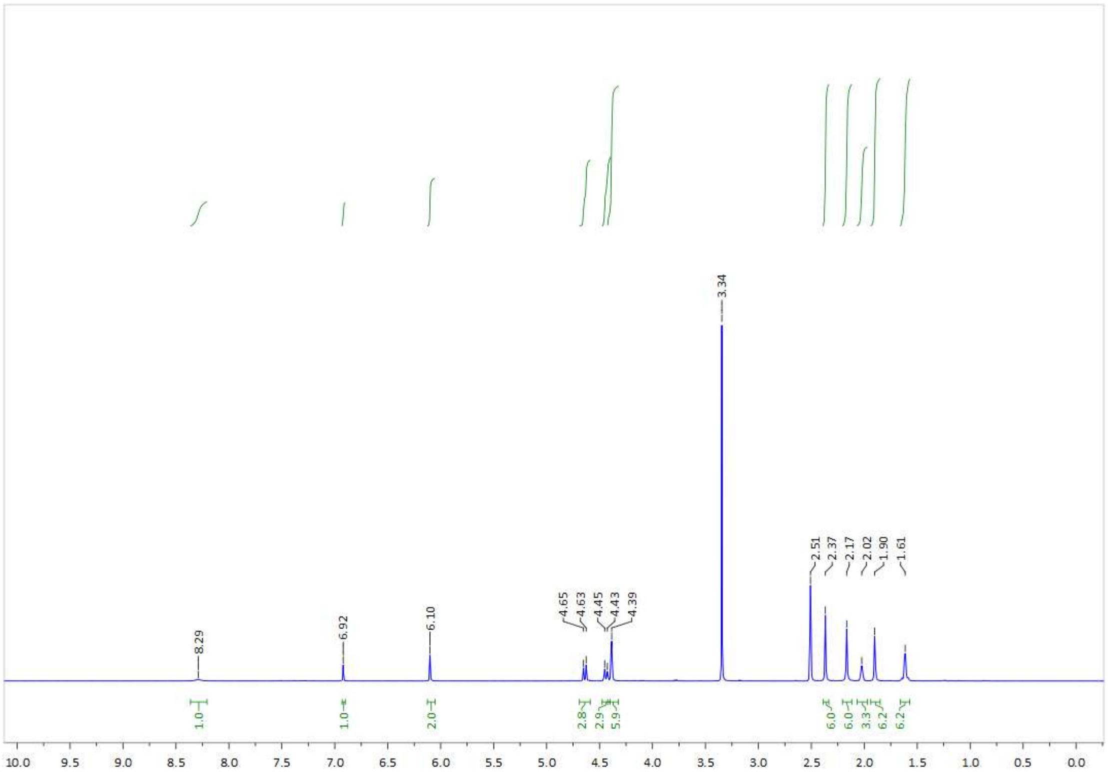

Figure S35. ^{1}H-NMR spectrum of $[Ag(L^{2Ad})(PTA)]NO_3$ (5) in DMSO-d_6.

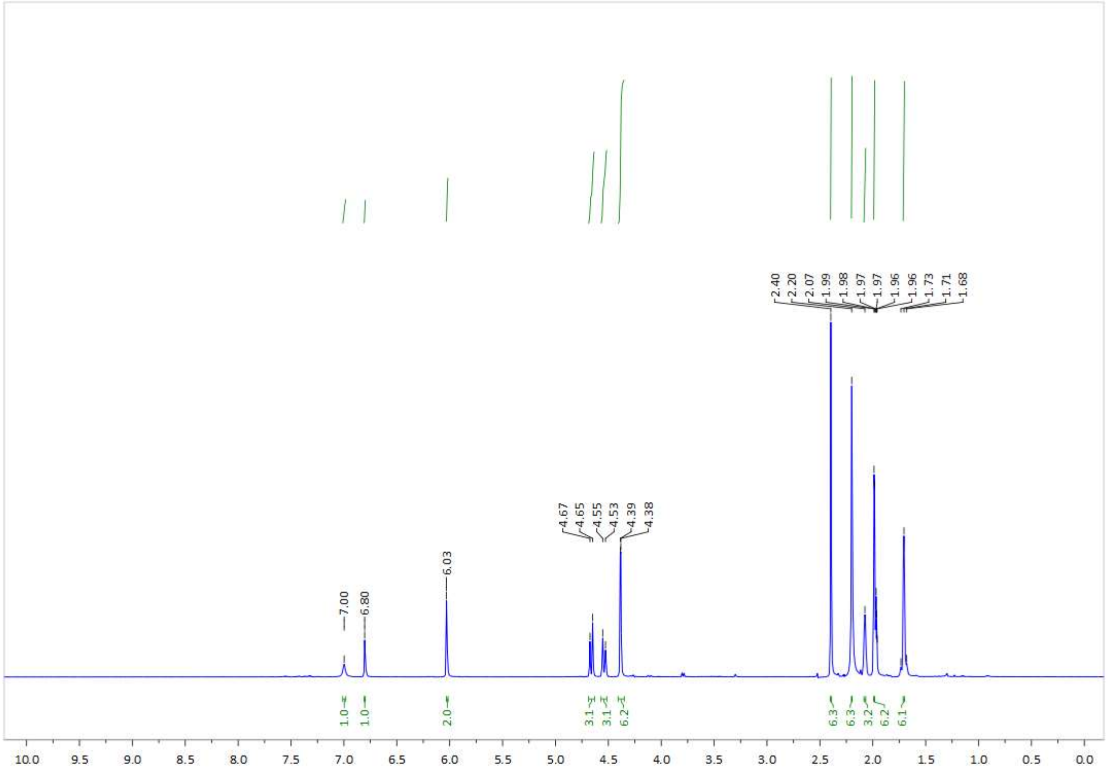

Figure S36. ^{1}H-NMR spectrum of [Ag(L^{2Ad})(PTA)]NO_3 (5) in CD_3CN.

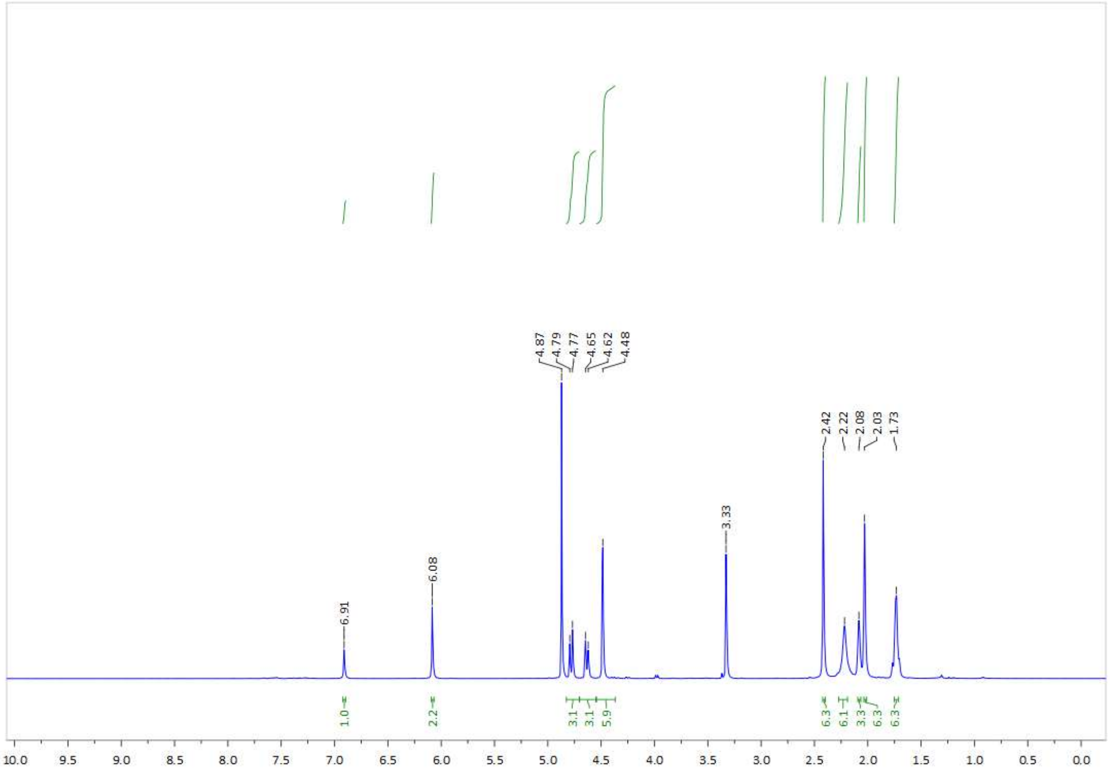

Figure S37. ^{1}H-NMR spectrum of [Ag(L^{2Ad})(PTA)]NO_3 (5) in CD_3OD.

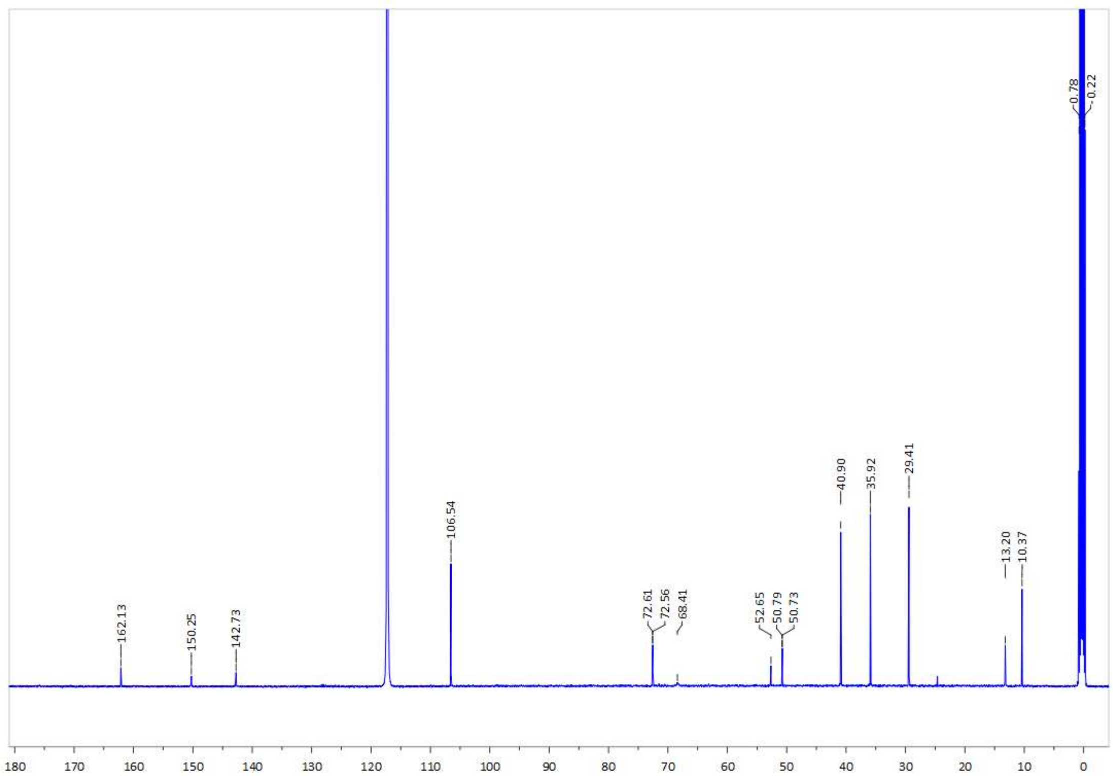

Figure S38. $^{13}C\{^{1}H\}$-NMR spectrum of $[Ag(L^{2Ad})(PTA)]NO_3$ (5) in CD_3CN.

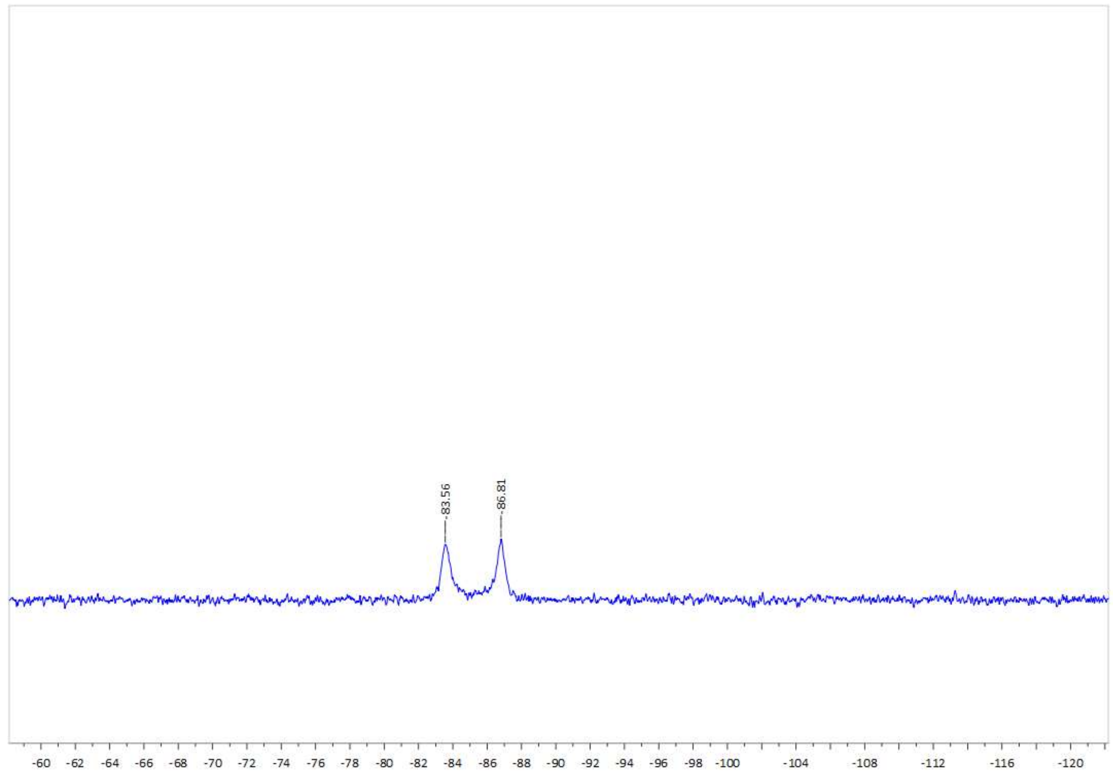

Figure S39. $^{31}P\{^{1}H\}$-NMR spectrum of $[Ag(L^{2Ad})(PTA)]NO_3$ (5) in CD_3CN at 293 K.

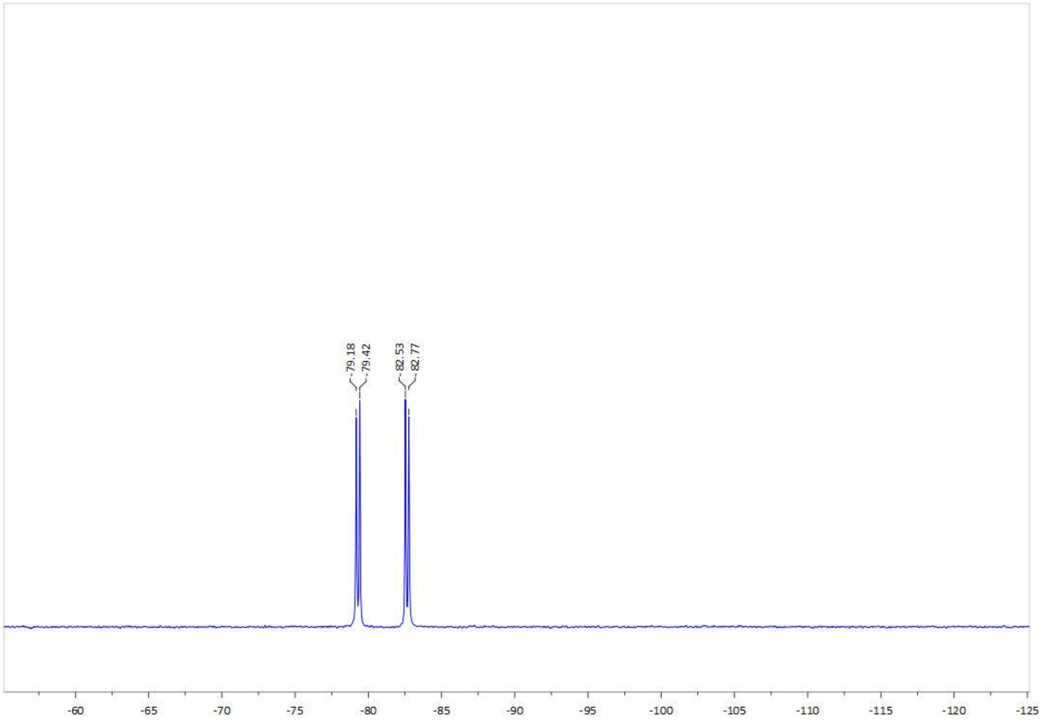

Figure S40. $^{31}P\{^1H\}$-NMR spectrum of $[Ag(L^{2Ad})(PTA)]NO_3$ (5) in CD_3OD at 223 K.

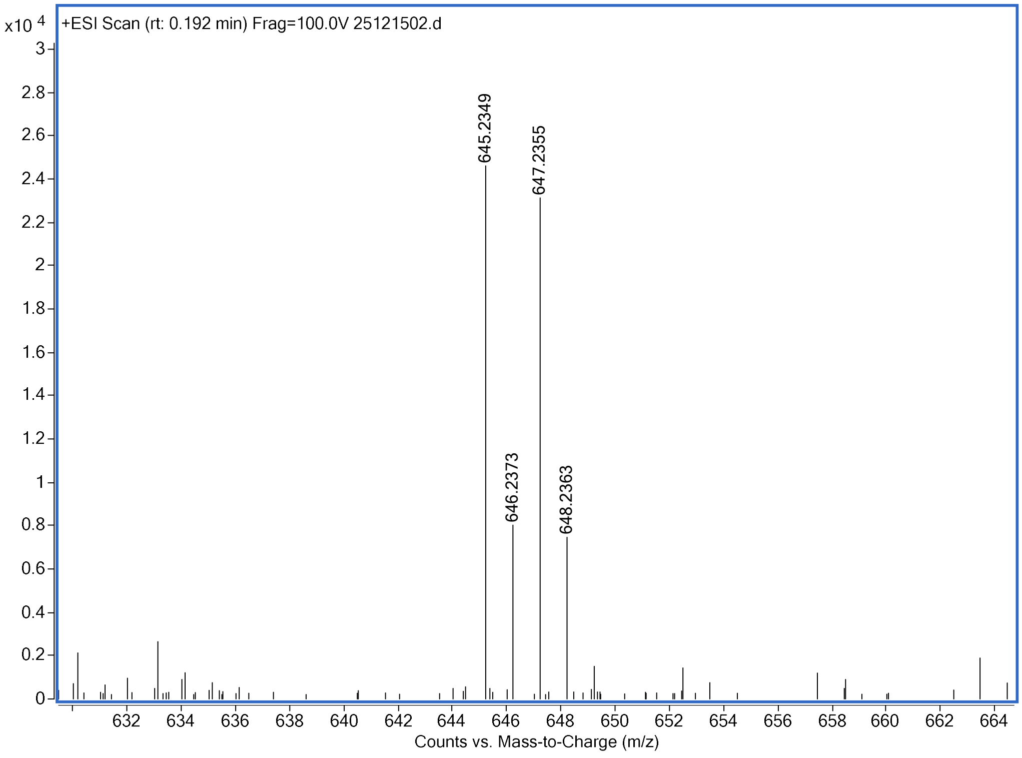

Figure S41. HR-MS spectrum of $[Ag(L^{2Ad})(PTA)]NO_3$ (5), molecular monoisotopic mass ion peaks at m/z 645.2349 $[C_{28}H_{43}Ag^{106.9051}N_8OP]^+$ and 647.2355 $[C_{28}H_{43}Ag^{108.9048}N_8OP]^+$; HRMS-ESI(+) (Agilent 6545 – qTOF), $[M]^+$.

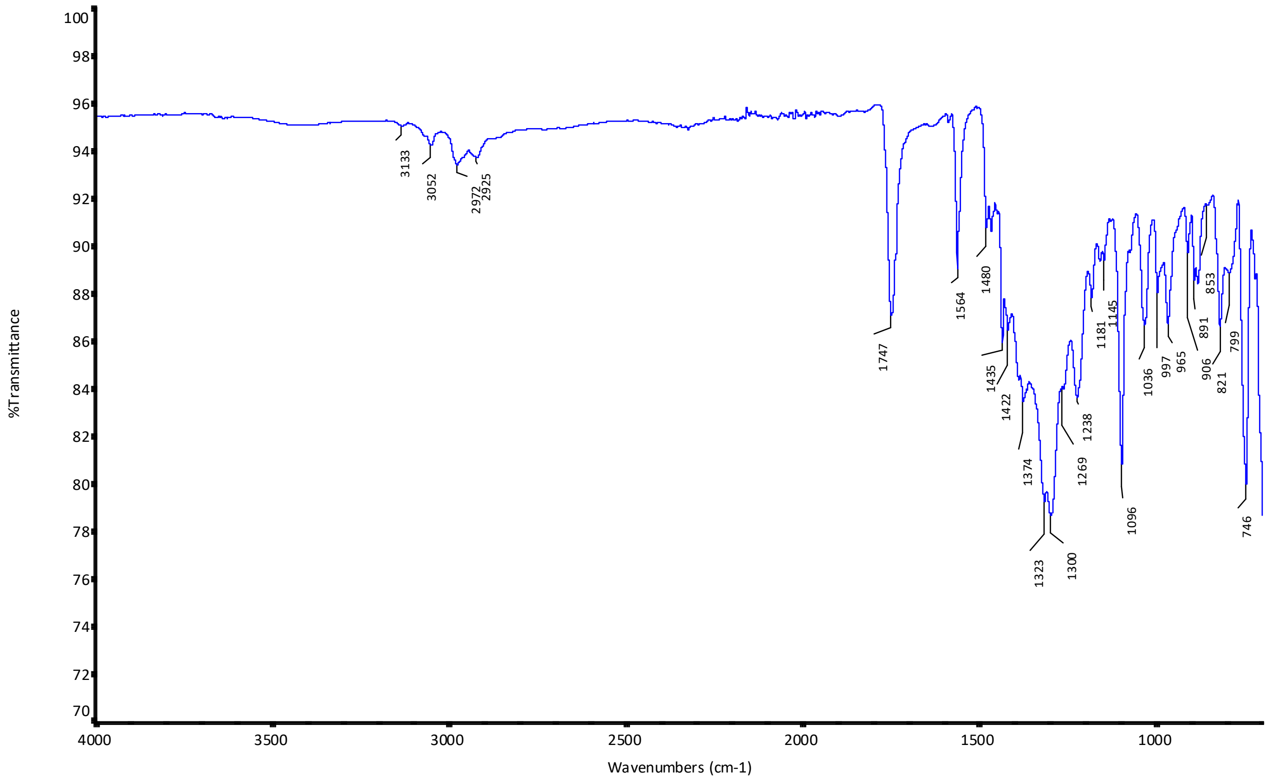

Figure S42. FT-IR spectrum of $[Ag(L^{2OiPr})(PPh_3)]NO_3$ (6).

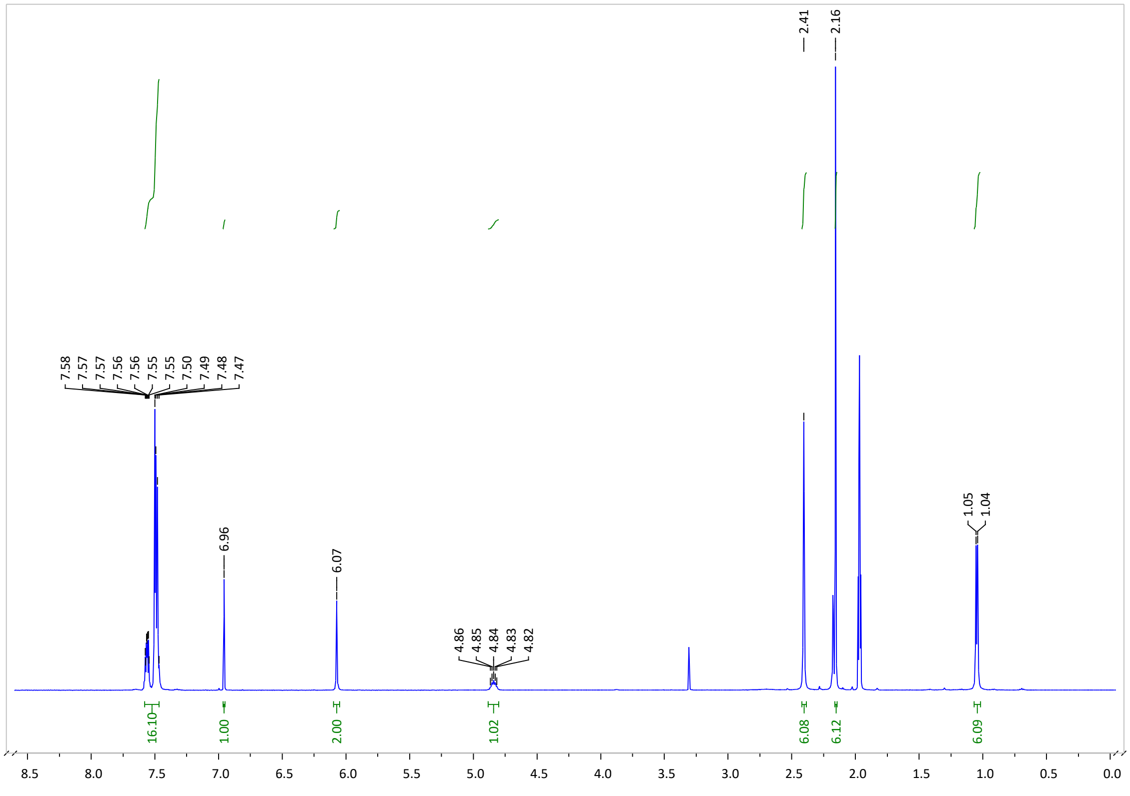

Figure S43. ^{1}H-NMR spectrum of $[Ag(L^{2OiPr})(PPh_3)]NO_3$ (6) in CD_3CN.

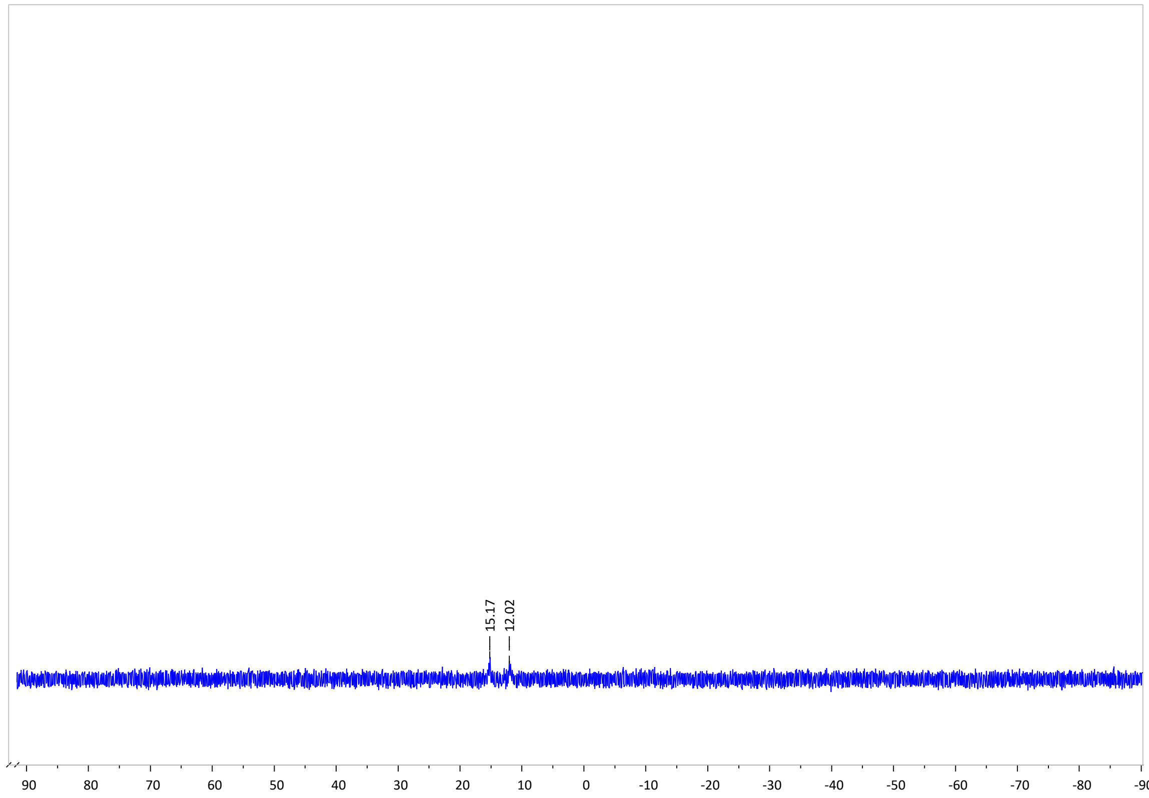

Figure S44. ^{31}P-NMR spectrum of [Ag(L^{2OiPr})(PPh$_3$)]NO$_3$ (6) in CD$_3$CN at 293 K.

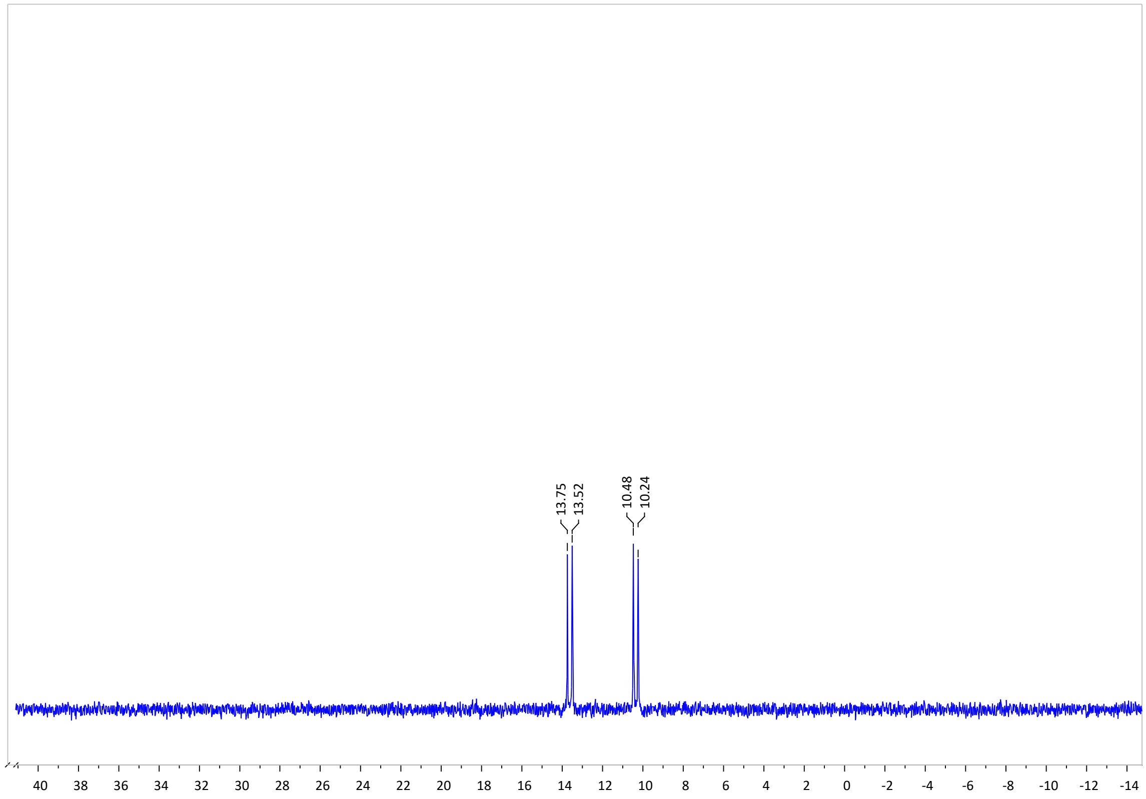

Figure S45. ^{31}P-NMR spectrum of [Ag(L^{2OiPr})(PPh$_3$)]NO$_3$ (6) in CD$_3$CN at 243 K.

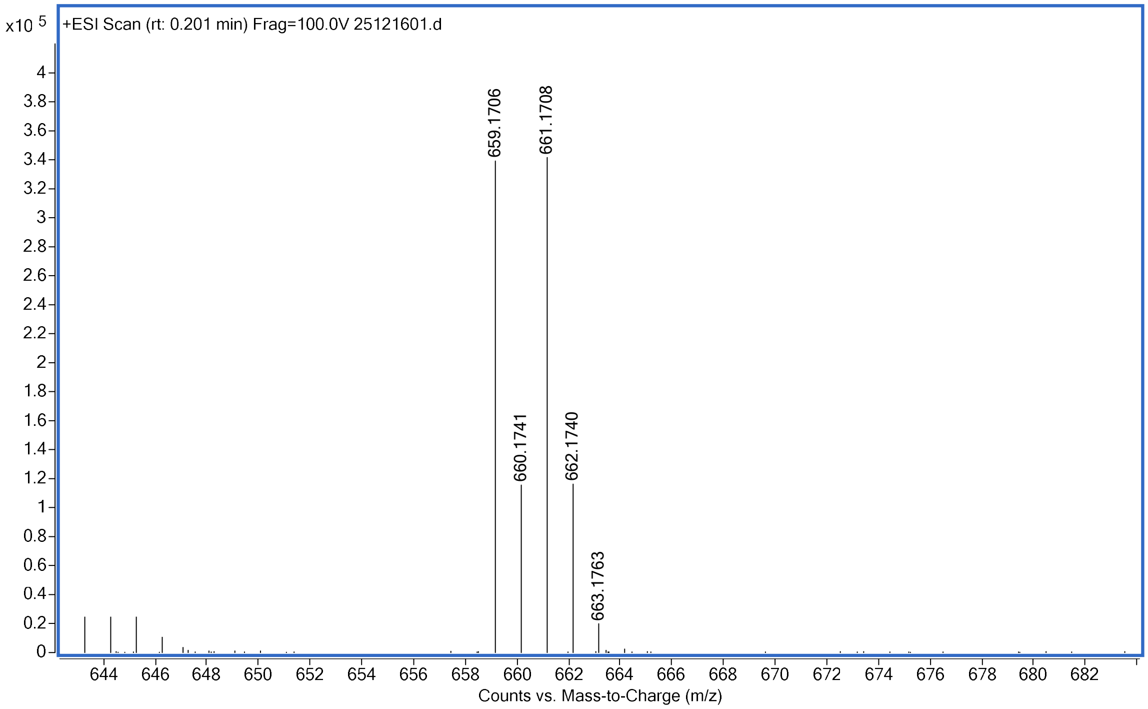

Figure S46. HR-MS spectrum of $[Ag(L^{2OiPr})(PPh_3)]NO_3$ (6), molecular monoisotopic mass ion peaks at m/z 659.1706 $[C_{33}H_{37}Ag^{106.9051}N_4O_2P]^+$ and 661.1708 $[C_{33}H_{37}Ag^{108.9048}N_4O_2P]^+$; HRMS-ESI(+) (Agilent 6545 – qTOF), $[M]^+$.

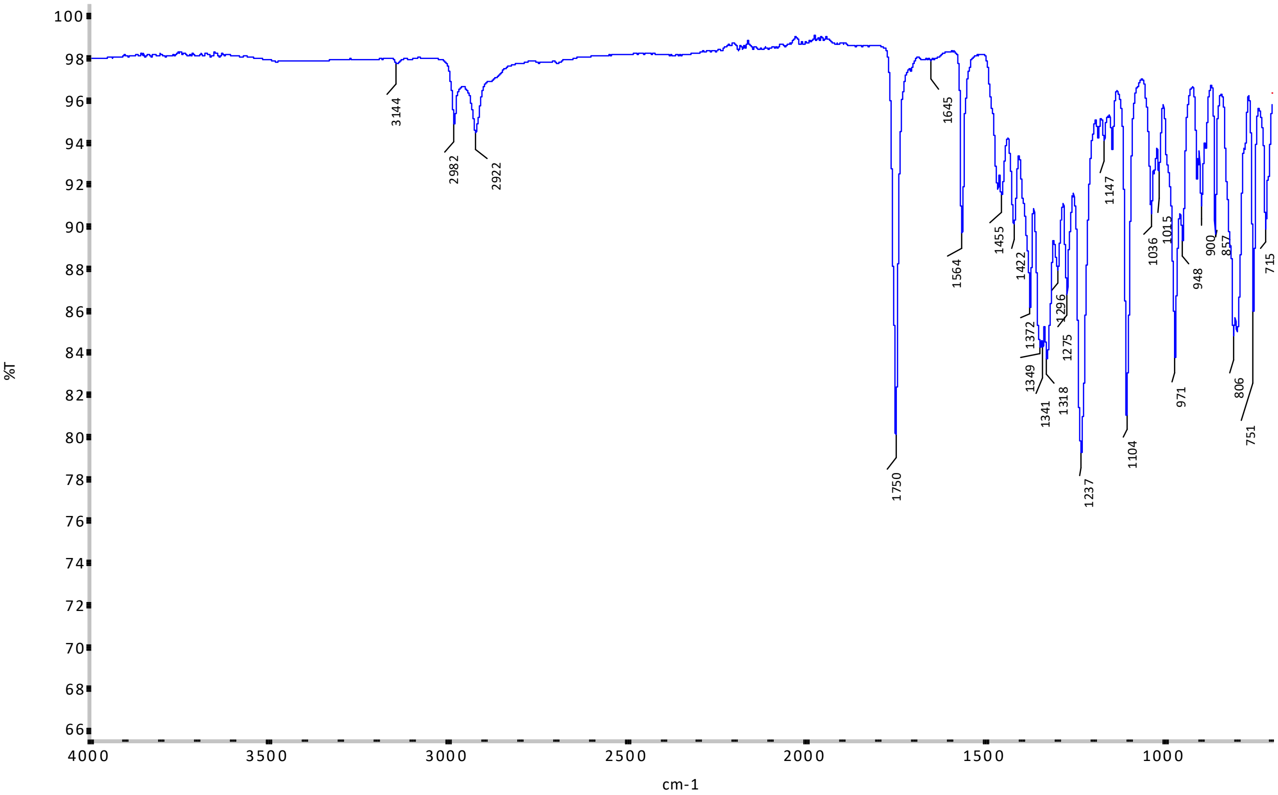

Figure S47. FT-IR spectrum of $[Ag(L^{2OiPr})(PTA)]NO_3$ (7).

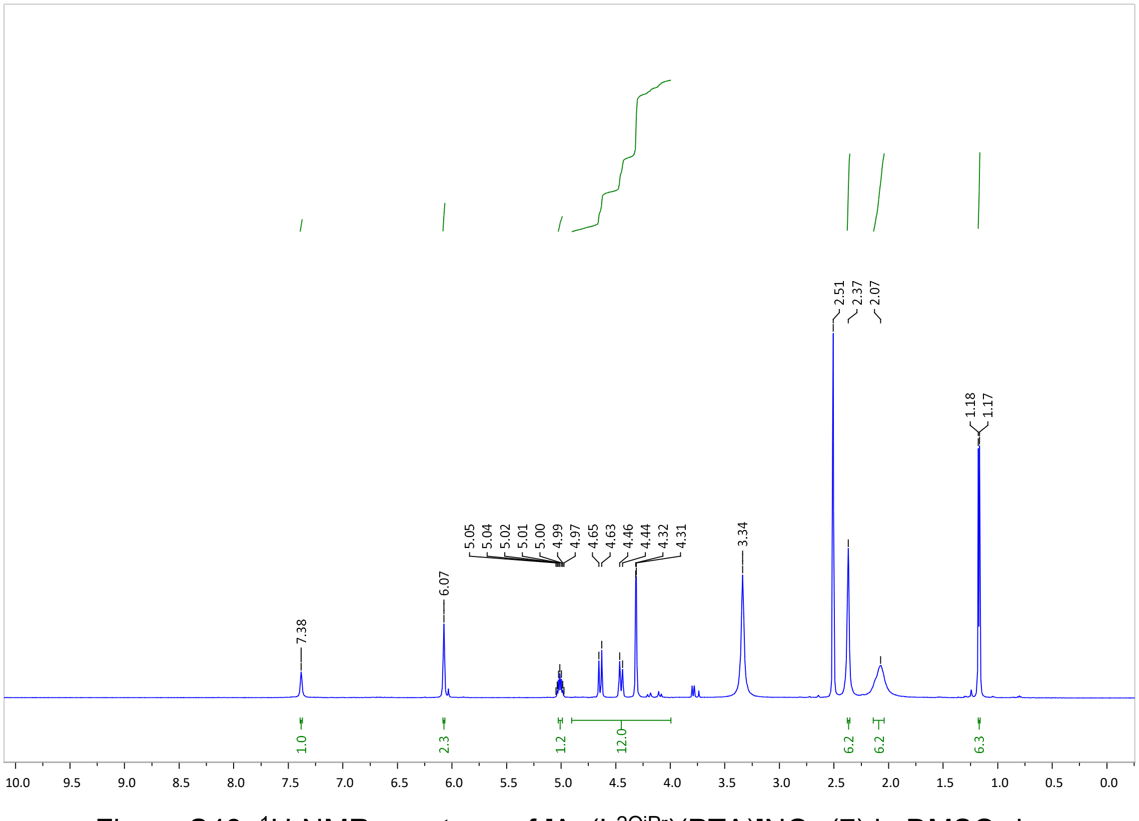

Figure S48. ^{1}H-NMR spectrum of $[Ag(L^{2OiPr})(PTA)]NO_3$ (7) in DMSO-d_6.

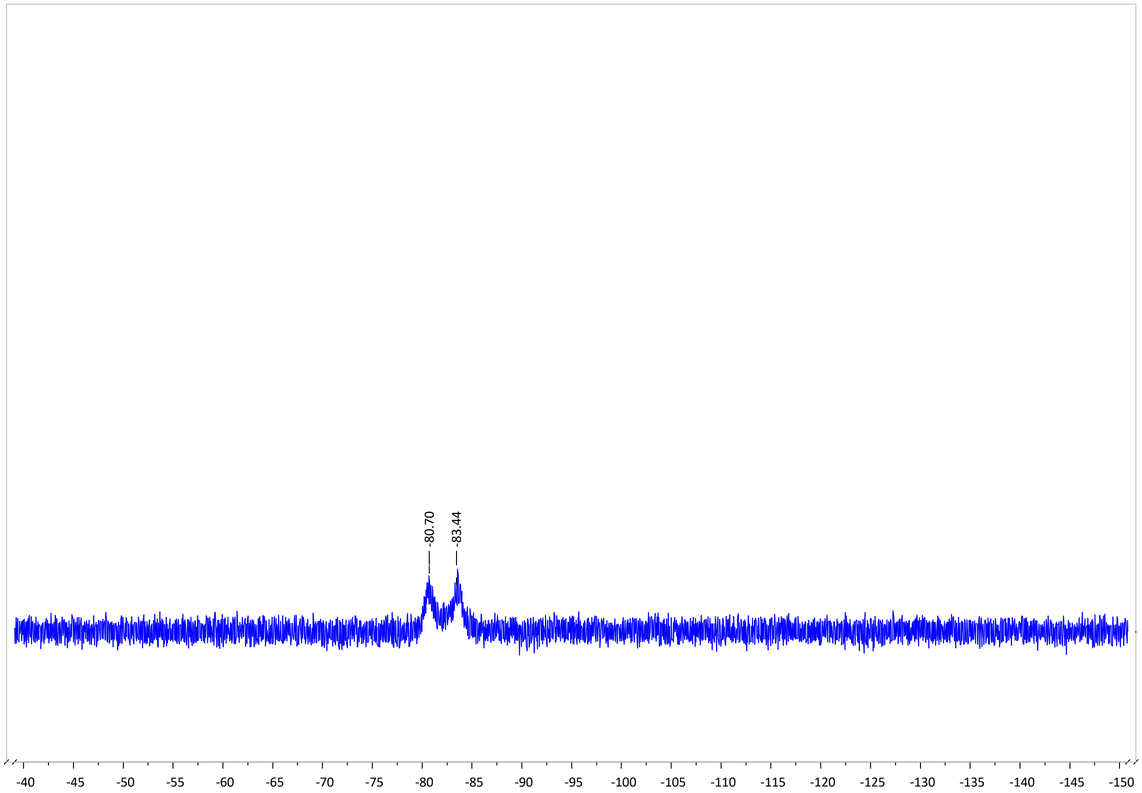

Figure S49. ^{31}P-NMR spectrum of [Ag(L^{2OiPr})(PTA)]NO_3 (7) in CD_3OD at 293 K.

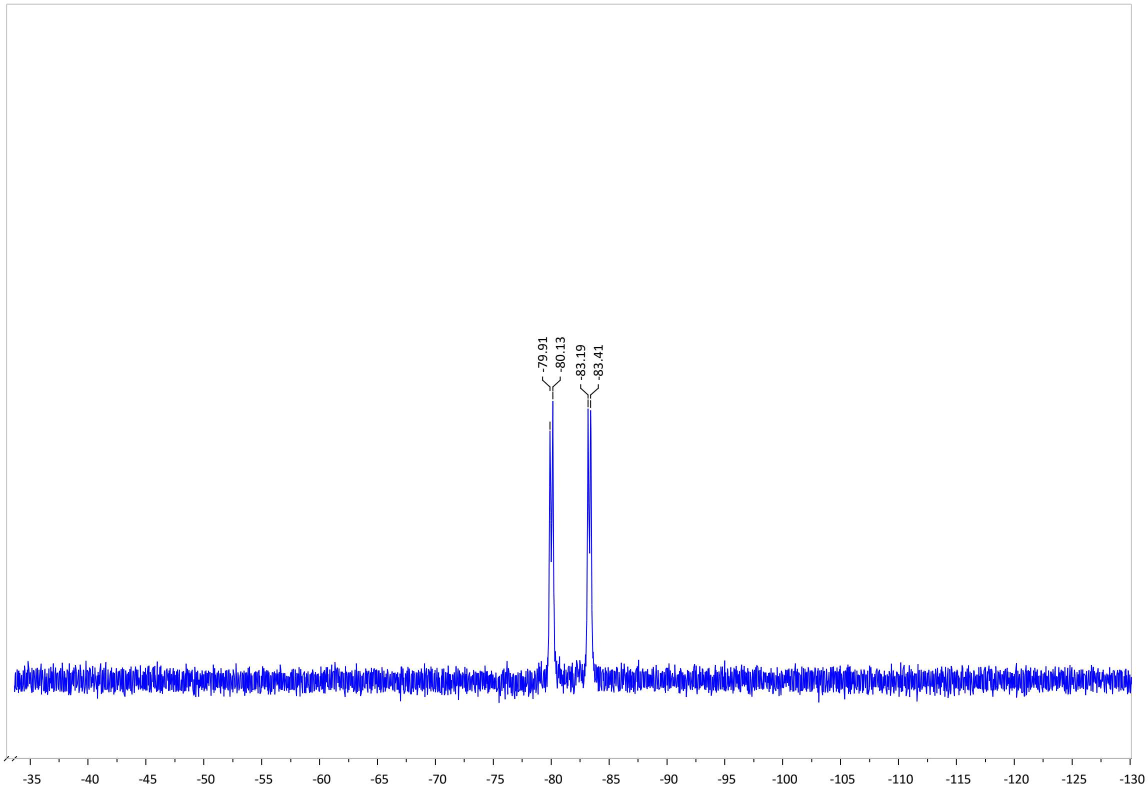

Figure S50. ^{31}P-NMR spectrum of [Ag(L^{2OiPr})(PTA)]NO_3 (7) in CD_3OD at 263 K.

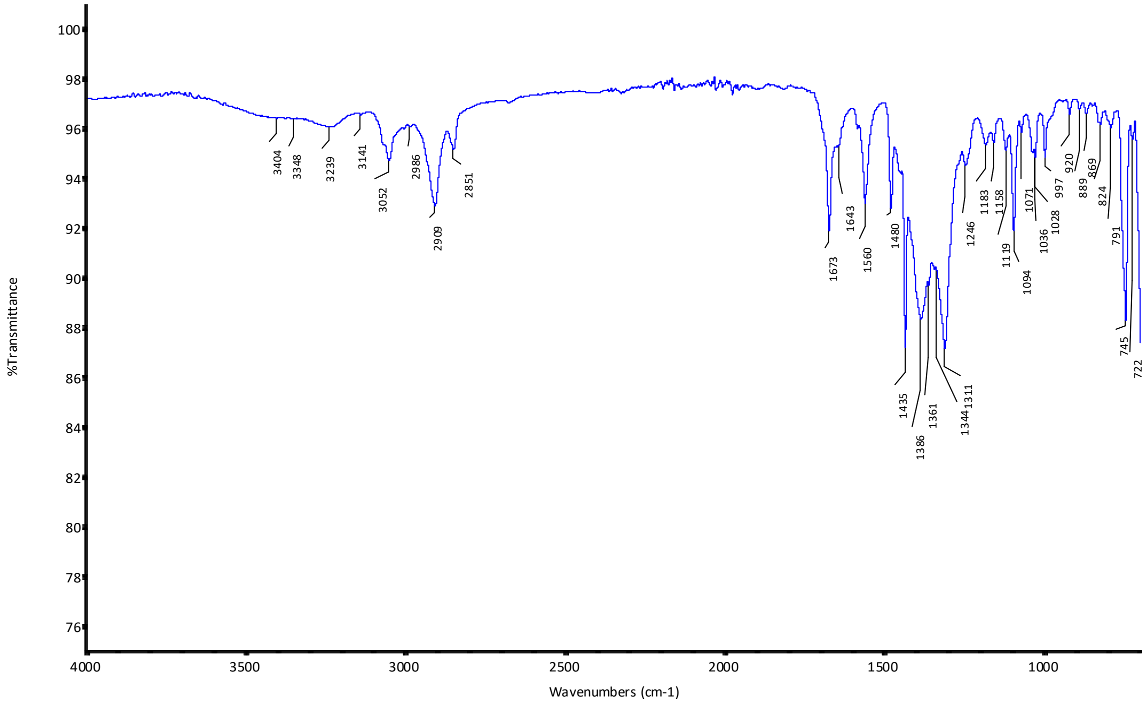

Figure S51. FT-IR spectrum of crystals of [Ag(L^{2Ad})(PPh$_3$)]NO$_3$ (3a).

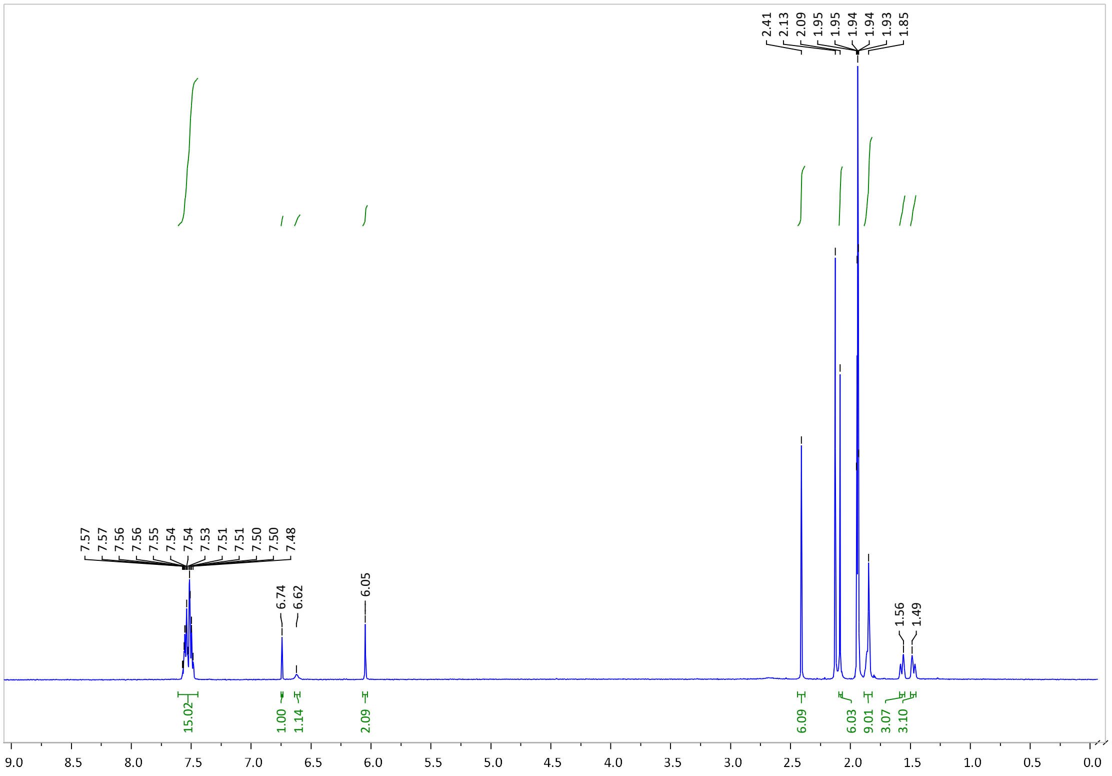

Figure S52. ^{1}H-NMR spectrum of crystals of [Ag(L^{2Ad})(PPh$_3$)]NO$_3$ (3a) in CD_3CN.

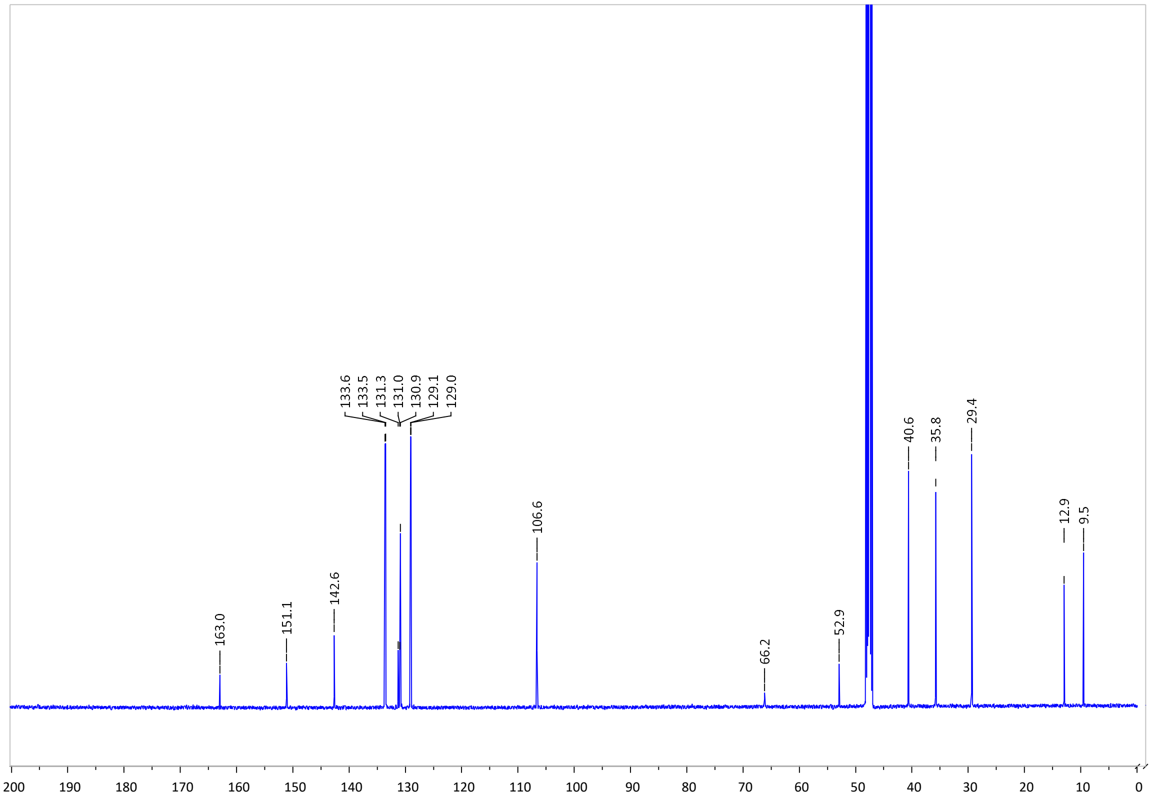

Figure S53. $^{13}C\{^{1}H\}$-NMR spectrum of crystals of $[Ag(L^{2Ad})(PPh_3)]NO_3$ (3a) in CD_3OD.

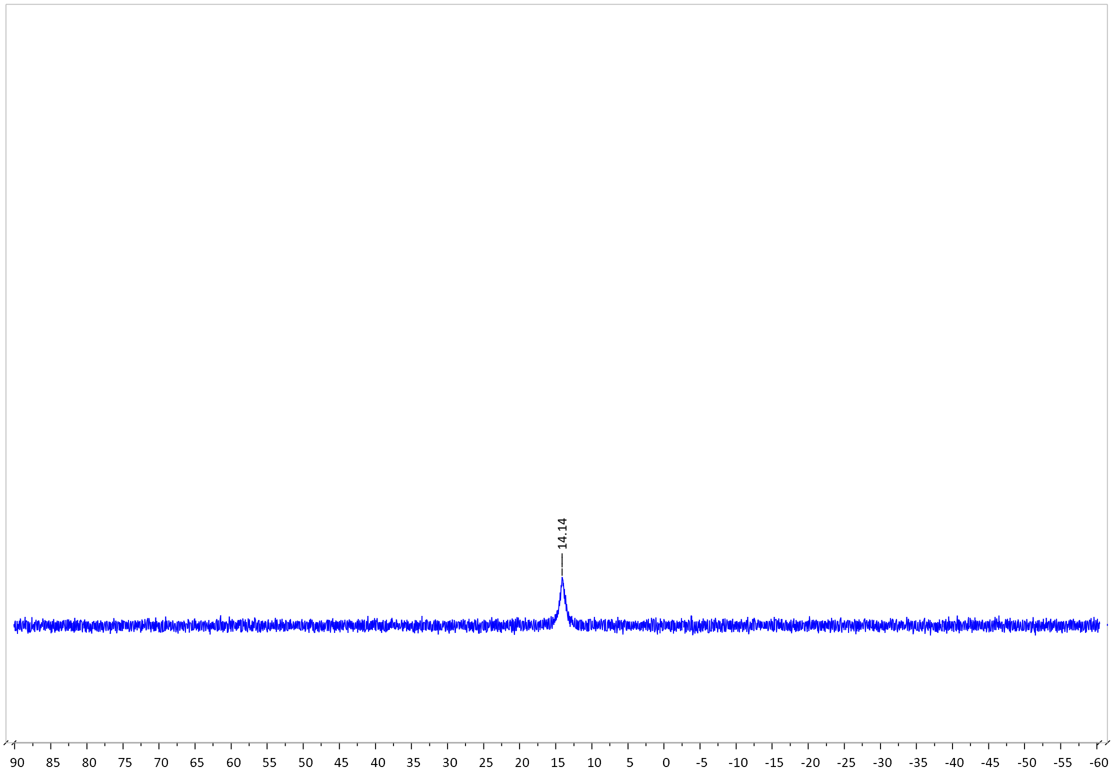

Figure S54. $^{31}P\{^{1}H\}$-NMR spectrum of crystals of $[Ag(L^{2Ad})(PPh_3)]NO_3$ (3a) in CD_3OD at 293 K.

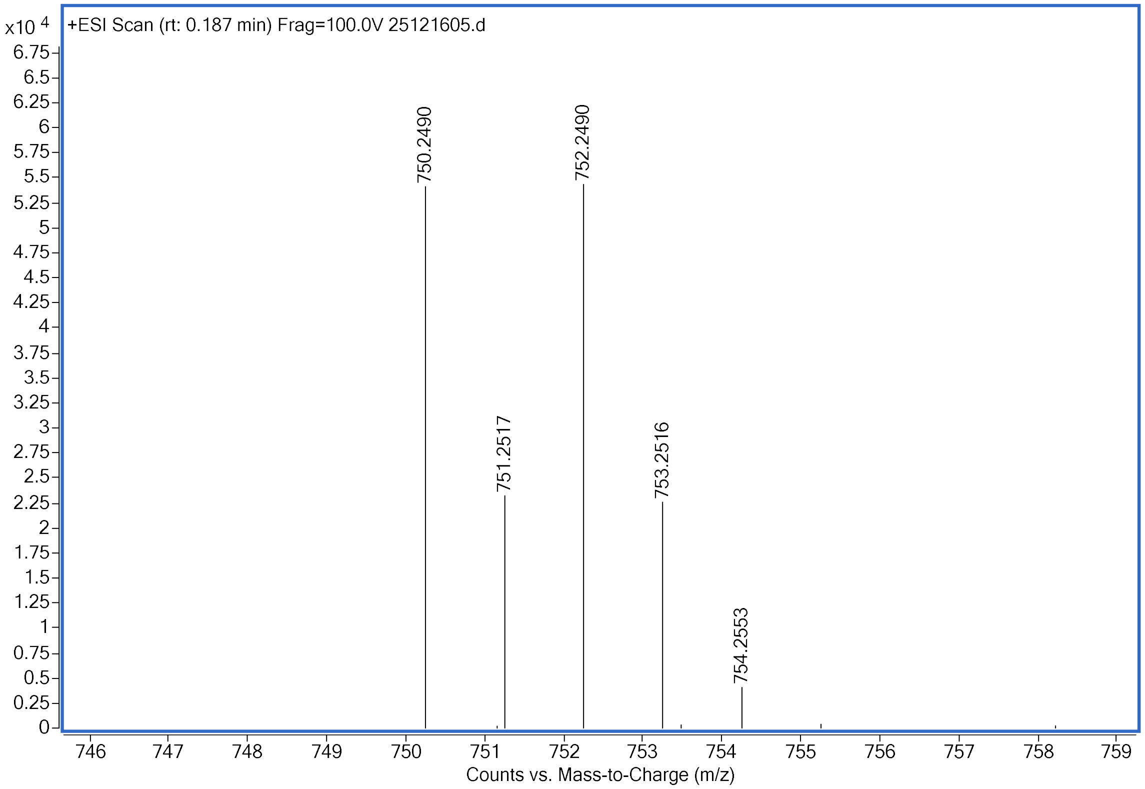

Figure S55. HR-MS spectrum of crystals of [Ag(L^{2Ad})(PPh_3)]NO_3 (3a), molecular monoisotopic mass ion peaks at m/z 750.2490 [$C_{40}H_{46}Ag^{106.9051}N_5OP$]$^+$ and 752.2490 [$C_{40}H_{46}Ag^{108.9048}N_5OP$]$^+$; HRMS-ESI(+) (Agilent 6545 – qTOF), [M]$^+$.

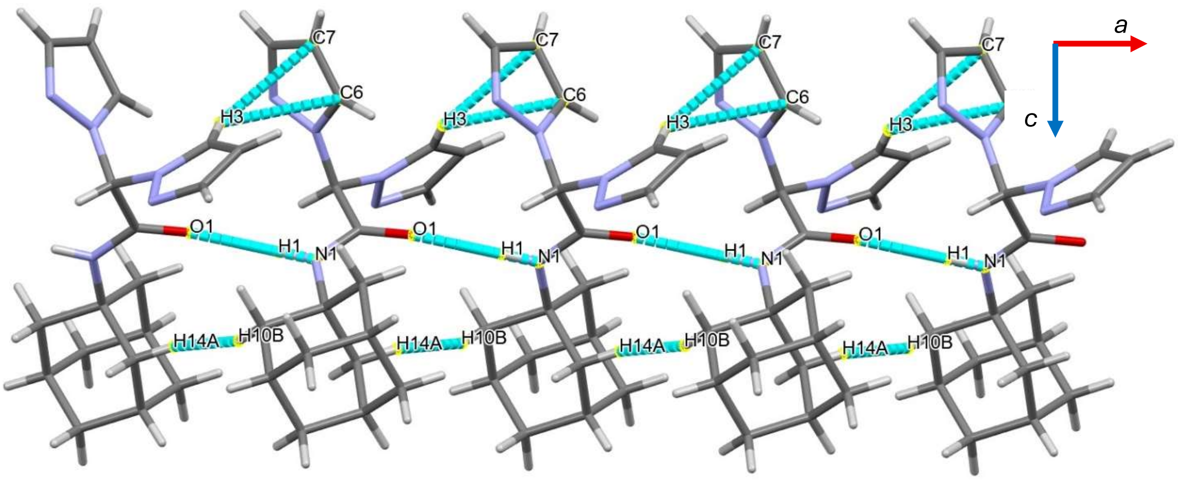

Figure S56. Packing diagram of intermolecular interactions for the ligand L^{Ad}. The N1-H1····O1 intermolecular hydrogen bond and the supporting interactions highlighted in cyan, viewed down the crystallographic b axis. The resulting motif propagates along the crystallographic a axis.

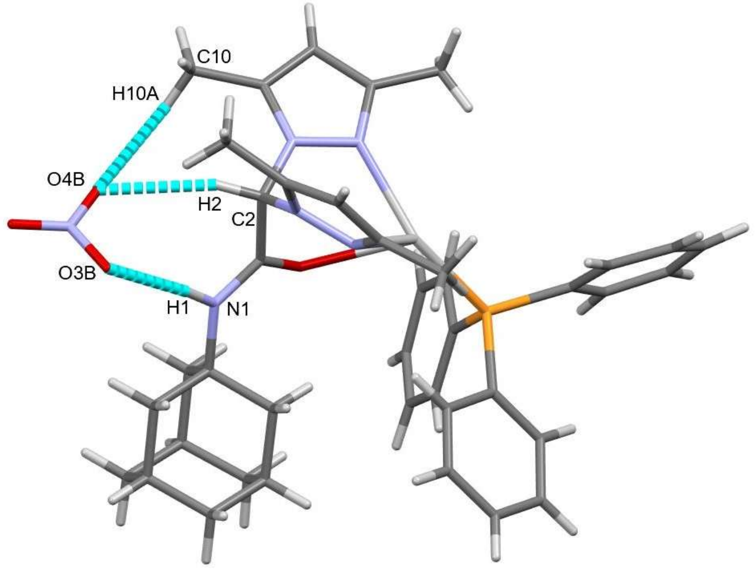

Figure S57. Pairing between the $[Ag(L^{2Ad})(PPh_3)]^+$ cation and the nitrate anion in complex (3a). The N1-H1····O3B hydrogen bond and the C2-H2····O4B, C10-H10A····O4B interactions highlighted in cyan.

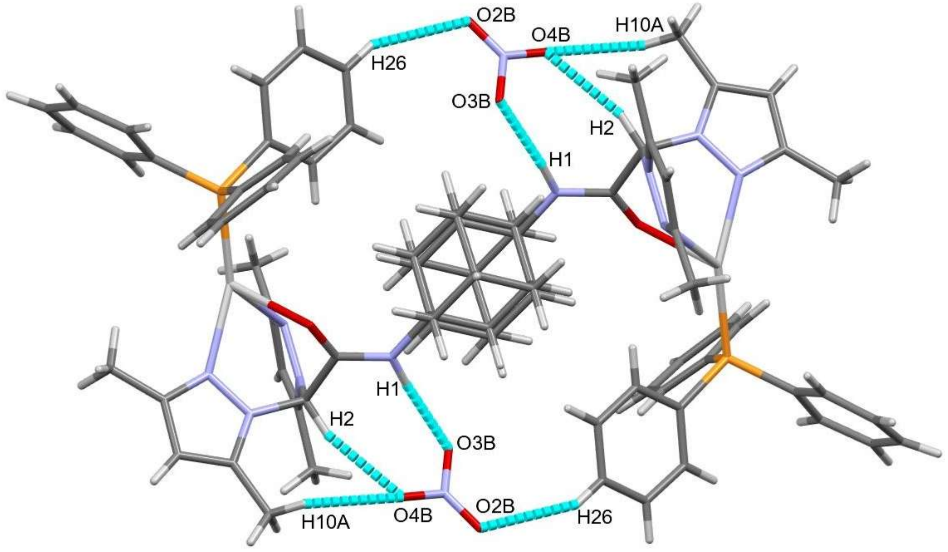

Figure S58. Cation/anion paired pairs in complex 3a. The involved H····O contacts highlighted in cyan.

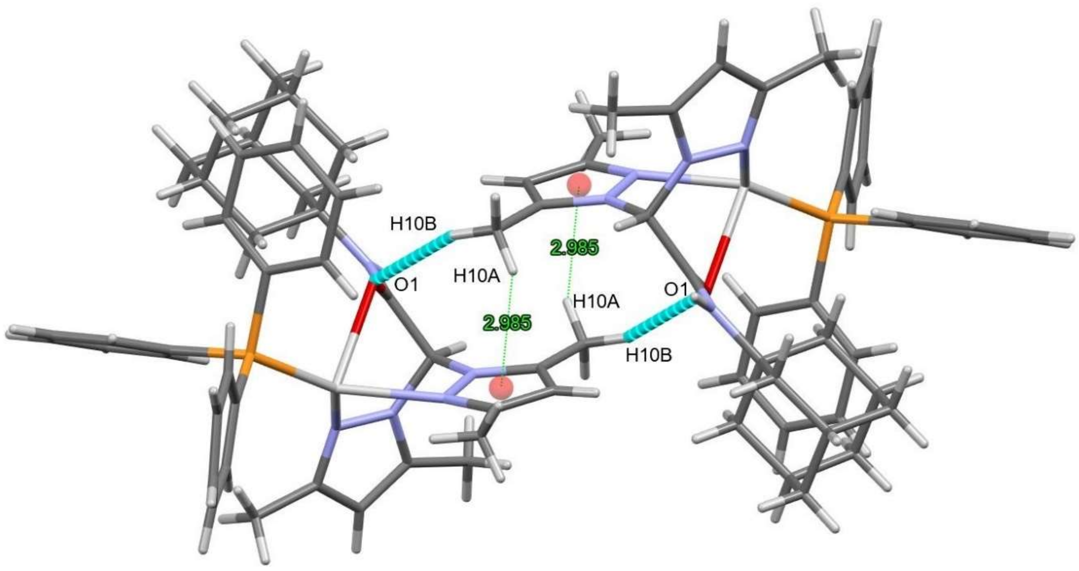

Figure S59. Paired cations in complex 3a. The involved H10B····O1 contacts highlighted in cyan; the interaction and mutual distance of H10A with the centroid of N2/C5 ring in green.

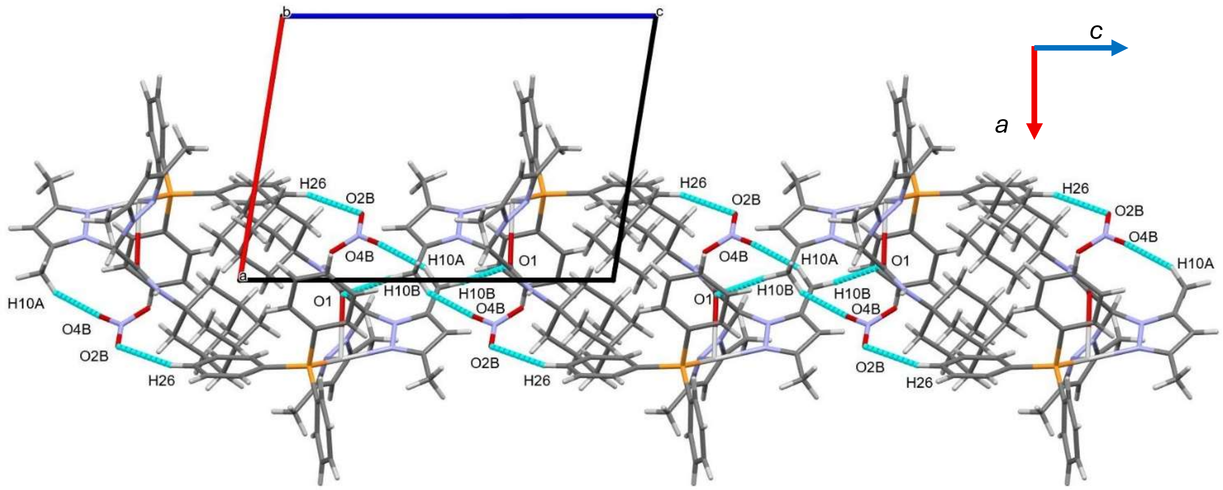

Figure S60. One-dimensional chain of cation/anion paired pairs originated by H10B···O1 and H26····O2B contacts in complex 3a. The motif runs along the crystallographic c axis. Contacts highlighted in cyan

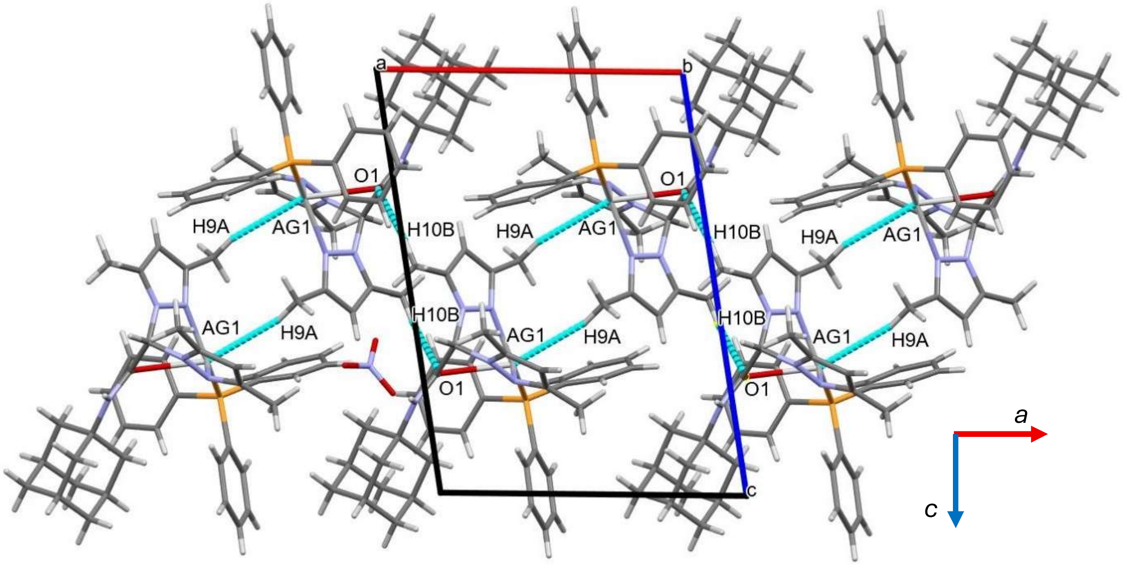

Figure S61. One-dimensional chain of cations originated by H10B···O1 and H9A····Ag1 contacts in complex 3a. The motif runs along the crystallographic a axis. Contact highlighted in cyan

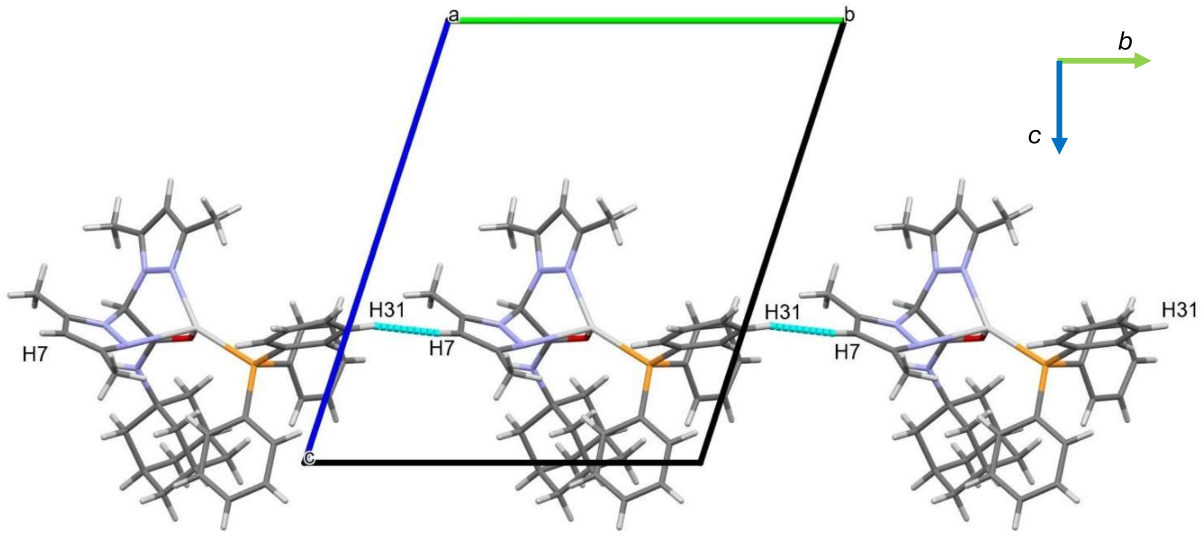

Figure S62. One-dimensional chain originated by H7····H31 contact in complex 3a. The motif runs along the crystallographic b axis. Contact highlighted in cyan.

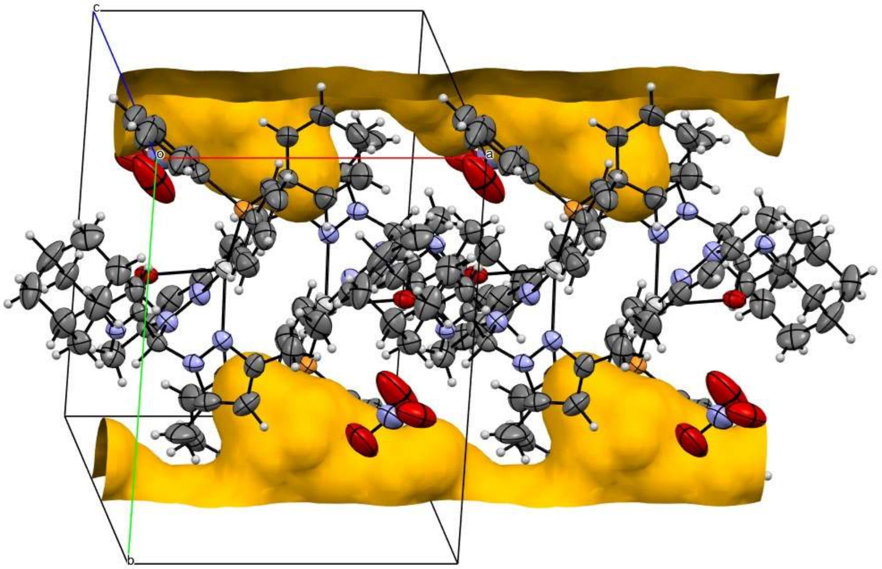

Figure S63. A representation of the unit cell voids hosting the crystallization acetonitrile molecules in complex 3a. The view is taken roughly down the crystallographic c axis.

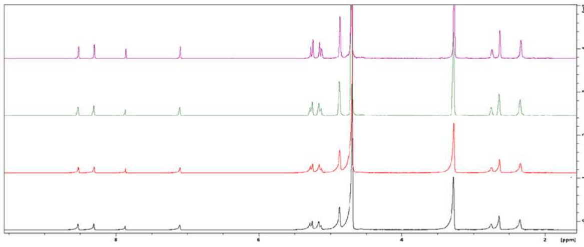

$[Ag(L^{Ad})(PPh_3)_2]NO_3$ (1)

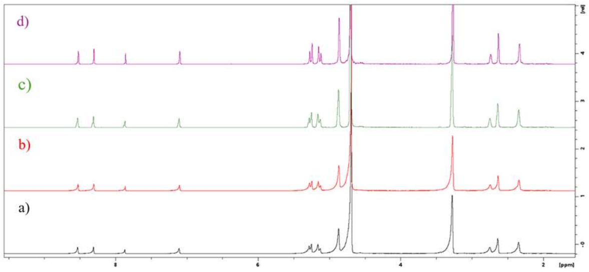

$[Ag(L^{Ad})(PTA)_2]NO_3$ (2)

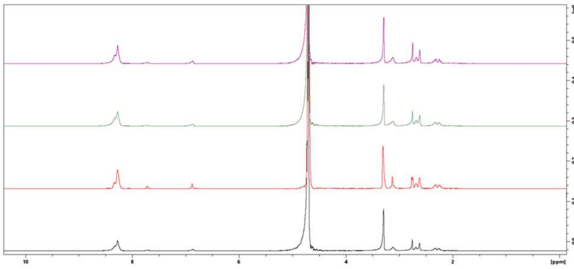

$[Ag(L^{2Ad})(PPh_3)]NO_3$ (3)

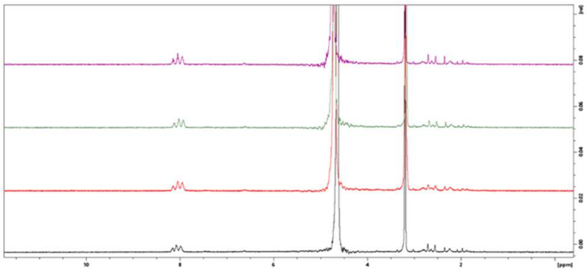

$[Ag(L^{2Ad})(PPh_3)_2]NO_3$ (4)

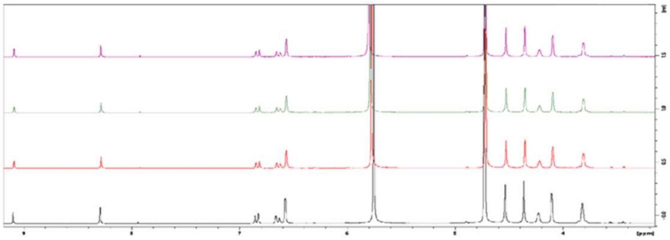

$[Ag(L^{2Ad})(PTA)]NO_3$ (5)

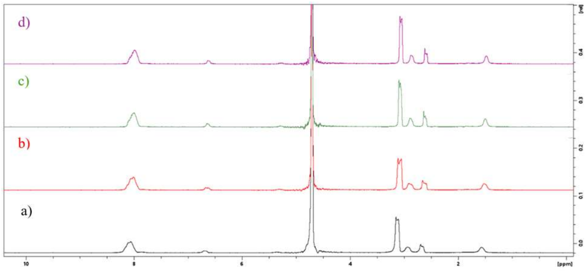

$[Ag(L^{2OiPr})(PPh_3)]NO_3$ (6)

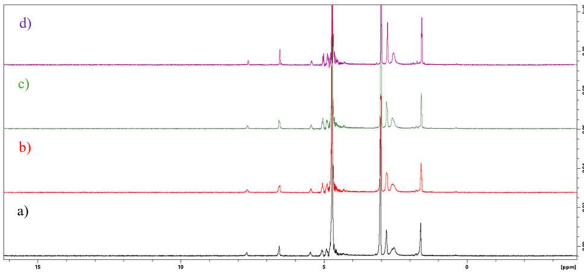

$[Ag(L^{2OiPr})(PTA)]NO_3$ (7)

Figure S64. Stability studies: ^{1}H-NMR spectra of compounds 1-7 in DMSO-d_6 (~0.4 mL), followed by dilution with D_2O to a final volume of 0.5 mL. Spectra were recorded at time points: a) t = 0; b) t = 24 h; c) t = 48 h; d) t = 72 h.

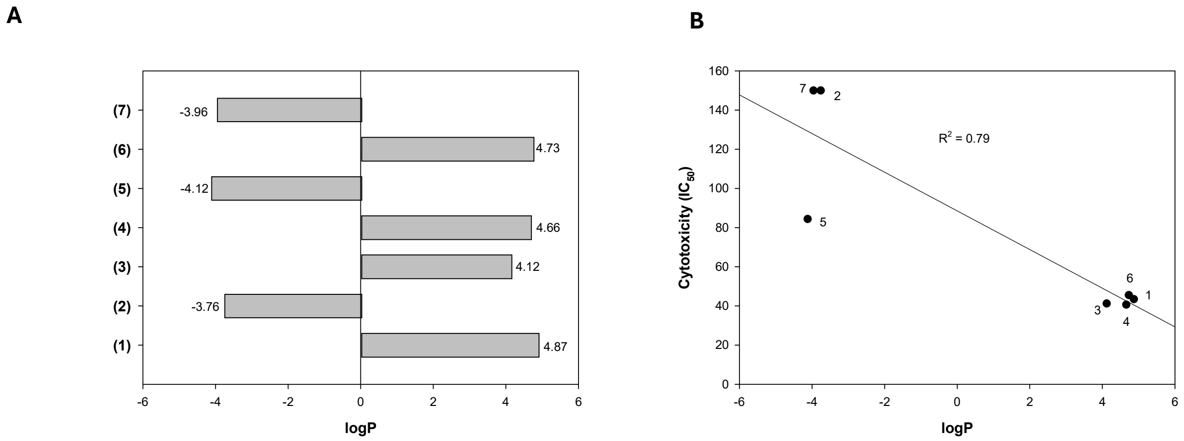

Figure S65. LogP values (A) and correlation with cytotoxicity in 3D system (B). To determine the logP the shake-flask method was used. Octanol-saturated water (OSW) and water-saturated octanol (WSO) were prepared using analytical grade reagents. Compounds were first dissolved in DMSO and then vortexed for 30 min at room temperature to establish the partition equilibrium. To separate the phases, centrifugation was performed at 3000 g for 5 min. The aqueous layer was carefully separated from the octanol layer for Ag analysis. Silver was quantified by GF-AAS. Partition coefficients were calculated using the equation logP = log([Ag]WSO/[Ag]OSW).

Table S1. List of crystallographic data for the ligand L^{Ad}.

Fractional Atomic Coordinates (×10^4) and Equivalent Isotropic Displacement Parameters (Å^2×10^3) for the ligand L^{Ad}. U_{eq} is defined as 1/3 of the trace of the orthogonalised U_{IJ} tensor.

Atom	x	y	z	U(eq)
O1	3184.1(8)	5042.5(10)	3649.3(3)	48.9(3)
N1	5364.5(10)	5499.2(11)	3802.3(3)	39.2(3)
N2	3892.0(11)	2580.8(11)	3307.3(3)	40.1(3)
N3	4019.6(14)	1307.0(13)	3467.4(3)	58.2(3)
N4	5360.8(10)	4256.0(11)	3032.9(3)	39.8(3)
N5	6307.3(12)	3547.4(14)	2841.6(3)	50.9(3)
C1	4394.3(12)	4821.2(13)	3623.3(3)	35.8(3)
C2	4919.4(12)	3648.9(13)	3368.0(3)	36.8(3)
C3	2726.2(15)	2692.4(16)	3119.1(4)	50.7(3)
C4	2064.0(17)	1440.5(18)	3155.9(4)	58.6(4)
C5	2897(2)	635.6(18)	3372.1(5)	66.6(5)
C6	4804.8(15)	5333.0(16)	2842.2(4)	48.3(3)

Fractional Atomic Coordinates (×10^4) and Equivalent Isotropic Displacement Parameters (Å^2×10^3) for the ligand L^{Ad}. U_{eq} is defined as 1/3 of the trace of the orthogonalised U_{IJ} tensor.

Atom	x	y	z	U(eq)
C7	5425.6(17)	5344.1(19)	2517.4(4)	57.6(4)
C8	6336.1(17)	4230.4(19)	2530.5(4)	58.5(4)
C9	5144.7(11)	6579.4(13)	4083.0(3)	35.8(3)
C10	6541.2(13)	7118.4(15)	4203.3(4)	45.2(3)
C11	6381.3(15)	8218.4(16)	4502.8(4)	52.7(4)
C12	5669.1(18)	7543(2)	4822.5(4)	59.5(4)
C13	4273.0(16)	7019.4(17)	4703.6(4)	51.5(4)
C14	4435.1(15)	5911.0(15)	4405.9(4)	44.6(3)
C15	4313.3(13)	7832.1(14)	3941.8(3)	41.6(3)
C16	4153.2(14)	8929.8(15)	4242.1(4)	48.3(3)
C17	3436.4(16)	8254.8(18)	4560.5(4)	53.9(4)
C18	5544.0(17)	9458.1(16)	4361.7(5)	56.8(4)

Anisotropic Displacement Parameters (Å^2×10^3) for the ligand L^{Ad}. The Anisotropic displacement factor exponent takes the form: $-2\pi^2[h^2a^{*2}U_{11}+2hka^*b^*U_{12}+\ldots]$.

Atom	U_{11}	U_{22}	U_{33}	U_{23}	U_{13}	U_{12}
O1	24.3(5)	63.6(6)	58.9(6)	-15.8(5)	4.1(4)	-3.2(4)
N1	22.7(5)	48.4(6)	46.7(6)	-10.0(4)	3.3(4)	1.1(4)
N2	40.0(6)	39.4(5)	41.0(5)	-0.9(4)	0.7(4)	-2.2(4)
N3	74.2(9)	46.0(6)	54.3(7)	9.1(5)	-4.4(6)	-6.0(6)
N4	32.4(5)	46.6(6)	40.3(5)	-3.3(4)	6.3(4)	-1.1(4)
N5	40.7(6)	61.8(7)	50.4(6)	-8.2(5)	12.5(5)	1.5(5)
C1	27.3(6)	43.4(6)	36.8(6)	-0.7(5)	3.4(4)	-2.8(5)
C2	29.5(6)	43.2(6)	37.7(6)	-1.0(5)	0.4(5)	1.3(5)
C3	42.4(8)	51.1(8)	58.4(8)	-5.1(6)	-5.7(6)	-2.7(6)
C4	51.0(9)	63.5(9)	61.4(9)	-17.6(7)	5.4(7)	-17.5(7)
C5	85.9(13)	50.2(9)	63.9(9)	2.1(7)	5.0(9)	-21.5(8)
C6	42.1(8)	51.1(7)	51.8(7)	4.5(6)	1.4(6)	-2.6(6)
C7	57.7(9)	68.7(10)	46.2(7)	9.6(7)	0.4(6)	-14.0(8)
C8	53.6(9)	77.7(10)	44.6(7)	-7.4(7)	14.7(6)	-11.7(8)
C9	27.3(6)	40.9(6)	39.3(6)	-5.6(5)	3.0(4)	-0.1(5)
C10	29.1(7)	51.0(7)	55.5(8)	-10.6(6)	0.5(5)	-0.6(5)
C11	39.2(8)	58.2(8)	60.7(8)	-18.6(7)	-3.2(6)	-6.2(6)
C12	63.0(10)	69.9(10)	45.6(8)	-13.6(7)	-6.9(7)	4.4(8)
C13	54.4(9)	60.9(8)	39.5(7)	-0.1(6)	11.3(6)	-0.3(7)
C14	42.2(7)	46.5(7)	45.3(7)	2.1(5)	4.0(6)	-3.1(6)
C15	34.6(7)	47.5(7)	42.7(7)	1.0(5)	4.0(5)	3.6(5)
C16	45.4(8)	44.0(7)	55.5(8)	-2.7(6)	7.3(6)	9.5(6)
C17	44.9(8)	62.9(9)	54.1(8)	-10.3(7)	14.1(6)	6.8(7)
C18	58.3(9)	46.4(8)	65.8(9)	-15.0(7)	9.7(7)	-5.7(7)

Bond Lengths for the ligand L^{Ad}.

Atom	Atom	Length/Å	Atom	Atom	Length/Å
O1	C1	1.2196(15)	C6	C7	1.362(2)
N1	C1	1.3267(16)	C7	C8	1.385(3)
N1	C9	1.4782(15)	C9	C10	1.5338(17)
N2	N3	1.3478(16)	C9	C14	1.5340(17)
N2	C2	1.4477(16)	C9	C15	1.5307(17)
N2	C3	1.3465(18)	C10	C11	1.5333(19)
N3	C5	1.323(2)	C11	C12	1.529(2)
N4	N5	1.3583(15)	C11	C18	1.524(2)
N4	C2	1.4446(15)	C12	C13	1.527(2)
N4	C6	1.3536(18)	C13	C14	1.5341(19)
N5	C8	1.326(2)	C13	C17	1.523(2)
C1	C2	1.5521(16)	C15	C16	1.5340(18)
C3	C4	1.359(2)	C16	C17	1.527(2)
C4	C5	1.375(3)	C16	C18	1.525(2)

Bond Angles for the ligand L^{Ad}.

Atom	Atom	Atom	Angle/˚	Atom	Atom	Atom	Angle/˚
C1	N1	C9	125.25(10)	N1	C9	C10	107.25(9)
N3	N2	C2	119.41(11)	N1	C9	C14	109.98(10)
C3	N2	N3	112.04(12)	N1	C9	C15	111.92(10)
C3	N2	C2	128.46(11)	C10	C9	C14	109.02(10)
C5	N3	N2	103.60(13)	C15	C9	C10	108.79(11)
N5	N4	C2	118.24(11)	C15	C9	C14	109.80(10)
C6	N4	N5	111.95(11)	C11	C10	C9	109.72(11)
C6	N4	C2	128.77(11)	C12	C11	C10	109.57(13)
C8	N5	N4	103.84(12)	C18	C11	C10	109.27(12)
O1	C1	N1	125.60(11)	C18	C11	C12	109.62(13)
O1	C1	C2	120.39(10)	C13	C12	C11	109.40(12)
N1	C1	C2	114.01(10)	C12	C13	C14	109.20(12)
N2	C2	C1	110.72(9)	C17	C13	C12	109.82(14)
N4	C2	N2	111.10(10)	C17	C13	C14	109.32(12)
N4	C2	C1	110.58(10)	C13	C14	C9	109.70(11)
N2	C3	C4	106.86(14)	C9	C15	C16	109.36(10)
C3	C4	C5	104.71(14)	C17	C16	C15	109.61(12)
N3	C5	C4	112.78(15)	C18	C16	C15	109.52(11)
N4	C6	C7	106.67(14)	C18	C16	C17	109.55(13)
C6	C7	C8	105.17(14)	C13	C17	C16	109.66(12)
N5	C8	C7	112.35(13)	C11	C18	C16	109.51(12)

Table S2. List of crystallographic data for the complex $[Ag(L^{2Ad})(PPh_3)]NO_3$ (3).

Fractional Atomic Coordinates (×10^4) and Equivalent Isotropic Displacement Parameters (Å^2×10^3) for the complex $[Ag(L^{2Ad})(PPh_3)]NO_3$ (3). U_{eq} is defined as 1/3 of the trace of the orthogonalised U_{IJ} tensor.

Atom	x	y	z	U(eq)
Ag1	7001.8(2)	6157.1(2)	6950.8(2)	56.93(9)
P1	6750.6(6)	7924.3(5)	7775.2(6)	54.21(18)
O1	9434.1(18)	6095.4(14)	7181.3(12)	50.2(4)
N1	10393.4(19)	4986.8(16)	7867.5(14)	42.1(4)
N2	7431(2)	5056.4(19)	5628.8(14)	49.2(5)
N3	8356(2)	4387.1(17)	5658.5(13)	45.0(5)
N4	7020(2)	4538.5(17)	7347.8(14)	45.5(5)
N5	7746(2)	3808.5(16)	6883.1(14)	43.3(4)
C1	9592(2)	5198.3(19)	7230.1(15)	38.6(5)
C2	8810(2)	4182.8(19)	6510.0(16)	41.2(5)
C3	7222(3)	5076(3)	4767.1(19)	58.7(7)
C4	8012(3)	4422(3)	4253.8(19)	66.5(8)
C5	8729(3)	3989(2)	4826.4(18)	54.9(6)
C6	6021(3)	3959(3)	7465.3(19)	54.9(7)
C7	6098(4)	2877(3)	7077(3)	75.9(10)
C8	7193(3)	2790(2)	6700(2)	63.5(8)
C9	6262(4)	5747(4)	4491(3)	87.0(12)
C10	9710(3)	3222(3)	4651(2)	70.2(8)
C11	5005(3)	4483(4)	7964(2)	76.5(10)
C12	7777(6)	1831(3)	6184(4)	113.3(17)
C13	11114(2)	5806(2)	8695.7(16)	40.8(5)
C14	11790(3)	5195(3)	9288(2)	63.6(8)
C15	12520(3)	5998(3)	10186(2)	78.7(10)
C16	11620(4)	6677(4)	10704(2)	87.1(12)
C17	10949(4)	7275(3)	10129(2)	76.4(10)
C18	10197(3)	6484(3)	9232.0(19)	57.1(7)
C19	12121(3)	6527(3)	8464(2)	65.0(8)
C20	12861(4)	7336(4)	9366(3)	88.7(13)
C21	13523(3)	6705(4)	9948(3)	93.0(13)
C22	11907(5)	8002(3)	9883(3)	93.1(13)
C23	6703(2)	8128(2)	8984(2)	55.4(7)
C24	7054(3)	7329(3)	9357(3)	65.3(8)
C25	7089(4)	7468(3)	10278(3)	77.2(9)
C26	6754(3)	8394(3)	10840(3)	78.6(10)
C27	6394(3)	9194(3)	10479(3)	78.0(10)
C28	6369(3)	9072(3)	9562(3)	66.7(8)
C29	5393(3)	8542(2)	7392(2)	53.4(6)
C30	5481(4)	9538(4)	7286(5)	125(2)
C31	4395(5)	9948(4)	6982(5)	134(3)
C32	3233(4)	9384(3)	6797(3)	86.8(11)

Fractional Atomic Coordinates (×10^4) and Equivalent Isotropic Displacement Parameters (Å^2×10^3) for the complex [Ag(L^{2Ad})(PPh_3)]NO_3 (3). U_{eq} is defined as 1/3 of the trace of the orthogonalised U_{IJ} tensor.

Atom	x	y	z	U(eq)
C33	3117(4)	8461(4)	7001(4)	96.9(14)
C34	4196(3)	8022(3)	7267(3)	84.1(12)
C35	8151(3)	8803(2)	7764(3)	68.5(9)
C36	8978(4)	9400(4)	8553(4)	101.7(15)
C37	10035(5)	10058(4)	8501(5)	124(2)
C38	10243(5)	10147(4)	7693(6)	117(2)
C39	9460(5)	9534(6)	6870(6)	131(2)
C40	8402(4)	8831(4)	6933(4)	104.2(15)
O2B	12504(4)	1961(4)	7017(4)	158.8(19)
O3B	11068(5)	2826(3)	7663(3)	145.2(17)
O4B	11375(7)	2854(6)	6422(4)	218(3)
N6	11693(4)	2528(3)	7020(3)	90.9(10)

Anisotropic Displacement Parameters (Å^2×10^3) for the complex [Ag(L^{2Ad})(PPh_3)]NO_3 (3). The Anisotropic displacement factor exponent takes the form: $-2\pi^2[h^2a^{*2}U_{11}+2hka^*b^*U_{12}+\ldots]$.

Atom	U_{11}	U_{22}	U_{33}	U_{23}	U_{13}	U_{12}
Ag1	56.47(14)	43.34(12)	67.16(15)	10.17(9)	7.01(9)	16.39(9)
P1	41.0(3)	36.9(3)	82.9(5)	13.9(3)	12.0(3)	8.8(3)
O1	57.9(10)	40.0(9)	49.9(10)	15.7(8)	-5.4(8)	5.4(8)
N1	42.5(10)	38.1(10)	43.1(10)	12.1(8)	-1.3(8)	5.1(8)
N2	49.6(12)	58.5(13)	38.6(10)	16.7(10)	-1.8(9)	8.3(10)
N3	49.4(11)	48.0(11)	33.5(10)	8.2(8)	1.8(8)	7.3(9)
N4	46.9(11)	48.0(11)	42.8(11)	15.4(9)	6.3(8)	7.4(9)
N5	49.7(11)	35.7(10)	41.9(10)	10.4(8)	2.2(8)	3.8(8)
C1	37.5(11)	41.6(12)	36.3(11)	11.5(9)	5.1(9)	6.3(9)
C2	44.9(12)	38.3(12)	37.0(11)	7.6(9)	2.3(9)	8.7(9)
C3	57.0(16)	72.9(19)	44.6(14)	24.9(14)	-7.3(12)	-2.9(14)
C4	75(2)	84(2)	34.7(13)	16.0(14)	0.8(13)	-6.6(17)
C5	59.2(16)	58.2(16)	38.6(13)	4.0(12)	8.2(11)	-4.3(13)
C6	47.9(14)	69.8(18)	47.5(14)	24.4(13)	-0.9(11)	-2.2(12)
C7	75(2)	65(2)	84(2)	24.0(18)	9.8(18)	-19.8(17)
C8	78(2)	40.7(14)	65.0(18)	12.4(13)	3.6(15)	-3.6(13)
C9	77(2)	117(3)	77(2)	57(2)	-13.9(18)	12(2)
C10	74(2)	73(2)	55.4(17)	1.6(15)	21.6(15)	9.5(16)
C11	50.6(17)	115(3)	69(2)	35(2)	12.9(15)	8.7(18)
C12	147(4)	37.3(17)	149(4)	9(2)	49(4)	5(2)
C13	34.8(11)	46.9(13)	38.7(11)	13.0(10)	1.7(9)	-0.1(9)
C14	59.5(17)	69.3(19)	58.0(17)	24.7(15)	-13.7(13)	3.4(14)
C15	67(2)	102(3)	57.6(18)	27.3(19)	-22.4(15)	-4.6(19)
C16	77(2)	123(3)	42.3(16)	10.2(19)	-2.4(15)	-19(2)
C17	70(2)	83(2)	55.2(18)	-7.5(16)	9.4(15)	4.1(17)
C18	47.1(14)	67.0(18)	49.0(14)	6.7(13)	5.2(11)	5.7(13)

Anisotropic Displacement Parameters (Å^2×10^3) for the complex [Ag(L^{2Ad})(PPh$_3$)]NO$_3$ (3). The Anisotropic displacement factor exponent takes the form: $-2\pi^2[h^2a^{*2}U_{11}+2hka^*b^*U_{12}+\ldots]$.

Atom	U_{11}	U_{22}	U_{33}	U_{23}	U_{13}	U_{12}
C19	56.0(16)	79(2)	54.5(16)	17.4(15)	10.2(13)	-18.6(15)
C20	75(2)	104(3)	70(2)	19(2)	3.4(18)	-49(2)
C21	46.2(17)	133(4)	74(2)	7(2)	-10.7(16)	-13(2)
C22	113(3)	64(2)	75(2)	-4.6(19)	-4(2)	-13(2)
C23	34.9(12)	44.0(14)	80.9(19)	11.3(13)	4.3(12)	5.9(10)
C24	48.8(15)	52.0(16)	92(2)	18.6(16)	6.3(15)	12.2(12)
C25	67(2)	74(2)	92(3)	31(2)	0.7(18)	12.4(17)
C26	56.4(18)	90(3)	80(2)	18(2)	0.8(16)	2.9(17)
C27	61.0(19)	70(2)	85(2)	-1.9(18)	5.6(17)	10.4(16)
C28	55.5(17)	49.4(16)	86(2)	8.3(15)	4.2(15)	11.8(13)
C29	46.5(14)	40.8(13)	73.8(18)	16.2(13)	13.9(12)	8.8(11)
C30	56(2)	76(3)	276(8)	97(4)	34(3)	15.2(18)
C31	82(3)	95(3)	276(8)	120(5)	53(4)	37(3)
C32	70(2)	80(2)	112(3)	33(2)	2(2)	28.8(19)
C33	57(2)	90(3)	138(4)	43(3)	-18(2)	-2.1(19)
C34	54.4(18)	67(2)	130(3)	45(2)	-12.8(19)	-5.0(15)
C35	46.1(15)	49.1(16)	114(3)	22.3(17)	27.1(17)	11.5(12)
C36	61(2)	78(3)	143(4)	-1(3)	29(2)	-13.2(19)
C37	71(3)	94(3)	186(6)	6(4)	44(3)	-18(2)
C38	71(3)	79(3)	206(7)	43(4)	47(4)	-2(2)
C39	89(3)	140(5)	209(7)	97(5)	66(4)	33(4)
C40	71(2)	110(4)	147(4)	60(3)	27(3)	7(2)
O2B	136(3)	159(4)	228(5)	102(4)	54(3)	89(3)
O3B	210(5)	92(2)	166(4)	57(3)	77(4)	67(3)
O4B	255(7)	308(8)	184(5)	151(6)	98(5)	198(7)
N6	86(2)	84(2)	123(3)	56(2)	21(2)	31.3(18)

Bond Lengths for the complex [Ag(L^{2Ad})(PPh$_3$)]NO$_3$ (3).

Atom	Atom	Length/Å	Atom	Atom	Length/Å
Ag1	P1	2.3417(7)	C14	C15	1.541(4)
Ag1	O1	2.5684(19)	C15	C16	1.498(6)
Ag1	N2	2.250(2)	C15	C21	1.514(6)
Ag1	N4	2.377(2)	C16	C17	1.496(6)
P1	C23	1.818(3)	C17	C18	1.541(4)
P1	C29	1.818(3)	C17	C22	1.504(6)
P1	C35	1.820(3)	C19	C20	1.551(5)
O1	C1	1.222(3)	C20	C21	1.531(7)
N1	C1	1.322(3)	C20	C22	1.533(7)
N1	C13	1.480(3)	C23	C24	1.387(4)
N2	N3	1.367(3)	C23	C28	1.394(4)
N2	C3	1.332(3)	C24	C25	1.379(5)
N3	C2	1.450(3)	C25	C26	1.368(6)

Bond Lengths for the complex [Ag(L^{2Ad})(PPh_3)]NO_3 (3).

Atom	Atom	Length/Å	Atom	Atom	Length/Å
N3	C5	1.361(3)	C26	C27	1.381(6)
N4	N5	1.367(3)	C27	C28	1.377(6)
N4	C6	1.323(4)	C29	C30	1.360(5)
N5	C2	1.452(3)	C29	C34	1.360(4)
N5	C8	1.352(3)	C30	C31	1.390(6)
C1	C2	1.556(3)	C31	C32	1.341(6)
C3	C4	1.393(5)	C32	C33	1.339(6)
C3	C9	1.492(5)	C33	C34	1.385(5)
C4	C5	1.368(5)	C35	C36	1.389(6)
C5	C10	1.491(5)	C35	C40	1.358(7)
C6	C7	1.379(5)	C36	C37	1.391(6)
C6	C11	1.496(5)	C37	C38	1.335(9)
C7	C8	1.363(5)	C38	C39	1.407(9)
C8	C12	1.498(5)	C39	C40	1.432(7)
C13	C14	1.530(4)	O2B	N6	1.176(5)
C13	C18	1.523(4)	O3B	N6	1.250(5)
C13	C19	1.524(4)	O4B	N6	1.151(6)

Bond Angles for the complex [Ag(L^{2Ad})(PPh_3)]NO_3 (3).

Atom	Atom	Atom	Angle/˚	Atom	Atom	Atom	Angle/˚
P1	Ag1	O1	104.23(4)	N1	C13	C18	110.2(2)
P1	Ag1	N4	132.69(6)	N1	C13	C19	112.1(2)
N2	Ag1	P1	146.04(6)	C18	C13	C14	108.8(2)
N2	Ag1	O1	73.89(7)	C18	C13	C19	110.2(2)
N2	Ag1	N4	81.15(8)	C19	C13	C14	108.6(2)
N4	Ag1	O1	81.93(6)	C13	C14	C15	109.8(3)
C23	P1	Ag1	113.84(9)	C16	C15	C14	110.5(3)
C23	P1	C35	104.51(16)	C16	C15	C21	110.0(4)
C29	P1	Ag1	119.15(10)	C21	C15	C14	108.3(3)
C29	P1	C23	104.80(13)	C17	C16	C15	110.2(3)
C29	P1	C35	104.76(14)	C16	C17	C18	110.6(3)
C35	P1	Ag1	108.50(10)	C16	C17	C22	110.1(3)
C1	O1	Ag1	105.96(15)	C22	C17	C18	108.1(3)
C1	N1	C13	124.8(2)	C13	C18	C17	109.6(2)
N3	N2	Ag1	119.07(14)	C13	C19	C20	109.0(2)
C3	N2	Ag1	132.8(2)	C21	C20	C19	108.8(4)
C3	N2	N3	105.6(2)	C21	C20	C22	110.0(3)
N2	N3	C2	119.95(19)	C22	C20	C19	108.8(3)
C5	N3	N2	111.6(2)	C15	C21	C20	109.3(3)
C5	N3	C2	128.5(2)	C17	C22	C20	109.9(3)
N5	N4	Ag1	115.11(15)	C24	C23	P1	118.9(2)
C6	N4	Ag1	126.76(18)	C24	C23	C28	118.4(3)
C6	N4	N5	105.4(2)	C28	C23	P1	122.7(3)

Bond Angles for the complex [Ag(L^{2Ad})(PPh_3)]NO_3 (3).

Atom	Atom	Atom	Angle/˚	Atom	Atom	Atom	Angle/˚
N4	N5	C2	119.33(19)	C25	C24	C23	121.0(3)
C8	N5	N4	111.2(2)	C26	C25	C24	120.3(4)
C8	N5	C2	128.3(2)	C25	C26	C27	119.4(4)
O1	C1	N1	125.4(2)	C28	C27	C26	120.9(3)
O1	C1	C2	120.4(2)	C27	C28	C23	120.0(3)
N1	C1	C2	114.2(2)	C30	C29	P1	124.3(3)
N3	C2	N5	110.63(19)	C30	C29	C34	116.9(3)
N3	C2	C1	111.77(19)	C34	C29	P1	118.6(2)
N5	C2	C1	110.01(19)	C29	C30	C31	120.6(4)
N2	C3	C4	109.8(3)	C32	C31	C30	121.3(4)
N2	C3	C9	120.0(3)	C33	C32	C31	118.4(4)
C4	C3	C9	130.2(3)	C32	C33	C34	120.3(4)
C5	C4	C3	107.5(2)	C29	C34	C33	121.7(3)
N3	C5	C4	105.5(3)	C36	C35	P1	123.0(3)
N3	C5	C10	123.4(3)	C40	C35	P1	117.2(3)
C4	C5	C10	131.1(3)	C40	C35	C36	119.8(4)
N4	C6	C7	110.4(3)	C35	C36	C37	120.4(6)
N4	C6	C11	121.2(3)	C38	C37	C36	120.2(6)
C7	C6	C11	128.5(3)	C37	C38	C39	121.6(5)
C8	C7	C6	107.2(3)	C38	C39	C40	117.4(6)
N5	C8	C7	105.8(3)	C35	C40	C39	120.4(6)
N5	C8	C12	122.4(3)	O2B	N6	O3B	123.7(5)
C7	C8	C12	131.8(3)	O4B	N6	O2B	123.4(5)
N1	C13	C14	106.8(2)	O4B	N6	O3B	112.8(4)

Table S3. Main nonbonding interactions (Å and degrees) for L^{Ad}.

Donor (D)[a]	Contact (C)	Acceptor (A)	C····A	D····A	D–C–A [b]	Symmetry
N1	H1	O1	2.03	2.90	175.9	–1/2+x, –y, z
C3	H3	N4/C8[c]	2.73	3.58	129.7	–1/2+x, –y, z
C14	H14A	H10B	2.27	3.15	147.3	–1/2+x, –y, z

[a] Atom bound to contact atom; [b] Donor–Contact–Acceptor angle; [c] Geometric centroid of the N4/C8 pyrazolyl ring; listed contacts at least 0.05 Å shorter than the sum of the pertinent van der Waals radii.

The most efficient intermolecular interaction of L^{Ad} is the hydrogen bond connecting the H1 hydrogen and the O1 oxygen of a nearby molecule at -1/2 + x, - y, z (N1····O1 distance of 2.90 Å and N1-H1····O1 angle of 175.9°), which is strong enough to create a one-dimensional motif propagating along the crystallographic a axis (Figure S56). This interaction is supported by two other contacts involving the H3 atom and the π-electron density of the N4/C8 pyrazolyl ring of another unit, and an 'hydrophobic' H····H contact between H14A and H10B of two proximal adamantane residues (both contacts again at -1/2 + x, - y, z).

Table S4. Main nonbonding interactions (Å and degrees) for the complex $[Ag(L^{2Ad})(PPh_3)]NO_3$ (3).

Donor (D)[a]	Contact (C)	Acceptor (A)	C····A	D····A	D–C–A [b]	Symmetry
N1	H1	O3B	2.05	2.90	171.0	x, y, z
C2	H2	O4B	2.41	3.33	156.1	x, y, z
C10	H10A	O4B	2.54	3.24	129.4	x, y, z
C10	H10B	O1	2.56	3.43	151.0	2–x, 1–y, 1–z
C26	H26	O2B	2.62	3.48	153.3	2–x, 1–y, 2–z
C10	H10A	N2/C5[c]	2.98	3.53	117.5	2–x, 1–y, 1–z
C7	H7	H31	2.32	3.23	164.5	x, –1+y, z
C9	H9A	Ag1	3.14	4.08	166.0	1–x, 1–y, 1–z

[a] Atom bound to contact atom; [b] Donor–Contact–Acceptor angle; [c] Geometric centroid of the N2/C5 pyrazolyl ring; listed contacts at least 0.05 Å shorter than the sum of the pertinent van der Waals radii.

The complex cation and the nitrate counter anion are put together by several nonbonding intreractions, basically hydrogen bonds, which define an articulated network of contacts. Within the asymmetric unit, the tightest link involves the H1 hydrogen and the O3B oxygen, (H1····O3B distance about 0.65 Å shorter than the sum of van der Waals radii) and it is supported by non-canonical H2····O4B, H10A····O4B hydrogen bonds. These interactions just pair the two ions and do not further propagate along the crystal (Figure S57). Similarly, the nitrate O2B atom in its turn engages the H26 atom of a second unit at 2–x, 1–y, 2–z; the nitrate ion paired to this second unit engages backward the H26 atom of the x, y, z unit, so that two cation/anion couples pair together (Figure S58). Another non-canonical hydrogen bond connects the amidic O1 oxygen and the H10B atom of a nearby unit at 2–x, 1–y, 1–z, while the H10A atom of this unit interacts with the π-electron density of the N2/C5 pyrazolyl ring, pairing two cations together (Figure S59).The H26····O2B, H10B····O1 and H10A····O4B contacts taken together create a one-dimensional motif of cation/anion paired pairs propagating along the crystallographic c axis (Figure S60). A van der Waals head-to-tail approach forming a dimeric pair of cations engages Ag1 and the H9A atom of a second unit at 1–x, 1–y, 1–z. Notably this interaction, together with the H10B····O1 contact, define another one-dimensional chain of cations, this time propagating along the crystallographic a axis, that criss-cross the former chain (Figure S61). The last contact can be defined 'hydrophobic' in type and it is a softer H7····H31 interaction (second unit at x, –1+y, z). This approach originates a third one-dimensional motif (Figure S62) running along the crystallographic b axis, intersecting the former two chains and finally creating a 3D contact network. The nonbonding contact network representation above does not highlight the important role of the crystallization acetonitrile molecules, sitting in the unit cell voids (highlighted in Figure S63) interspersed between the nitrate anions and the complex cations, whose presence in our view drives the tripod coordination of the L^{2Ad} ligand.
